# Comparative effects of plant-derived anthocyanin- and flavanol-rich supplements on recovery outcomes after exercise-induced muscle damage: a systematic review and network meta-analysis

**DOI:** 10.3389/fnut.2026.1857527

**Published:** 2026-08-14

**Authors:** Kai-Xuan Xu, Juan Chang, Yi-Jia Wu, Hui-Wen Chen, Jie-Wei Zheng, Chengcheng Liu

**Affiliations:** 1 College of Physical Education, Hunan Normal University, Changsha, Hunan, China; 2 Department of Physical Education, Dankook University, Yongin-si, Gyeonggi-do, Republic of Korea; 3 School of Physical Education, Changsha University, Changsha, China

**Keywords:** anthocyanins, exercise recovery, exercise-induced muscle damage, flavanols, network meta-analysis, plant-derived polyphenols

## Abstract

**Background:**

Exercise-induced muscle damage (EIMD) commonly follows strenuous exercise and is marked by delayed onset muscle soreness (DOMS), reduced muscle strength, and increased creatine kinase (CK) and C-reactive protein (CRP), which may impair recovery and subsequent training. Plant-derived polyphenols such as anthocyanins or flavanols are widely used to support recovery, but their comparative effects remain uncertain.

**Methods:**

We conducted a systematic review and network meta-analysis (NMA) following established methodological guidance. PubMed, Web of Science, CENTRAL, and Embase were searched from inception to January 2026 for randomized controlled trials in healthy adults with acute EIMD that compared plant-derived anthocyanin- or flavanol-rich supplements with placebo or control conditions. Primary outcomes were DOMS, muscle strength, CK, and CRP at 48 h post-exercise; 24 h and 72 h outcomes were secondary. Frequentist random-effects network meta-analysis was performed in Stata 19.5, with interventions ranked using SUCRA. Pairwise meta-analyses, subgroup analyses, and sensitivity analyses were conducted for the main 48 h outcomes. Risk of bias and certainty of evidence were assessed using RoB 2.0 and CINeMA.

**Results:**

Thirty trials involving 595 participants and eight supplement types were included. In the primary analysis, only pomegranate and tart cherry significantly reduced 48 h DOMS vs. the placebo node, ranking first and second, respectively. No intervention showed a clear advantage for 48 h muscle strength, CK, or CRP. Pairwise meta-analyses were generally consistent with the network findings. For secondary outcomes, tart cherry was the only supplement associated with significant DOMS reduction at both 24 h and 72 h. No consistent benefits were observed for muscle strength, CK, or CRP at these time points. Sensitivity analyses did not materially alter the conclusions, although some estimates varied by supplementation protocol, outcome definition, or comparator characteristics. Certainty of evidence was generally low.

**Conclusion:**

Plant-derived polyphenol supplements did not provide consistent benefits across all recovery outcomes after EIMD. Their main potential value appears to lie in reducing DOMS, with tart cherry showing the most consistent favorable pattern and pomegranate showing potential benefit. These supplements should therefore be considered as targeted options for subjective soreness relief rather than universal strategies for accelerating objective physiological recovery.

**Systematic review registration:** https://www.crd.york.ac.uk/PROSPERO/view/CRD420261340555, identifier CRD420261340555.

## Introduction

Exercise-induced muscle damage (EIMD) is a common response to high-intensity training and competition. It is usually caused by unaccustomed eccentric exercise and involves a series of changes, including mechanical disruption, inflammation, and oxidative stress (1, 2). After EIMD occurs, damaged muscle fibers may show increased membrane permeability, disturbed calcium balance, and breakdown of structural proteins, which can further trigger local sterile inflammation and excessive production of reactive oxygen species (1, 2). In practice, EIMD is often marked by delayed onset muscle soreness (DOMS), increased creatine kinase (CK) and lactate dehydrogenase (LDH), and reduced maximal voluntary isometric contraction (MVIC), explosive performance, and joint range of motion. These effects are usually most evident 24–72 h after exercise (2, 3). Because such impairments can reduce the quality of subsequent training and increase the risk of secondary injury, improving recovery after EIMD has become an important topic in sports medicine, rehabilitation, and sports nutrition (2, 4).

Among plant polyphenols, anthocyanins and flavanols are two of the most widely studied compounds in exercise recovery research (5, 6). Anthocyanins are mainly found in dark-colored fruits and vegetables such as tart cherries, blackcurrants, and blueberries, whereas flavanols are primarily derived from cocoa and green tea extracts (6, 7). Both are commonly used in sports nutrition interventions in the form of concentrated juices, freeze-dried powders, capsules, and standardized extracts. In exercise recovery studies, anthocyanin-containing supplements have mainly been investigated for reducing DOMS and supporting muscle function recovery, whereas flavanol-containing supplements have more often been examined for vascular, metabolic, and endurance-related outcomes (5, 8, 9). Their proposed mechanisms may therefore differ. Anthocyanin-containing supplements may contribute to post-exercise recovery by attenuating exercise-induced oxidative stress and modulating inflammation-related responses (10, 11). Flavanols, in contrast, may act more strongly through vascular pathways by enhancing nitric oxide-dependent vasodilation and improving vascular function and microcirculation (12, 13). Overall, both classes of supplements appear promising, but they may not provide the same benefits across outcomes.

Because anthocyanins and flavanols differ in source, biological action, and potential benefits, a systematic comparison within a single evidence framework is needed. In recent years, network meta-analysis (NMA) has become increasingly useful for evaluating EIMD recovery strategies because it can combine direct and indirect evidence, compare multiple interventions, and rank their relative effects (14). Most existing studies have focused on physical recovery methods, such as cold-water immersion, contrast water therapy, and cryotherapy, or on basic nutritional supplements, including protein, branched-chain amino acids, and β-hydroxy-β-methyl butyrate (HMB) (15, 16). Systematic reviews and meta-analyses of plant-derived interventions for exercise-induced muscle damage have also examined curcumin, tea-beverage leaf extracts, and broadly defined polyphenol-rich foods, juices, and concentrates (17–19). However, several limitations remain. First, previous reviews and meta-analyses have often pooled chemically diverse polyphenol interventions within broad categories, limiting subclass-specific interpretation (19–21). Second, most previous reviews focus on biochemical markers such as CK, LDH, and C-reactive protein (CRP), while giving less attention to more recovery-relevant functional outcomes such as MVIC, countermovement jump (CMJ), and DOMS (3, 4, 20). Third, variation in supplementation timing, treatment duration, and dietary control contributes to between-study heterogeneity and weakens the interpretation of findings (19). More refined comparisons of specific polyphenol subclasses are therefore still needed.

Although existing studies suggest that both anthocyanins and flavanols may help recovery after EIMD, important evidence gaps remain. Most previous reviews and meta-analyses have been limited to pairwise comparisons showing whether one class of polyphenols performs better than placebo, with few high-quality NMAs directly comparing and ranking anthocyanins and flavanols within the same framework (20, 22). In addition, differences in outcome timing across studies make it difficult to determine whether these supplements have distinct advantages at different stages of recovery (4, 20). Evidence on functional outcomes also remains insufficient, and it is still unclear which class is more effective for key indicators such as MVIC, CMJ, and DOMS (3, 4). Therefore, this study will conduct a systematic review and network meta-analysis to compare the relative effects of plant-derived anthocyanin and flavanol supplements on recovery after EIMD. The primary outcomes will be DOMS, muscle strength, and changes in CK and CRP at 48 h post-exercise, while the corresponding outcomes at 24 h and 72 h will be treated as secondary outcomes. This study compares the effects of different plant-derived polyphenol supplements across recovery outcomes and time points and assesses whether the findings may inform their selective use in sports nutrition practice.

## Methods

This review protocol was prospectively registered in PROSPERO (CRD420261340555) and was conducted and reported in accordance with the PRISMA 2020 statement and the PRISMA extension for network meta-analysis (PRISMA-NMA) (23, 24).

### Search strategy

We systematically searched PubMed, Web of Science, the Cochrane Central Register of Controlled Trials (CENTRAL), and Embase from database inception to January 2026, using subject headings and free-text terms related to plant-derived anthocyanin and flavanol supplements, exercise-induced muscle damage, and recovery outcomes. Two reviewers (K.-X. X. and J. C.) independently screened titles, abstracts, and full texts for eligibility. Discrepancies during study selection were resolved through discussion, and consultation with a third reviewer (Y.-J. W.) was used when consensus could not be reached. To ensure comprehensive coverage, we also searched ACSM and ECSS conference abstracts, ClinicalTrials.gov, and the reference lists of included studies and relevant reviews to identify any additional eligible studies. The complete search strategy is presented in Appendix 1.

### Eligibility criteria

Eligible studies were peer-reviewed randomized controlled trials published in English. Participants were healthy adults aged 18–60 years who underwent an acute EIMD protocol. The interventions of interest were plant-derived supplements in which anthocyanins or flavanols were the main active compounds. Eligible comparators included matched placebo conditions or vehicle-matched control conditions designed to isolate the effects of the anthocyanin- or flavanol-rich supplement. Matching could include one or more of the following domains: energy content, macronutrient composition, color, flavor, appearance, volume, capsule form, or delivery vehicle. Control conditions were eligible if they did not contain the target anthocyanin- or flavanol-rich active ingredient. Trials were not excluded solely because the comparator was not fully isoenergetic or macronutrient-matched; instead, comparator characteristics were extracted, classified, and examined in sensitivity analyses. The primary outcomes were DOMS, muscle strength, CK, and CRP at 48 h after exercise. The 48 h time point was selected for the primary analysis because it was the most consistently reported across the included studies and is practically relevant for assessing delayed soreness and early recovery after EIMD. When sufficient data were available, the corresponding outcomes at 24 h and 72 h after exercise were analyzed as secondary outcomes. Both parallel and crossover designs were eligible, provided that crossover trials included a washout period of at least 2 weeks. We excluded non-randomized trials, observational studies, animal and *in vitro* studies, conference abstracts without full-text publications, non-English publications, and studies without accessible full texts. Studies using multi-ingredient supplements were also excluded if the specific effects of anthocyanins or flavanols could not be isolated.

### Screening process

All records identified through the search were imported into EndNote X9.3.3 (Clarivate Analytics, Philadelphia, PA, USA) for management, and duplicates were removed using a combination of automated detection and manual checking. Study selection was conducted in three stages. First, two reviewers (K.-X. X. and J. C.) independently screened titles, and any record considered potentially relevant or insufficiently clear was carried forward to the next stage. Second, the abstracts of retained records were reviewed, and any disagreements were resolved through discussion or, when necessary, adjudication by a third reviewer (Y.-J. W.). Finally, the full texts of potentially eligible studies were retrieved and assessed against the predefined inclusion and exclusion criteria. No unresolved disagreements remained after discussion and, where required, consultation with the third reviewer.

### Data extraction

Before the review commenced, a standardized data extraction form was developed with the full list of extracted variables provided in Appendix 2. Study characteristics included the first author, publication year, country or region, study design, sample size, and, for crossover trials, the treatment period. Participant characteristics included sex, age, training status, exercise level, and other baseline information. EIMD protocols were recorded by exercise type, intensity or load, target muscle groups, duration, and exercise volume. For intervention and comparator, we extracted the plant source, intervention category, polyphenol class, main active compounds, product form, dose, timing, and duration of supplementation. Comparator details included the control type, such as a matched placebo or control conditions, its composition, matching features, and any reported blinding procedures. Outcome data included DOMS, strength-related outcomes, CK, and CRP, together with measurement methods, assessment time points, analytical sample sizes, effect directions, any required reverse coding, and available group-level statistics. When available, baseline and post-exercise means and standard deviations were extracted, with emphasis on 24, 48, and 72 h after EIMD. When outcome data were reported only graphically, means and error-bar values were extracted using Engauge Digitizer. For DOMS-related outcomes, effect directions were harmonized before standardized mean difference (SMD) calculation so that negative values consistently indicated a favorable intervention effect; measures with the opposite direction, such as pressure pain threshold, were reverse-coded where necessary before pooling. For strength-related outcomes, direct strength or torque measures were prioritized, including MVIC and MVC, as well as isometric or isokinetic peak torque; isokinetic total work was extracted only when these measures were unavailable and was examined in sensitivity analyses. For crossover trials, only first-period data before treatment crossover were extracted and analyzed as parallel-group comparisons. During data extraction, a small number of discrepancies were identified between the two reviewers (K.-X. X. and J. C.), mainly involving outcome data, follow-up time-point classification, and study characteristics reported inconsistently across publications. These discrepancies were resolved through discussion and consultation of the original study reports, with a third reviewer (Y.-J. W.) involved when necessary to reach consensus. All issues were resolved, and the final dataset was agreed upon by all reviewers.

### Quality assessment of evidence

The methodological quality of the included randomized controlled trials was assessed using the Cochrane Risk of Bias tool for randomized trials (ROB 2.0). The assessment covered five domains: the randomization process, deviations from intended interventions, missing outcome data, outcome measurement, and selection of the reported result (25). In accordance with the ROB 2.0 criteria, each study was judged as having low risk of bias, some concerns, or high risk of bias. The assessment was conducted independently by two reviewers (K.-X. X. and J. C.), and any disagreements were resolved through discussion or, when necessary, by consultation with a third reviewer (Y.-J. W.). Agreement between the two reviewers was good, and all discrepancies were resolved through consensus, with no unresolved issues remaining. Contour-enhanced funnel plots were generated on the basis of direct pairwise comparisons, and Egger's regression test was used to examine small-study effects and potential publication bias (26). The certainty of evidence in the treatment network was further evaluated using the CINeMA framework, which considers six domains: within-study bias, reporting bias, indirectness, imprecision, heterogeneity, and incoherence (27, 28). We assessed the comparability of treatment comparisons by examining key factors, including age, training status, supplementation timing, EIMD protocol characteristics, and the composition and matching of control conditions across studies.

### Statistical analysis

Network meta-analysis was conducted in Stata 19.5 using a frequentist approach and the mvmeta command to fit multivariate random-effects models (29, 30). Continuous outcomes were reported as mean differences (MDs) or standardized mean differences (SMDs) with 95% confidence intervals (CIs), depending on whether studies used the same measurement scale. Random-effects models were estimated using restricted maximum likelihood (REML) to account for variation between studies (31). When standard deviations for change scores were not reported directly, they were calculated, where possible, from the statistics available in the original articles using established conversion formulas (32, 33). No standard deviations were imputed using data from other studies.

Heterogeneity between studies was assessed using τ^2^ and classified as low (< 0.04), low to moderate (0.04–0.16), moderate to high (0.16–0.36), or high (>0.36) (34). A common heterogeneity variance was assumed across all comparisons, and the correlation coefficient in the between-study covariance matrix was set at 0.5 (35, 36). Because most treatment networks did not include closed loops, formal inconsistency tests could not be performed for most outcomes. Where the data and network structure allowed, we conducted exploratory checks as a descriptive assessment. Therefore, the main analyses used a consistency model, and between-study heterogeneity was assessed using τ^2^.

Network plots were used to illustrate the structure of the available evidence. Surface under the cumulative ranking curve (SUCRA) values were calculated to rank the relative effectiveness of plant-derived supplements, with higher values indicating a greater likelihood of being among the most effective interventions (37). In addition to the network meta-analysis, conventional pairwise random-effects meta-analyses and forest plots were also produced for the main outcomes to present direct treatment effects and corresponding 95% CIs. Sensitivity analyses for the primary 48-h outcomes excluded studies using acute supplementation protocols, pre-exercise-only supplementation, or post-exercise-only supplementation. Comparator-based exclusions were also applied according to the predefined categories specified in Appendix 4. For strength-related outcomes, additional sensitivity analyses excluded isokinetic total work measures and restricted analyses to isometric strength outcomes. Subgroup analyses comparing trained participants with untrained or recreationally active participants were conducted to explore potential sources of heterogeneity.

## Results

### Literature selection

**Figure 1:**
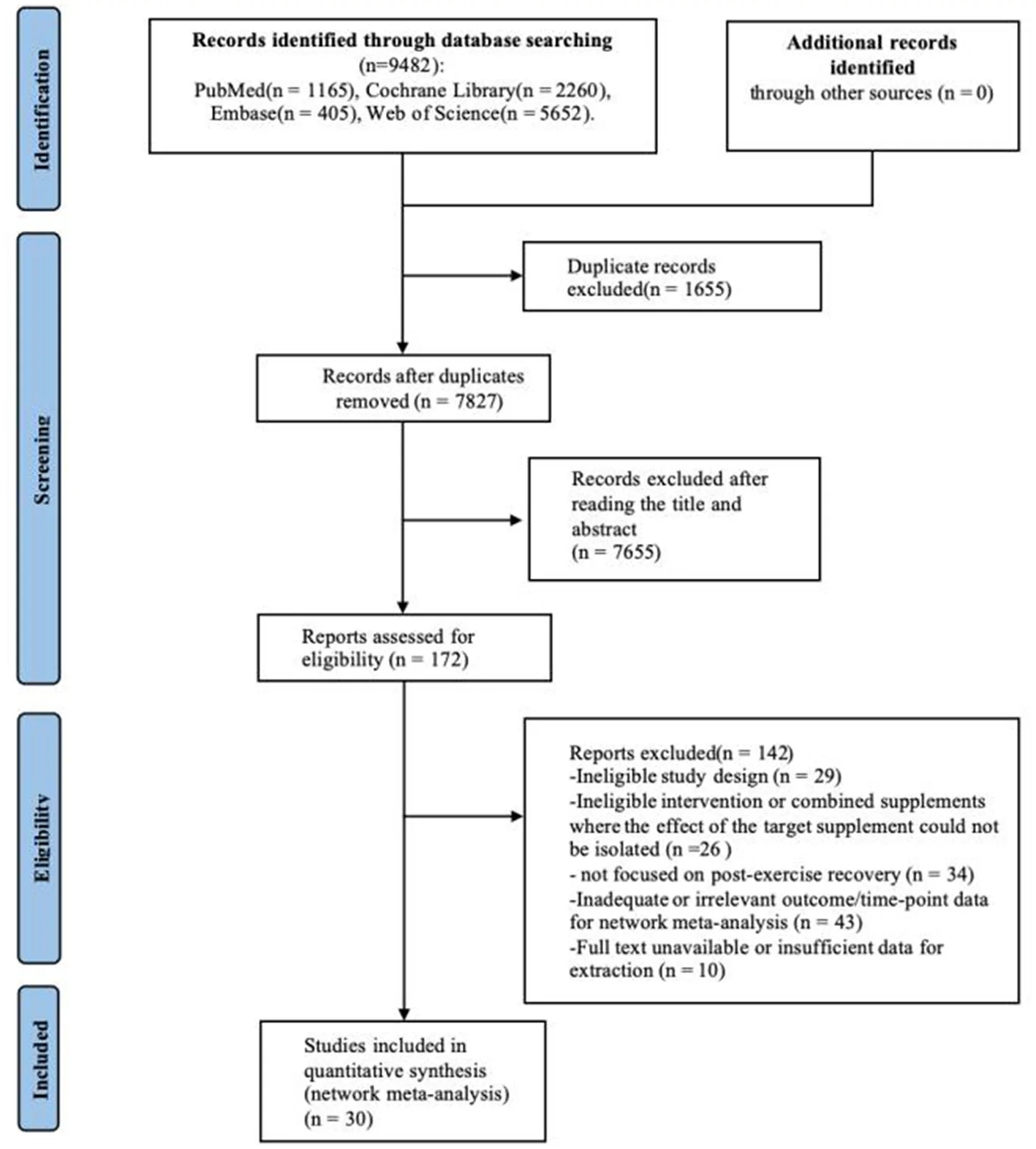
PRISMA flow diagram of study identification and selection process.

A total of 9,482 records were identified through the database search, including 1,165 from PubMed, 2,260 from the Cochrane Library, 405 from Embase, and 5,652 from Web of Science. No additional studies were identified from other sources. After duplicates were removed, 7,827 records remained for title and abstract screening, and 7,655 were excluded at this stage. The full texts of 172 articles were then reviewed for eligibility. Of these, 142 were excluded for the following reasons: ineligible study design (*n* = 29); ineligible interventions or combined supplements that did not allow the effects of the target supplement to be assessed separately (*n* = 26); lack of focus on post-exercise recovery (*n* = 34); insufficient outcome or time-point data for inclusion in the network meta-analysis (*n* = 43); and unavailable full text or insufficient data for extraction (*n* = 10). In total, 30 studies (11, 38–66) were included in the quantitative synthesis (network meta-analysis; Figure 1).

### Study characteristics

A total of 30 randomized controlled trials published between 2010 and 2026 were included, comprising 595 participants. The studies were conducted in seven countries, most commonly the United States (*n* = 14) and the United Kingdom (*n* = 10), with two studies from Poland and one each from Tunisia, Australia, New Zealand, and Spain. Sample sizes ranged from 9 to 49 participants. Across studies, participants were mainly young men, with mean ages ranging from 18.0 to 44.0 years, and included both trained and untrained individuals. EIMD was induced using a range of protocols, including traditional resistance exercise, isolated eccentric or jump-based exercise, endurance or downhill running, and sport-specific simulation tasks.

Eight plant-derived supplement nodes were investigated (Appendix 3). Tart cherry was the most frequently studied intervention (*n* = 13), followed by tea extracts (*n* = 5) and pomegranate (*n* = 4). Blackcurrant, blueberry, and cocoa flavanols were each examined in two studies, whereas grape and mixed polyphenols were each assessed in one study. Supplements were delivered in several forms, including juices or concentrates, capsules, powders, beverages, and gels. Intervention duration ranged from 1 to 45 days, although most studies used short-term supplementation during the peri-exercise period. All studies compared the active intervention with a placebo or matched control, with comparator characteristics summarized in Appendix 4. The most commonly reported outcomes were DOMS, CK, recovery of muscle strength, and inflammatory markers such as CRP or hs-CRP (Table 1).

**Table 1:** Baseline of characteristics of included studies.

Reference	Country	Design	Randomized treatments	Age (mean ± SD, yrs)	Sex (M/F)	Training status	EIMD protocol	Supplement (node)	Active component and reported amount	Dose and form	Timing and duration	Comparator	Outcomes and timepoints
Ammar et al. (38)	Tunisia	RCT	*n* = 9 crossover	21.0 ± 0.5	9M/0F	Trained	Traditional resistance training (Olympic weightlifting: snatch, clean and jerk, squat; 85–90% 1RM)	Pomegranate	Pomegranate polyphenols; per 500 ml: total polyphenols 2.56 g, o-diphenols 1.08 g, flavonoids 292.59 mg, flavonols 46.75 mg; anthocyanin/flavanol amount NR	250 ml × 3 + 500 ml once, juice	Acute; pre-only; 2 days	Flavored placebo beverage	DOMS(VAS); CK; CRP
Arent et al. (39)	USA	RCT	*n* = 18 crossover	21.3 ± 0.4	18M/0F	Trained	Endurance and anaerobic cycling (30-s Wingate + 8 × 10-s sprints; 0.10 kP/kg)	Tea extracts	Tea polyphenols; theaflavins 350 mg and catechins ~30% per 2-capsule dose	440 mg twice/day (880 mg/day), capsules	Chronic; peri-exercise; 9 days	Matched placebo capsules	DOMS (VAS)
Bell et al. (40)	UK	RCT	*n* = 8 (Exp)	30 ± 8	16M/0F	Trained	Endurance and downhill running (simulated road cycling race, 109 min; maximal sprint effort)	Tart cherry	Tart cherry polyphenols; anthocyanins 276 mg per 30 ml dose; flavanol amount NR	30 ml twice/day, concentrate juice	Chronic; peri-exercise; 8 days	Matched placebo beverage	DOMS (VAS); strength (MVIC); CK; CRP
			*n* = 8 (Con)										
Bell et al. (41)	UK	RCT	*n* = 8 (Exp) *n* = 8 (Con)	25 ± 4	16M/0F	Trained	Endurance and downhill running (Modified LIST soccer simulation: 12 × 20-m sprints + 90-min intermittent run)	Tart cherry	Tart cherry polyphenols; anthocyanins 73.5 mg/L and total phenols 178.8 mg GAE/L; flavanol amount NR	30 ml twice/day, concentrate juice	Chronic; peri-exercise; 8 days	Matched placebo beverage	DOMS (VAS); CK; CRP
Beyer et al. (42)	USA	RCT	*n* = 14 (Exp)	Exp: 21.9 ± 2.5	29M/0F	Untrained	Traditional resistance training (Squat 6 × 10 + leg press 4 × 10 + leg extension 4 × 10; 70% 1RM)	Tea extracts	Tea polyphenols; per 1,000 mg dose: total polyphenols ≥400 mg, EGCG 50–80 mg, and theaflavins 13 mg; anthocyanin amount NR	1,000mg twice/day, extract capsules	Chronic; peri-exercise; >32 days	Matched placebo capsules	DOMS (VAS); CK
			*n* = 15 (Con)	Con: 21.5 ± 2.3									
Bowtell et al. (43)	UK	RCT	*n* = 10 crossover	27.8 ± 1.6	10M/0F	Trained	Traditional resistance training (Single-leg knee extension, 10 × 10 reps; 80% 1RM)	Tart cherry	Tart cherry polyphenols; anthocyanins approximately 273.5 mg per 30 ml dose; flavanol amount NR	30 ml twice/day, concentrate juice	Chronic; peri-exercise; 10 days	Matched placebo concentrate	DOMS (PPT); strength (MVC); CK
Brown et al. (44)	UK	RCT	*n* = 10 (Exp)	19 ± 1	0M/20F	Untrained	Endurance and downhill running (15 × 30-m maximal sprints with 10-m deceleration; 60-s rest)	Tart cherry	Tart cherry polyphenols; anthocyanins 2.205 mg per 30 ml dose and total phenols 5.364 mg GAE per dose; flavanol amount NR	30 ml twice/day + 100 ml water, concentrate	Chronic; peri-exercise; 8 days	Matched placebo beverage	DOMS (PPT); CK; CRP
			*n* = 10 (Con)										
Costello et al. (45)	UK	RCT	*n* = 10 (Exp)	Exp: 30 ± 4	12M/8F	Untrained	Endurance and downhill running (Half-marathon, hilly course; best-effort pace)	Blackcurrant	Blackcurrant anthocyanins; 210 mg/day; flavanol amount NR	600 mg/day, capsules	Chronic; peri-exercise; 10 days	Matched placebo capsules	DOMS (VAS)
			*n* = 10 (Con)	Con: 29 ± 7									
de Carvalho et al. (46)	USA	RCT	*n* = 13	20.69 ± 1.49	13M/0F	Trained	Pure eccentric exercise and jumping (100 drop jumps from 60 cm; 5 × 20 reps; maximal effort)	Cocoa flavanols	Cocoa flavanols; 308 mg per dose, 616 mg/day; anthocyanin amount NR	308 mg twice/day, beverage + CHO-PRO milk	Chronic; peri-exercise; 7 days	Matched CHO-PRO milk beverage; 0 mg flavanols	DOMS (VAS); strength (MVIC); CK
Hillman et al. (47)	USA	RCT	*n* = 8 (Exp)	23 ± 5	10M/6F	Untrained	Pure eccentric exercise and jumping (plyometric drop-jump from 60 cm; 5 × 20 reps; maximal effort)	Tart cherry	Tart cherry polyphenols; product equivalent to 45 cherries per 240 ml bottle; anthocyanin/flavanol amount NR	240 ml twice/day, juice	Chronic; peri-exercise; 10 days	Matched placebo beverage	DOMS (VAS); CK
			*n* = 8 (Con)										
Hutchison et al. (48)	USA	RCT	*n* = 8 (Exp)	Exp: 19.5 ± 0.3	Exp: 1M/7F	Untrained	Traditional resistance training (eccentric knee extension, 3 × 10 reps; 115% 1RM)	Blackcurrant	Blackcurrant anthocyanins; per 100 g: malvidin glucosides 193.25 mg, cyanidin glucosides 175.69 mg; flavanol amount NR	~473 ml twice/day, juice	Chronic; peri-exercise; 8 days	Matched placebo beverage	DOMS (VAS); CK
			*n* = 8 (Con)	Con: 20.9 ± 0.9	Con: 2M/6F								
Jajtner et al. (49)	USA	RCT	*n* = 13 (PPB)	PPB: 21.8 ± 2.5	38M/0F	Untrained	Traditional resistance training (squat 6 × 10 + leg press 4 × 10 + leg extension 4 × 10; 70% 1RM)	Tea extracts	Tea polyphenols; per 1,000 mg dose: total polyphenols ≥400 mg, EGCG 50–80 mg, and theaflavins 13 mg; anthocyanin amount NR	1,000mg twice/day, extract capsules	Chronic; pre-only; 32+ days	Matched placebo capsules	CK
			*n* = 15 (PL)	PL: 21.6 ± 2.5									
			*n* = 10 (CON)	CON: 23.3 ± 4.3									
Jo et al. (50)	USA	RCT	*n* = 10 (Exp)	Exp: 22.3 ± 3.0	10M/12F	Trained	Pure eccentric exercise and jumping (vertical depth jumps 5 × 20 + leg press 2 × 6; 90% 1RM eccentric)	Mixed polyphenols	Mixed polyphenols, including trans-resveratrol, catechins, anthocyanins, flavonols, and quercetin; trans-resveratrol 60 mg/day; specific anthocyanin/flavanol amounts NR	716 mg extract + 60 mg trans-resveratrol/day, capsules	Chronic; pre-only; 30 days	Matched placebo capsules	DOMS (VAS); CK; CRP
			*n* = 12 (Con)	Con: 23.4 ± 2.7									
Jówko et al. (51)	Poland	RCT	*n* = 8 (Exp)	Exp: 22.4 ± 3.4	16M/0F	Trained	Traditional resistance training (Muscle endurance: 3 × bench press + 3 × squat to failure; 60% 1RM)	Tea extracts	Green tea catechins (including EGCG); total polyphenols 320 mg including ~250 mg catechins (~176 mg EGCG) per capsule; anthocyanin amount NR	640 mg/serving, capsule	Acute; pre-only; 1 day	Matched placebo capsules	CK
			*n* = 8 (Con)	Con: 22.9 ± 5.5									
Jówko et al. (52)	Poland	RCT	*n* = 16 crossover	21.6 ± 1.5	16M/0F	Trained	Endurance and downhill running (4 × 15-s maximal cycling sprints; 75 g/kg BW; 1-min rest)	Tea extracts	Green tea catechins (including EGCG); 245 mg polyphenols (200 mg catechins, 137 mg EGCG) per capsule; anthocyanin amount NR	2 capsules twice/day (980 mg polyphenols/day), capsules	Chronic; peri-exercise; 28 days	Matched placebo capsules	CK
Kupusarevic et al. (53)	UK	RCT	*n* = 10 crossover	28 ± 4	10M/0F	Trained	Sport-specific simulation (Professional rugby union match; 80 min; ~6,845 m total distance)	Tart cherry	Tart cherry polyphenols; equivalent to 100 sour cherries per serving; anthocyanin/flavanol amount NR	30 ml twice/day, gel	Acute; peri-exercise; 5 days	Matched placebo gel	DOMS (VAS)
Levers et al. (54)	USA	RCT	*n* = 11 (Exp)	Exp: 21.18 ± 3.34	23M/0F	Trained	Traditional resistance training (3 × 20 eccentric elbow flexions + 6 × 10 eccentric knee extensions; 110% 1RM)	Tart cherry	Tart cherry polyphenols; ~66 mg/day anthocyanins (40 mg per 290 mg powder); flavanol amount NR	480 mg/day, freeze-dried powder capsules	Chronic; peri-exercise; 10 days	Matched placebo capsules	DOMS (GPRS); strength (IK Total Ext Work); CK
			*n* = 12 (Con)	Con: 20.58 ± 1.78									
Levers et al. (55)	USA	RCT	*n* = 11 (Exp)	Exp: 20.82 ± 1.89	18M/9F	Trained	Endurance and downhill running (Half-marathon, 21.1 km; best-effort pace)	Tart cherry	Tart cherry polyphenols; ~66 mg/day anthocyanins; flavanol amount NR	480 mg/day, freeze-dried powder capsules	Chronic; peri-exercise; 10 days	Matched placebo capsules	DOMS (GPRS ); CK
			*n* = 16 (Con)	Con: 22.44 ± 4.86									
Trombold et al. (56)	USA	RCT	*n* = 17 crossover	21.9 ± 2.4	17M/0F	Trained	Traditional resistance training (Unilateral eccentric exercise: elbow flexors, 3 × 20 maximal voluntary eccentric contractions at 0.7 rad/s; knee extensors, 6 × 10 eccentric contractions at 110% 1RM; 3-min rest between sets.)	Pomegranate	Pomegranate ellagitannins; anthocyanins 384 mg/L; flavanol amount NR	250 ml twice/day, juice	Chronic; peri-exercise; 15 days	Matched placebo beverage	DOMS (VAS); strength (MVIC);
McCormick et al. (57)	Australia	RCT	*n* = 9 crossover	18.6 ± 1.4	9M/0F	Trained	Sport-specific simulation (Simulated water polo match: 8 × 5-min quarters + strength training; maximal effort)	Tart cherry	Tart cherry polyphenols; anthocyanins 9.117 mg/ml; flavanol amount NR	90 ml/day, concentrate (split doses)	Chronic; post-only; 6 days	Matched placebo beverage	DOMS (VAS); hs-CRP
McLeay et al. (58)	New Zealand	RCT	*n* = 10 crossover	22 ± 1	0M/10F	Trained	Traditional resistance training (300 maximal eccentric knee extensions; isokinetic at 30°/s)	Blueberry	Blueberry polyphenols; anthocyanins 96.6 mg/100 ml, flavonoids 10.2 mg/100 ml	~450 ml/serving, smoothie	Acute; peri-exercise; 3 days (5 total doses)	Matched placebo smoothie	DOMS (VAS); strength (IPT);
Morehen et al. (59)	UK	RCT	*n* = 11 crossover	18 ± 1	11M/0F	Trained	Sport-specific simulation (Professional rugby league match; avg 67 min; ~6,334 m; ~28 collisions)	Tart cherry	Tart cherry polyphenols; anthocyanins 320 mg per 30 ml dose; flavanol amount NR	30 ml/day + 100 ml water, concentrate	Chronic; peri-exercise; 7 days	Matched placebo beverage	DOMS (Likert Scale)
Morgan et al. (60)	UK	RCT	*n* = 10 crossover	22.8 ± 3.3	10M/0F	Untrained	Traditional resistance training (10 × 10 single-leg knee extensions; 80% 1RM; 3-s eccentric)	Cocoa flavanols	Cocoa polyphenols; per 330 ml/day: total polyphenols 154 mg, including flavanols 23 mg, catechin 43 mg, epicatechin 8 mg, and procyanidins 12 mg; anthocyanin amount NR	330 ml/day, drink	Chronic; peri-exercise; 10 days	Matched placebo beverage	DOMS (VAS); strength (MVIC); CK; CRP
O'Connor et al. (61)	US A	R CT	*n* = 20 (Exp)	Exp: 19.8 ± 1.7	Exp: 11M/9 F	Un trained	Traditional resistance training (eccentric elbow flexion, 3 × 6 reps; 120% 1RM)	Grape	Grape polyphenols; reported per kg powder: resveratrol 1.75 mg/kg, catechin 19.7 mg/kg, and quercetin 32.6 mg/kg; anthocyanin amount NR	46 g/day, freeze-dried powder in water	Chronic; peri-exercise; 45 days	Matched placebo powder	DOMS (VAS); strength (MVIC)
Torregrosa-García et al. (62)	Spain	RCT	*n* = 26 crossover	34.9 ± 10.0	26M/0F	Trained	Pure eccentric exercise and jumping (Single-leg step-down eccentric exercise; 6 × 15 reps/leg; bodyweight; 1-s concentric/4-s eccentric)	Pomegranate	Pomegranate polyphenols; 225 mg punicalagins/day; anthocyanin/flavanol amount NR	375 mg twice/day, extract capsules	Chronic; peri-exercise; 15 days	Matched placebo capsules	Strength (IPK); CK; CRP
Trombold et al. (63)	USA	RCT	*n* = 16 crossover	24.2 ± 1.4	16M/0F	Untrained	Pure eccentric exercise and jumping (2 × 20 maximal eccentric elbow flexions; Biodex isokinetic at 0.7 rad/s)	Pomegranate	Pomegranate ellagitannins; total polyphenols 650 mg containing 1% anthocyanins (~13 mg/day); flavanol amount NR	236 ml twice/day (2 bottles/day), extract drink	Chronic; peri-exercise; 9 days	Matched placebo beverage	DOMS (VAS); strength (MVIC); CK; CRP
Nieman et al. (11)	USA	RCT	*n* = 23 (Exp)	Exp: 38.8 ± 1.3	Exp: 12M/11F	Untrained	Pure eccentric exercise and jumping (90-min full-body eccentric protocol; 17 exercises; 2–3 sets; 30–60-s rest)	Blueberry	Blueberry polyphenols; anthocyanins 280 mg/day, total phenols 805 mg/day; flavanol amount NR	25 g/day, freeze-dried powder	Chronic; peri-exercise; 18 days	Matched placebo powder	DOMS (VAS); CK
			*n* = 26 (Con)	Con: 39.0 ± 1.3	Con: 12M/14F								
Quinlan et al. (64)	UK	RCT	*n* = 10 (Exp)	Exp: 28 ± 4	Exp: 4M/6F	Trained	Sport-specific simulation (modified LIST: 6 × 15-min intermittent running + 12 × 20-m maximal sprints)	Tart cherry	Tart cherry polyphenols; anthocyanin/flavanol amount NR	30 ml/day + 70 ml water, concentrate	Chronic; peri-exercise; 8 days	Matched placebo beverage	DOMS (VAS); strength (MVIC); CK; CRP
			*n* = 10 (Con)	Con: 25 ± 5	Con: 4M/6F								
Ortega et al. (65)	USA	RCT	*n* = 17 crossover	22.2 ± 3.3	0M/17F	Untrained	Pure eccentric exercise and jumping (8 × 10 maximal concentric/eccentric knee extensions; isokinetic at 60°/s)	Tart cherry	Tart cherry polyphenols; anthocyanin/flavanol amount NR	1,000 mg/day, concentrated extract capsules	Chronic; peri-exercise; 8 days	Matched placebo capsules	DOMS (VAS); strength (IPK 60°/s)
Squires et al. (66)	UK	RCT	*n* = 17 (Exp)	Exp: 36 ± 9	Exp: 11M/6F	Untrained	Endurance and downhill running (trail marathon, 42.195 km; 613 m elevation gain; full effort)	Tart cherry	Tart cherry polyphenols; anthocyanins 68.4 mg/day (pre) and 123.1 mg/day (post-marathon); flavanol amount NR	2.2 g/day pre + 3.96 g/day post, spray-dried extract capsules	Chronic; peri-exercise; 7 days	Matched placebo capsules	DOMS (VAS); CK; hs-CRP
			*n* = 18 (Con)	Con: 44 ± 9	Con: 12M/6F								

RCT, randomized controlled trial; NR, not reported in the original study; GAE, gallic acid equivalents; Exp, experimental group; Con, control group; M, male; F, female; yrs, years; BW, body weight; 1RM, one-repetition maximum; EIMD, exercise-induced muscle damage; LIST, Loughborough Intermittent Shuttle Test; DOMS, delayed onset muscle soreness; VAS, visual analog scale; PPT, pressure pain threshold; MVIC, maximal voluntary isometric contraction; MVC, maximal voluntary contraction; CMJ, countermovement jump; IPT, isometric peak torque; IPK, isokinetic peak torque; IK Total Ext Work, isokinetic total extension work; CK, creatine kinase; CRP, C-reactive protein; hs-CRP, high-sensitivity C-reactive protein; GPRS, general pain rating scale; CHO-PRO, carbohydrate-protein; EGCG, epigallocatechin gallate.

### Risk of bias, certainty of evidence, and consistency

The risk-of-bias assessment is presented in Appendix 5. Overall, the included studies were methodologically sound, with most judged to be at low risk of bias across the assessed domains. Low-risk ratings were assigned to 29 studies (96.67%) for both the randomization process and selective reporting, 28 (93.33%) for outcome measurement, 26 (86.67%) for missing outcome data, and 24 (80.00%) for deviations from intended interventions. This last domain showed a relatively higher proportion of studies with some concerns, suggesting that the delivery and monitoring of interventions were not always fully described. In total, 20 studies (66.67%) were judged to be at low risk of bias, 8 (26.67%) raised some concerns, and 2 (6.67%) were considered at high risk. Most outcome networks did not include closed loops, so formal inconsistency tests could not be performed in most cases. Exploratory checks were conducted only for the few networks where the structure allowed, and these did not show clear evidence of inconsistency. However, because most networks could not be formally assessed, these findings should be interpreted with caution. In addition, τ^2^ values were generally low, suggesting limited between-study heterogeneity. Although slightly higher values were observed for DOMS at 48 h and 72 h, muscle strength at 48 h, and CK at 72 h, these remained within a low-to-moderate range. Overall, the network meta-analysis findings appeared stable (Appendix 6). The CINeMA assessment showed that confidence in the evidence was generally low across comparisons. Most estimates for 48 h DOMS, muscle strength, CK, and CRP were rated as low confidence, mainly because of imprecision, sparse direct evidence, and the limited ability to fully assess inconsistency in networks without closed loops. No major concerns regarding transitivity were identified after considering participant characteristics, EIMD protocols, supplementation features, and comparator characteristics (Appendix 11). Funnel plots and Egger's tests showed no clear asymmetry for muscle strength and CRP, whereas some asymmetry was observed for DOMS and CK at certain time points, suggesting that these results should be interpreted cautiously (Appendix 12).

### Primary network analysis

#### 48 -h DOMS

**Figure 2:**
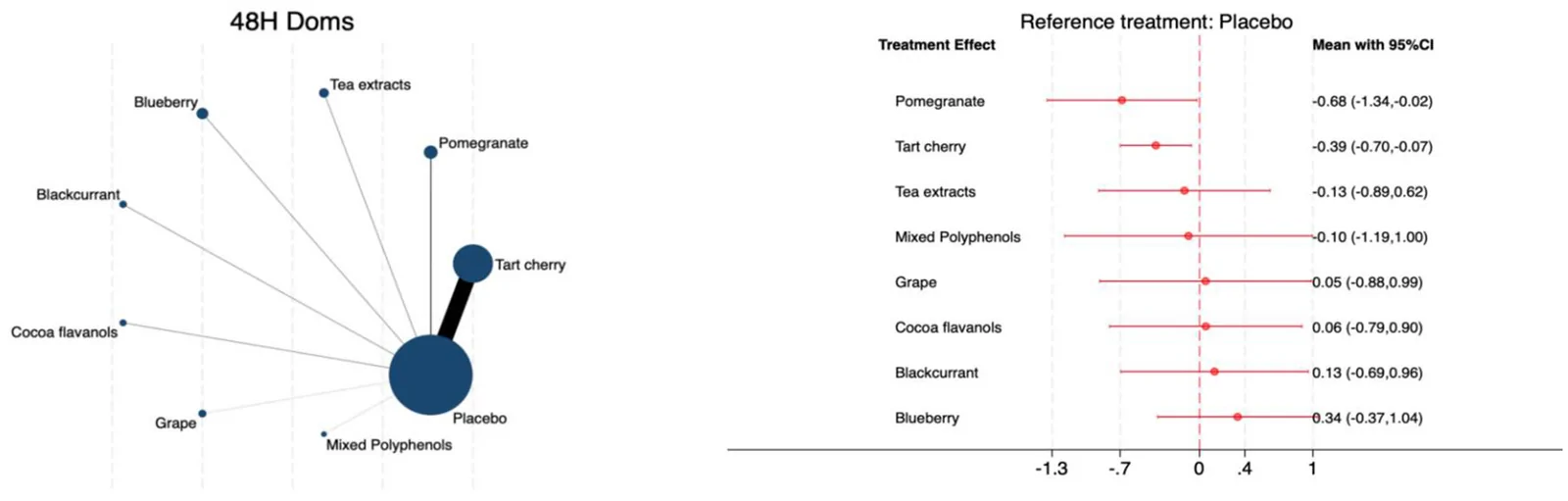
Network and forest plots of 48-h DOMS. Node size is proportional to participant number, and edge thickness to the number of studies for each comparison. The placebo node pools all eligible control conditions, including matched placebo and vehicle-matched controls. Negative SMD values favor plant-derived supplements.

For DOMS at 48 h post-exercise, 26 trials involving 499 participants and 9 nodes were included. All eligible control conditions, including matched placebo and vehicle-matched control conditions, were grouped into a single placebo/control node for the network analysis. This node is labeled as “placebo node” in the network plots for simplicity. This node served as the main common comparator, and the network was fully connected with no isolated nodes (Figure 2). Compared with placebo node, only pomegranate and tart cherry showed statistically significant effects. Pomegranate ranked highest (*SMD* = −0.68, 95% *CI* −1.34 to −0.02; *SUCRA* = 89.3%), followed by tart cherry (*SMD* = −0.39, 95% *CI* −0.70 to −0.07; *SUCRA* = 76.0%; Figure 2; Appendix 9, Figure S9.1 and Table S9.1). No significant effects were observed for the other interventions. The league table was broadly consistent with the forest plot: in addition to being superior to placebo node, pomegranate was also significantly more effective than blueberry (*SMD* = −1.02, 95% *CI* −1.98 to −0.06), whereas no other pairwise comparisons reached statistical significance (Appendix 10, Table S10.1). Pairwise random-effects meta-analysis supported the NMA findings, showing a small but significant reduction in 48 h DOMS with plant-derived supplements vs. control conditions (*SMD* = −0.25, 95% *CI* −0.48 to −0.02; *I*^2^ = 48.0%; Appendix 7, Figure S7.1). According to the CINeMA assessment, the overall certainty of evidence for this outcome was low (Appendix 11, Table S11.2).

#### 48 -h muscle strength

**Figure 3:**
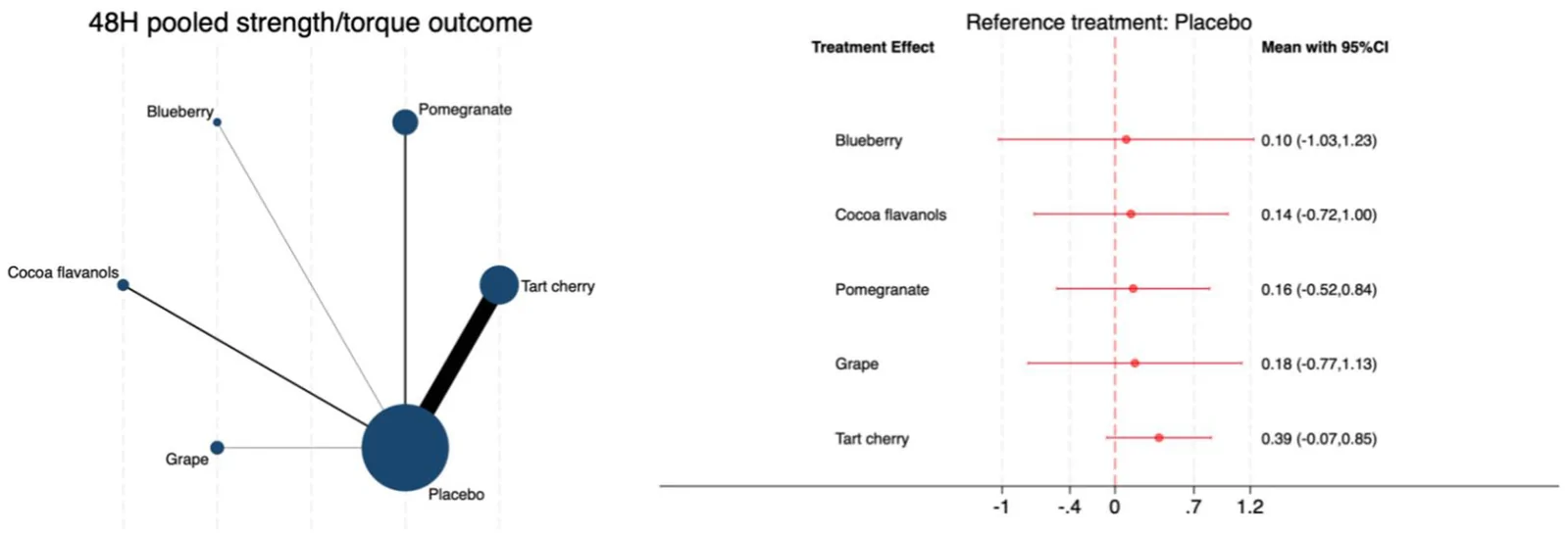
Network and forest plots for the 48-h pooled muscle strength outcome. Node size is proportional to participant number, and edge thickness to the number of studies for each comparison. The placebo node pools all eligible control conditions, including matched placebo and vehicle-matched controls. Positive SMD values favor plant-derived supplements.

At 48 h, the muscle strength outcome included 13 randomized controlled trials with 237 participants and 6 nodes. The network was well connected, with placebo node as the main common comparator. Most plant polyphenol interventions showed a trend toward better muscle strength recovery than placebo node, but the 95% confidence intervals generally included no effect (Figure 3). According to the SUCRA rankings, tart cherry ranked highest (73.3%), followed by grape (51.9%) and pomegranate (50.1%). However, none of these interventions differed significantly from placebo node (Appendix 9, Figure S9.2 and Table S9.2). The league table showed a similar pattern, with no significant differences in any pairwise network comparisons, suggesting that differences between plant polyphenol interventions in muscle strength recovery at 48 h were limited (Appendix 10, Table S10.2). A pairwise random-effects meta-analysis indicated a small, non-significant tendency for plant-derived supplements to improve strength-related outcomes at 48 h (*SMD* = 0.23, 95% *CI* −0.03 to 0.48; *I*^2^ = 23.5%; Appendix 7, Figure S7.2). The CINeMA assessment indicated that the overall certainty of evidence for this outcome was low (Appendix 11, Table S11.3).

#### 48 -h CK

**Figure 4:**
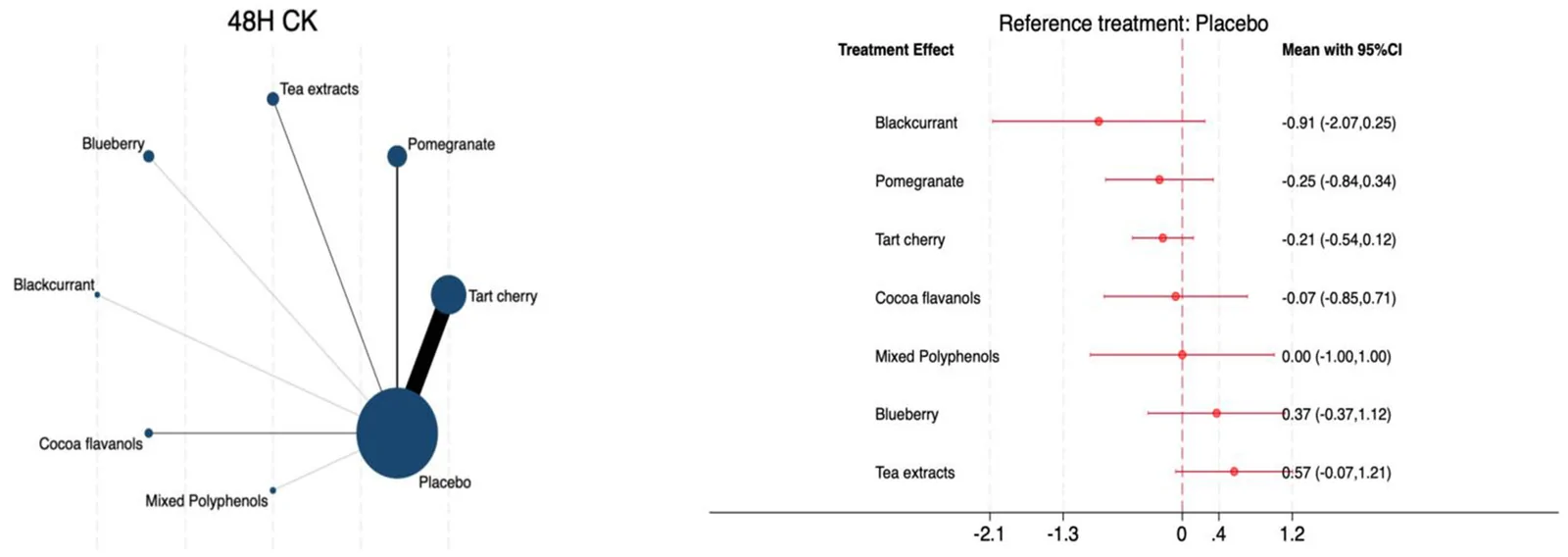
Network plot and forest plot for 48-h CK outcome. Node size is proportional to the number of participants, and edge thickness is proportional to the number of studies informing each comparison. The placebo node pools all eligible control conditions, including matched placebo and vehicle-matched controls. Negative SMD values favor plant-derived supplements.

For the 48-h CK outcome, this network meta-analysis included 19 trials with 411 participants across 8 nodes. Placebo node was the main reference treatment and linked most of the active interventions, resulting in a complete network structure. However, none of the comparisons between the active interventions and placebo node showed a statistically significant effect (Figure 4). Although the ranking analysis placed blackcurrant (*SUCRA* = 90.8%), pomegranate (*SUCRA* = 67.2%), and tart cherry (*SUCRA* = 66.5%) highest for reducing CK levels, all corresponding 95% confidence intervals crossed the null value. These findings suggest possible differences in relative ranking, but they do not provide firm evidence of superiority and should therefore be interpreted cautiously (Appendix 9, Figure S9.3; Table S9.3). In the league table, pairwise network estimates suggested lower CK values for tart cherry and blackcurrant compared with tea extracts, whereas all other pairwise network comparisons were not statistically significant. (Appendix 10, Table S10.3). A pairwise random-effects meta-analysis showed a small, non-significant reduction in CK at 48 h with plant-derived supplements vs. control (*SMD* = −0.11, 95% *CI* −0.38 to 0.15; *I*^2^ = 47.9%; Appendix 7, Figure S7.3). Overall, the certainty of the evidence for this outcome was low according to the CINeMA assessment (Appendix 11, Table S11.4).

#### 48 -h CRP

**Figure 5:**
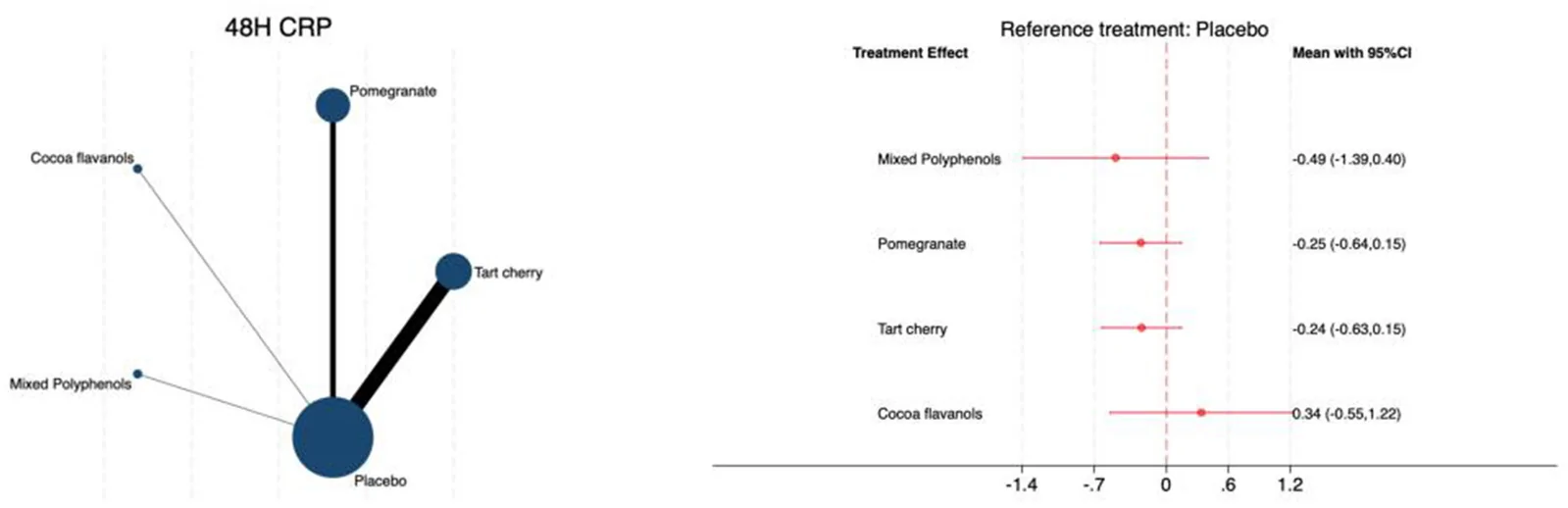
Network and forest plots for the 48-h CRP outcome. Node size is proportional to participant number, and edge thickness to the number of studies for each comparison. The placebo node pools all eligible control conditions, including matched placebo and vehicle-matched controls. Negative SMD values favor plant-derived supplements.

For the 48-h CRP outcome, this network meta-analysis included 10 studies with 190 participants across 5 nodes. Placebo node was the main reference treatment and connected all interventions within the network. No active intervention showed a statistically significant reduction in CRP compared with placebo node (Figure 5). Mixed polyphenols (*SUCRA* = 78.4%), pomegranate (*SUCRA* = 65.1%), and tart cherry (*SUCRA* = 63.9%) ranked highest for CRP reduction. However, all corresponding 95% confidence intervals crossed the null value, indicating that these rankings reflect relative probability rather than clear evidence of benefit. These findings should therefore be interpreted with caution (Appendix 9, Figure S9.4; Table S9.4). In addition, no significant differences were found in any pairwise network comparisons (Appendix 10, Table S10.4). Conventional pairwise meta-analysis likewise showed no significant effect on 48-h CRP (*SMD* = −0.22, 95% *CI* −0.48 to 0.03; *I*^2^ = 0.0%; Appendix 7, Figure S7.4), and the overall certainty of the evidence was low according to the CINeMA assessment (Appendix 11, Table S11.5).

### Secondary network analysis

#### DOMS at 24 h and 72 h

For the 24-h DOMS outcome, 25 trials involving 461 participants and 9 nodes were included. The network was centered on the placebo node and included no isolated nodes (Appendix 8, Figure S8.1). Of all interventions, only tart cherry showed a significant reduction in DOMS compared with placebo node (*SMD* = −0.34, 95% *CI* −0.58 to −0.10; *SUCRA* = 81.7%). No other intervention showed a significant effect. Tea extracts (*SUCRA* = 81.0%) and pomegranate (*SUCRA* = 69.0%) also ranked relatively high, but neither showed a significant benefit (Appendix 9, Figure S9.5; Table S9.5). Results from the league table were consistent with the forest plot. Tart cherry was superior to both placebo node and blueberry (*SMD* = −0.56, 95% *CI* −1.09 to −0.03), whereas no significant differences were found among the other interventions (Appendix 10, Table S10.5).

For the 72-h DOMS outcome, 10 trials involving 192 participants and 5 nodes were included. Placebo node was the main comparator and connected all interventions in the network, with no isolated nodes. Among all treatments, only tart cherry showed a significant reduction in DOMS compared with placebo node (*SMD* = −0.56, 95% *CI* −1.04 to −0.07; *SUCRA* = 85.3%), while no other intervention showed a significant effect (Appendix 8, Figure S8.5). Pomegranate (*SUCRA* = 63.8%) and blackcurrant (*SUCRA* = 50.5%) also ranked relatively high, but neither showed a significant benefit (Appendix 9, Figure S9.9 and Table S9.9). Results from the league table were consistent with the forest plot, showing a significant advantage only for tart cherry over placebo, with no significant differences among the other interventions (Appendix 10, Table S10.9).

#### Muscle strength at 24 h and 72 h

For the 24-h muscle strength outcome, 12 trials involving 224 participants and 6 nodes were included. The network was centered on the placebo node and included no isolated nodes. Most plant polyphenol interventions showed a tendency to improve muscle strength relative to placebo node, but none of these effects reached statistical significance (Appendix 8, Figure S8.2). Based on the ranking analysis, blueberry had the highest probability of being the most effective intervention (*SUCRA* = 79.4%), followed by tart cherry (*SUCRA* = 69.1%) and grape (*SUCRA* = 58.2%) (Appendix 9, Figure S9.6 and Table S9.6). The league table showed a similar pattern, with no significant differences between interventions, suggesting that any differences in effect were small (Appendix 10, Table S10.6).

For the 72-h muscle strength outcome, 6 trials involving 111 participants and 3 nodes were included. Although the network was simpler, it remained fully connected. Compared with the placebo node, both tart cherry and pomegranate showed a trend toward better muscle strength recovery than placebo node, but neither comparison was statistically significant (Appendix 8, Figure S8.6). The ranking analysis placed pomegranate first (*SUCRA* = 71.1%), followed by tart cherry (*SUCRA* = 68.7%) (Appendix 9, Figure S9.10 and Table S9.10). Consistent with these findings, the league table showed no significant differences between interventions, indicating little separation in their effects on muscle strength recovery at 72 h (Appendix 10, Table S10.10).

#### CK at 24 h and 72 h

For the 24-h CK outcome, 20 trials involving 330 participants and 8 nodes were included. Placebo node was the main comparator, and the network was well connected, with no isolated nodes. Overall, most plant polyphenol interventions showed a trend toward lower CK levels than placebo node, although most comparisons were not statistically significant (Appendix 8, Figure S8.3). In the ranking analysis, tart cherry ranked highest (*SUCRA* = 77.8%), followed by blackcurrant (*SUCRA* = 73.8%) and mixed polyphenols (*SUCRA* = 63.4%) (Appendix 9, Figure S9.7 and Table S9.7). However, the league table showed no significant differences between interventions, suggesting that any differences in effect at 24 h were modest (Appendix 10, Table S10.7).

For the 72-h CK outcome, 7 trials involving 159 participants and 4 nodes were included. The network was simpler but remained fully connected, with placebo node as the main comparator. None of the interventions showed a statistically significant effect compared with placebo node (Appendix 8, Figure S8.7). Tart cherry (*SUCRA* = 66.1%), placebo node (*SUCRA* = 64.3%), and pomegranate (*SUCRA* = 45.7%) ranked highest, but all corresponding 95% confidence intervals crossed the null value. The relatively high ranking of placebo further suggests that differences between interventions were small and that there was no clear evidence of superiority for any treatment (Appendix 9, Figure S9.11 and Table S9.11). This was consistent with the league table, which showed no significant differences between interventions (Appendix 10, Table S10.11).

### CRP at 24 h and 72 h

For the 24-h CRP outcome, 10 trials involving 190 participants and 5 nodes were included. Placebo node served as the main comparator, and the network was fully connected, with no isolated nodes. Overall, most plant polyphenol interventions were associated with lower CRP levels than placebo node, but none showed a statistically significant effect because all 95% confidence intervals included zero (Appendix 8, Figure S8.4). In the ranking analysis, pomegranate ranked first (*SUCRA* = 69.0%), followed by tart cherry (*SUCRA* = 63.8%) and mixed polyphenols (*SUCRA* = 56.9%) (Appendix 9, Figure S9.8 and Table S9.8). However, the league table showed no significant differences between interventions, suggesting that any differences in effect at 24 h were small (Appendix 10, Table S10.8).

For the 72-h CRP outcome, 5 trials involving 94 participants and 3 nodes were included. The network was simpler but remained fully connected. Both tart cherry and pomegranate showed a tendency to reduce CRP compared with placebo node, but neither effect was statistically significant (Appendix 8, Figure S8.8). Pomegranate ranked first in the ranking analysis (*SUCRA* = 64.8%), closely followed by tart cherry (*SUCRA* = 64.5%) (Appendix 9, Figure S9.12 and Table S9.12). The league table likewise showed no significant differences between interventions, indicating little separation in their effects on CRP reduction at 72 h (Appendix 10, Table S10.12).

### Subgroup analysis

Recovery after exercise-induced muscle damage may differ by training status. To examine this, the included studies were grouped into trained participants and untrained or recreationally active participants, and subgroup analyses were conducted for 48-h DOMS, muscle strength, CK, and CRP (Appendix 14). Overall, training status appeared to affect some outcomes. Among trained participants, plant polyphenols showed a clearer benefit for 48-h DOMS, with tart cherry and pomegranate performing significantly better than placebo. No consistent significant effects were observed among untrained or recreationally active participants. For 48-h muscle strength and CRP, no intervention showed a significant advantage in either subgroup.

The pattern for 48-h CK also varied by training status. Among trained participants, no intervention differed significantly from placebo. Among untrained or recreationally active participants, tea extracts were associated with higher CK levels than placebo, suggesting limited benefit in this group. Taken together, these findings suggest that training status may influence the effects of plant polyphenols on recovery, particularly for DOMS and CK. However, the evidence remains limited because each subgroup included few studies and the confidence intervals were wide.

### Sensitivity analyses

Sensitivity analyses tested whether the findings changed after excluding specific exercise protocols, supplementation timings, muscle strength measures, and less comparable control conditions. These controls included sensory-matched comparators with different nutritional content, partially matched or unclear comparators, nutritionally mismatched controls, and energy-containing placebos. Overall, most 48-h estimates remained in the same direction as the primary analyses, supporting the reliability of the main findings (Appendix 13, Tables S13.1–S13.5). Results for 48-h muscle strength outcomes and CRP were generally stable, and no sensitivity analysis changed the main conclusions. Some analyses could not be performed because too few studies were available or because the networks could not be estimated.

For 48-h DOMS, tart cherry showed a favorable effect across most sensitivity analyses, suggesting relatively robust findings. However, this effect became smaller and was no longer statistically significant when the strictest control-condition exclusions were applied. Pomegranate also showed a favorable effect in the primary analysis, but its results were less stable. Although the overall direction of effect remained beneficial and the effect size increased in some analyses, the effect was weakened and became non-significant after excluding partially matched or unclear comparators and sensory-matched comparators with different nutritional content. These findings suggest that the potential benefit of pomegranate for 48-h DOMS may be more affected by study design and comparator definition. Results for other interventions changed only slightly and did not affect the overall conclusions. For 48-h CK, some findings were sensitive to study design and control conditions. Tea extract was not statistically significant in the primary analysis but became significantly unfavorable after excluding studies with acute exercise protocols and partially matched or unclear controls. Pomegranate and cocoa flavanols also showed changes in effect direction in some analyses. However, these estimates were uncertain, with wide confidence intervals, and most results remained non-significant. These findings should therefore be interpreted with caution.

Taken together, the sensitivity analyses were broadly consistent with the primary analyses and supported the main conclusions. Tart cherry showed the most stable effect for improving 48-h DOMS, whereas the effects of pomegranate on DOMS and tea extract on CK appeared more dependent on study design and comparator definition. The changes observed in some CK analyses suggest that exercise protocol, supplementation timing, and comparator design may influence the estimated effects. For CRP, the significant effect of tart cherry appeared only under the strictest control-condition exclusions and should therefore be considered exploratory. Because some sensitivity analyses included only a small number of studies, these findings should be interpreted cautiously.

## Discussion

### Primary findings

The main network meta-analysis found that plant-derived polyphenol supplements did not provide consistent benefits across all outcomes after EIMD. Their potential value appeared to lie primarily in reducing DOMS, whereas evidence for improved muscle strength recovery and biochemical markers, including CK and CRP, remained limited and inconsistent. This pattern was supported by the conventional pairwise meta-analyses, which showed a small overall reduction in 48-h DOMS but no significant pooled effects for 48-h muscle strength, CK, or CRP. Among the interventions examined, tart cherry and pomegranate showed the most promising effects on pain-related recovery. Pomegranate also appeared to improve 48 h DOMS, but its effect varied more across sensitivity analyses and was therefore less certain. For tea extract, blackcurrant, blueberry, cocoa flavanols, grape, and mixed polyphenols, the effects were generally small, or the available evidence was too limited to draw firm conclusions. Sensitivity analyses did not meaningfully change the overall findings of this study. However, some results varied depending on factors such as study design, supplementation protocol, outcome measures, and comparator characteristics. Notably, the effects of pomegranate on DOMS and tea extract on CK differed across analytical approaches, indicating some uncertainty in these estimates. Taken together, the evidence suggests that the effectiveness of plant-derived polyphenol supplements depends on both the type of supplement and the recovery outcome being measured. The strongest and most consistent benefits were observed for reducing perceived muscle soreness. In contrast, evidence supporting improvements in muscle strength recovery and objective physiological markers, including CK and CRP, remains limited and inconsistent.

### Comparison with previous research

Our network meta-analysis found that plant-derived supplements did not produce consistent benefits across all recovery outcomes after exercise-induced muscle damage. Their main potential benefit appeared to be a reduction in delayed onset muscle soreness, whereas evidence for improvements in muscle strength recovery and in objective markers such as creatine kinase and C-reactive protein remained limited and inconsistent (19). These findings broadly support earlier reports that plant-derived supplements may improve the subjective experience of recovery after exercise, but they are more cautious than the broader positive conclusions reported in some previous pairwise meta-analyses (19). This difference is likely related to variations in statistical methods and in the way heterogeneity was addressed.

Previous systematic reviews and pairwise meta-analyses often combined plant polyphenol supplements from different sources into a single intervention category, despite differences in their chemical composition and biological properties (19). Although this approach can increase sample size and improve statistical power, it may also mask meaningful differences between individual supplements and produce an overall effect that mainly reflects an average across diverse interventions. As a result, earlier findings of “overall effectiveness” may describe a broad group-level signal rather than the true effect of a specific supplement on a particular recovery outcome. In contrast, to our knowledge, this is the first study to evaluate different plant-derived anthocyanin- and flavanol-rich supplements as separate nodes within a single network meta-analysis. This approach allowed us to combine direct and indirect evidence while preserving differences between interventions, providing a more precise comparison of their relative effects and reducing bias caused by grouping distinct supplements together.

Viewed in this way, the more consistent benefits for objective recovery outcomes reported in earlier studies were not confirmed when supplements were assessed separately. Instead, our analysis showed that only tart cherry and pomegranate had relatively clear advantages for subjective pain-related outcomes such as delayed onset muscle soreness, whereas evidence for other supplements and for other objective recovery outcomes remained limited. Therefore, our findings do not contradict earlier research, but rather refine and clarify it through a more rigorous comparative approach. While previous studies mainly asked whether plant polyphenols are effective overall, our study further examined which specific supplements are more likely to be beneficial and which aspects of recovery they are most likely to affect. Overall, the current evidence suggests that the effects of plant-derived supplements on recovery after exercise-induced muscle damage are more likely to be supplement-specific and outcome-specific than consistent across all supplements and all recovery measures (19).

### Potential mechanisms of tart cherry and pomegranate in alleviating DOMS

Tart cherry and pomegranate both contain anthocyanins and flavan-3-ols or proanthocyanidins, although the amounts and relative proportions of these compounds vary across raw materials and formulations (67, 68). *In vitro* studies suggest that cyanidin and certain catechin-type flavanols can inhibit COX-1 and COX-2 activity. In non-muscle animal models, cyanidin-3-O-glucoside has also been shown to reduce COX-2 expression and PGE2 production (69, 70). These findings suggest that regulation of the COX-2/PGE2 pathway may help reduce peripheral pain sensitivity after exercise, although this mechanism has not been directly confirmed in human skeletal muscle. Cell and animal studies further indicate that cyanidin-3-O-glucoside may suppress NF-κB signaling, reduce inflammatory mediators such as TNF-α and IL-6, and limit oxidative stress and lipid peroxidation (70, 71). These effects may reduce secondary membrane damage, inflammatory amplification, and pain-receptor sensitization after muscle injury, thereby supporting inflammation resolution and alleviating delayed-onset muscle soreness. This explanation is consistent with some human eccentric-exercise trials reporting reduced soreness after tart cherry or pomegranate supplementation, although these studies did not directly measure the proposed pathways (56, 72). The effectiveness of these supplements may also depend on product composition, bioactive compound exposure, dose and timing, exercise model, and training status. A direct comparison in resistance-untrained men found no significant benefit of either tart cherry or pomegranate (73). Likewise, a recent randomized, double-blind, placebo-controlled trial found that tart cherry powder did not improve muscle soreness, perceived recovery, exercise performance, or creatine kinase responses after repeated-sprint exercise (74). Together, these findings may suggest that the effects of both supplements are context dependent. Overall, the proposed mechanisms may partly explain the more consistent reduction in delayed-onset muscle soreness observed with tart cherry in the present study, whereas the effects of pomegranate appear less consistent across studies.

### Possible explanation for the gap between subjective pain and objective recovery markers

Although some plant-derived polyphenol supplements appeared to reduce DOMS, these benefits were not matched by improvements in muscle strength or in objective markers such as CK and CRP. This mismatch suggests that their effects may be limited to specific aspects of recovery rather than the broader physiological repair process. One explanation is that DOMS and CK reflect different forms of tissue response and follow different time courses. DOMS is more closely linked to microdamage in connective tissue, fascia, and musculotendinous regions, together with the secondary inflammatory response that increases pain sensitivity (75). Polyphenols and their circulating metabolites may contribute to pain attenuation by modulating COX-2 and related inflammatory pathways, particularly in well-perfused tissues involved in exercise recovery (76). CK, by contrast, is more closely associated with membrane disruption and structural damage in deeper muscle fibers. While polyphenols may lessen secondary oxidative stress, they are unlikely to reverse established membrane injury. CK release is also delayed, with peak serum levels often occurring later than peak soreness (75). This may explain why pain improves without a parallel reduction in CK. A second explanation is that pain relief does not necessarily translate into functional recovery. Loss of muscle strength after EIMD is driven in part by disruption of excitation—contraction coupling and sarcomere integrity (77). Although polyphenols may reduce inflammation and oxidative stress, they are unlikely to directly restore normal calcium handling or repair damaged contractile structures. Muscle strength may therefore remain impaired even when soreness is reduced (77, 78). In addition, both CK and CRP are highly variable measures, which makes treatment effects harder to detect. A recent literature review identified body composition and genetic variation as important contributors to post-exercise CK variability (79). CRP reflects systemic inflammation and may not closely track local exercise-induced muscle damage, as appreciable increases may occur primarily in individuals with greater muscle damage (80). Given the current sample size and evidence base, any true effects of plant polyphenols on CK and CRP may therefore be difficult to show consistently. Taken together, the available evidence suggests that plant-derived polyphenol supplements are more likely to influence subjective pain than to produce parallel improvements in muscle function or systemic biochemical markers after EIMD.

### Potential differences between supplements and implications of the subgroup and sensitivity analyses

The secondary analyses suggest that plant-derived polyphenol supplements may differ in their effects across recovery outcomes, with some agents ranking more favorably for specific measures. These rankings, however, should be interpreted cautiously. SUCRA reflects the probability that an intervention ranks higher than others, not the size or practical importance of its effect (81). In this study, the treatment network was relatively sparse, and most comparisons between active interventions and placebo had 95% confidence intervals that crossed the null value. Accordingly, these rankings are better viewed as hypothesis-generating signals than as evidence of clear superiority. Reviews have proposed that differences in phytochemical composition and metabolic behavior may contribute to supplement-specific effects on microcirculation, oxidative stress, and inflammatory pathways (21, 82). At present, however, this remains a mechanistic inference rather than a firm conclusion. The subgroup analyses further suggested that participant type may influence some recovery outcomes. At 48 h, the reduction in DOMS appeared more pronounced in trained participants, whereas results in untrained or recreationally active participants were less consistent. This pattern may reflect the stronger endogenous antioxidant capacity and greater tissue adaptation typically seen in trained populations (8, 79, 83). Sensitivity analyses were generally consistent with the main findings, especially for DOMS, where tart cherry showed the most stable beneficial effect. However, changes in some DOMS, CK, and CRP estimates after stricter exclusions suggest that study design, supplementation timing, outcome definition, and comparator characteristics may influence the results. Overall, the effects of plant-derived polyphenol supplements appear to vary by supplement, population, and recovery outcome assessed (84, 85).

### Strengths, robustness, and methodological limitations

#### Strengths and robustness

A key strength of this study is the use of a network meta-analysis, which made it possible to compare the relative effects of eight plant-derived polyphenol supplements within a single analytical framework despite the limited availability of direct head-to-head trials. Unlike earlier reviews that combined different polyphenols into a broad intervention category, this approach allowed a more precise assessment of how individual supplements may differ across recovery outcomes. Conventional pairwise meta-analyses were also conducted and broadly supported the direction of the network meta-analysis findings, although the level of agreement varied across outcomes. Robustness was further assessed through sensitivity analyses excluding studies with extreme exercise protocols, different supplementation timings, narrower definitions of strength-related outcomes, and increasingly strict control-condition criteria. These analyses were important because EIMD studies vary considerably in exercise protocols, supplementation strategies, intervention duration, outcome measures, and control conditions. Overall, the main conclusions were largely maintained across sensitivity analyses, particularly for DOMS, where tart cherry consistently showed favorable effects. Some variation in estimates was observed after stricter exclusions, indicating that study characteristics may influence the magnitude of effects.

#### Limitations

Several limitations should also be considered. First, although network meta-analysis is useful when direct comparative evidence is limited, the network in this study was relatively sparse. Most interventions were linked indirectly through a common placebo node, and direct comparisons between different plant-derived supplements were uncommon. Most networks also lacked closed loops, limiting formal inconsistency assessment using node-splitting or loop-specific methods. Where such assessments were possible, no obvious inconsistency was identified; however, inconsistency could not be comprehensively evaluated across all networks. Second, the evidence was not evenly distributed across the plant-derived supplements included in this review. Tart cherry had the strongest evidence base, while other supplements, such as blueberry, blackcurrant, cocoa flavanols, grape, and mixed polyphenols, were examined in only one or two studies. Consequently, the estimated effects and rankings of these less-studied supplements are less reliable and should be interpreted with caution. Third, the certainty of evidence was generally low, largely because studies were small and estimates were imprecise. Fourth, although statistical heterogeneity was generally low, important between-study differences remained in supplementation protocols, EIMD models, outcome measures, and comparator characteristics (19, 54). Although 48 h was selected as the primary time point because it was the most consistently reported and practically relevant assessment point across studies, it may not capture the peak response of all recovery markers. In particular, CK and CRP concentrations may peak earlier or later than 48 h depending on the EIMD protocol, participant characteristics, and individual variability in muscle damage and inflammatory responses. Differences in control-condition classification, matching features, and blinding procedures may also have influenced some effect estimates. Finally, few included studies considered individual differences in polyphenol metabolism. Human studies of anthocyanin- and flavanol-rich interventions have shown wide variation in polyphenol absorption, metabolism, and excretion, which may contribute to differences in study findings (86, 87).

#### Practical implications and future research directions

Based on the present findings and recent international position stand, plant-derived polyphenol supplements may be considered selectively and according to specific recovery needs rather than as routine recovery strategies (8). Across the included trials, supplements were administered before, after, or around exercise as anthocyanin- or flavanol-rich juices, concentrates, powders, capsules, beverages, or extracts. However, substantial variation in product type, dose, timing, and duration indicates that no standardized supplementation strategy has yet been established (Table 1). Current evidence suggests that their potential benefits are supplement-, outcome-, and time-specific and are more consistently observed for DOMS than for recovery as a whole. Tart cherry showed the most consistent favorable pattern for reducing soreness, whereas the findings for pomegranate were less stable. These effects may be relevant when short-term soreness reduction is prioritized, such as during congested competition schedules or limited recovery periods (85). However, improvements in soreness may not reflect broader physiological recovery, as no clear and stable benefits were observed for muscle strength, CK, or CRP.

Given the generally low certainty of evidence, recommendations regarding specific products, doses, timing, duration, or target populations cannot yet be made with confidence. Although multi-day peri-exercise supplementation has been proposed to address the limited bioavailability of some polyphenols, there is insufficient evidence to determine whether it is superior to acute or shorter-duration supplementation (84). The effects of prolonged high-dose supplementation on training adaptation also remain uncertain (8).

Future research should move beyond descriptive findings toward mechanistic investigation and more individualized nutrition strategies. Multi-omics approaches may help characterize interindividual responses and support the development of personalized interventions (88). Future studies may also complement systemic biomarkers such as CK and CRP with quantitative MRI T2 mapping to characterize the regional distribution and time course of muscle changes after EIMD (89, 90). Larger, adequately powered head-to-head trials with well-matched comparator conditions, transparent reporting of blinding procedures, and standardized outcome timing are also needed to clarify the effects of specific plant-derived polyphenol supplements on recovery, training adaptation, and long-term athletic performance.

## Conclusion

This network meta-analysis suggests that plant-derived polyphenol supplements have not shown consistent benefits across all aspects of recovery after exercise-induced muscle damage. Their potential value may lie primarily in reducing DOMS, with tart cherry showing the most consistent favorable pattern and pomegranate showing possible benefit for pain-related recovery. In contrast, no clear or stable benefits were observed for muscle strength recovery or for objective markers such as CK and CRP. Pairwise meta-analyses and sensitivity analyses generally supported this interpretation, although some estimates varied by supplementation protocol, outcome definition, and comparator characteristics. Overall, their potential role appears to be supplement-, outcome-, and time-specific rather than broadly applicable to recovery, particularly for the short-term reduction of perceived soreness. Given the generally low certainty of evidence, recommendations regarding specific products, doses, timing, duration, or target populations cannot yet be made with confidence. Future research should prioritize adequately powered head-to-head trials, standardized intervention protocols, and mechanistic outcomes that better capture local tissue repair, while accounting for individual differences in polyphenol metabolism and comparator design.

## Data Availability

The original contributions presented in the study are included in the article/supplementary material, further inquiries can be directed to the corresponding author.
