# Supplementary material for: Comparative effects of plant-derived anthocyanin- and flavanol-rich supplements on recovery outcomes after exercise-induced muscle damage: a systematic review and network meta-analysis
Source: Front Nutr. 2026 Aug 14;13:1857527. doi: 10.3389/fnut.2026.1857527 (PMC13522125; doi:10.3389/fnut.2026.1857527)
Supplement: Supplementary file 1 [file Data_Sheet_1.pdf]

Supplementary Appendix

Comparative Effects of Plant-Derived Anthocyanin- and Flavanol-Rich Supplements on Recovery Outcomes After Exercise-Induced Muscle Damage: A Systematic Review and Network Meta-Analysis

Kai-Xuan XU[1,2], Juan Chang[1,*], Yi-Jia Wu[1], Hui-Wen Chen[1], Jie-Wei Zheng[1], Chengcheng Liu[3]

Table of contents

Appendix 1: Search strategy 2

Appendix 2: Variables extracted from the included studies........ 6

Appendix 3: Treatment node definitions and study allocation in the network meta-analysis 7

Appendix 4: Characteristics and classification of placebo and control conditions........ 8

Appendix 5: Risk of bias of randomized clinical trials........ 13

Appendix 6: Evaluation of inconsistency and heterogeneity 15

Appendix 7: Conventional pairwise random-effects meta-analyses for the main 48-h outcomes........ 16

Appendix 8:Network maps and forest plots of secondary outcomes additional time points 18

Appendix 9: SUCRA and cumulative probability plots........ 26

Appendix 10: League tables of summary estimates from the network meta-analysis........ 38

Appendix 11: CINeMA Assessment 50

Appendix 12: Funnel plots........ 62

Appendix 13: Sensitivity analyses 69

Appendix 14: Subgroup analysis of training status........ 74

Appendix 1: Search strategy

Table S1. Search strategy of PubMed

#	Searches
**1**	("Anthocyanins"[MeSH Terms] OR "Flavonoids"[MeSH Terms] OR anthocyanin*[Title/Abstract] OR anthocyanidin*[Title/Abstract] OR flavanol*[Title/Abstract] OR "flavan-3-ol"[Title/Abstract] OR flavonoid*[Title/Abstract] OR polyphenol*[Title/Abstract] OR phenolic*[Title/Abstract] OR proanthocyanidin*[Title/Abstract] OR "plant extract"[Title/Abstract] OR "fruit extract"[Title/Abstract] OR "berry extract"[Title/Abstract] OR "tart cherry"[Title/Abstract] OR "sour cherry"[Title/Abstract] OR "cherry juice"[Title/Abstract] OR "Montmorency cherry"[Title/Abstract] OR pomegranate[Title/Abstract] OR blueberry[Title/Abstract] OR blackcurrant[Title/Abstract] OR elderberry[Title/Abstract] OR bilberry[Title/Abstract] OR aronia[Title/Abstract] OR "Aronia melanocarpa"[Title/Abstract] OR cocoa[Title/Abstract] OR "dark chocolate"[Title/Abstract] OR "green tea"[Title/Abstract] OR "black tea"[Title/Abstract] OR EGCG[Title/Abstract] OR epigallocatechin*[Title/Abstract] OR catechin*[Title/Abstract] OR pycnogenol[Title/Abstract] OR "pine bark"[Title/Abstract] OR grape[Title/Abstract] OR "grape seed extract"[Title/Abstract])
**2**	("exercise-induced muscle damage"[Title/Abstract] OR EIMD[Title/Abstract] OR "delayed onset muscle soreness"[Title/Abstract] OR DOMS[Title/Abstract] OR "muscle soreness"[Title/Abstract] OR "muscle damage"[Title/Abstract] OR "muscle recovery"[Title/Abstract] OR "muscle function"[Title/Abstract] OR "muscle strength"[Title/Abstract] OR "maximal voluntary contraction"[Title/Abstract] OR "peak torque"[Title/Abstract] OR "range of motion"[Title/Abstract] OR "countermovement jump"[Title/Abstract] OR "creatine kinase"[Title/Abstract] OR "lactate dehydrogenase"[Title/Abstract] OR myoglobin[Title/Abstract] OR "interleukin-6"[Title/Abstract] OR "IL-6"[Title/Abstract] OR "C-reactive protein"[Title/Abstract] OR "TNF-α"[Title/Abstract] OR "oxidative stress"[Title/Abstract] OR "malondialdehyde"[Title/Abstract] OR "eccentric exercise"[Title/Abstract] OR "eccentric contraction"[Title/Abstract] OR "downhill running"[Title/Abstract] OR "damaging exercise"[Title/Abstract])
**3**	("randomized controlled trial"[Publication Type] OR "controlled clinical trial"[Publication Type] OR randomized[Title/Abstract] OR randomised[Title/Abstract] OR placebo[Title/Abstract] OR crossover[Title/Abstract] OR "cross-over"[Title/Abstract] OR "double-blind"[Title/Abstract])
**4**	("systematic review"[Title/Abstract] OR meta-analy*[Title/Abstract] OR "systematic review"[Publication Type])
**5**	#1 AND #2 AND #3
**6**	#5 NOT #4
**7**	#6 AND "humans"[MeSH Terms] AND "english"[Language]
**8**	#7 NOT ("animals"[MeSH Terms] NOT "humans"[MeSH Terms])

Table S2. Search strategy of Web of Science: Science Citation Index Expanded

#	Searches
**1**	TS=(anthocyanin* OR anthocyanidin* OR flavanol* OR "flavan-3-ol" OR "flavan-3-ols" OR flavonoid* OR polyphenol* OR phenolic* OR proanthocyanidin* OR "plant extract*" OR "fruit extract*" OR "berry extract*" OR "tart cherry" OR "sour cherry" OR "cherry juice" OR "Montmorency cherry" OR pomegranate OR blueberry OR blackcurrant OR elderberry OR bilberry OR aronia OR "Aronia melanocarpa" OR cocoa OR "dark chocolate" OR "green tea" OR "black tea" OR EGCG OR epigallocatechin* OR catechin* OR pycnogenol OR "pine bark" OR "grape seed extract*" OR "grape polyphenol*" OR "grape juice")
**2**	TS=("exercise-induced muscle damage" OR EIMD OR "delayed onset muscle soreness" OR DOMS OR "muscle soreness" OR "muscle damage" OR "muscle recovery" OR "muscle function" OR "muscle strength" OR "maximal voluntary contraction" OR "peak torque" OR "range of motion" OR "countermovement jump" OR "creatine kinase" OR "lactate dehydrogenase" OR myoglobin OR "interleukin-6" OR "IL-6" OR "IL 6" OR "C-reactive protein" OR CRP OR "TNF-alpha" OR "TNF alpha" OR "tumor necrosis factor" OR "oxidative stress" OR malondialdehyde OR "eccentric exercise" OR "eccentric contraction*" OR "eccentric training" OR "downhill running" OR "damaging exercise" OR "strenuous exercise")
**3**	TS=("randomized controlled trial*" OR "controlled clinical trial*" OR randomized OR randomised OR "randomly allocated" OR "randomly assigned" OR "double-blind*" OR "double blind*" OR "placebo-controlled" OR "placebo controlled" OR crossover OR "cross-over" OR "cross over") OR DT=("Randomized Controlled Trial")
**4**	TS=("systematic review" OR meta-analy* OR "meta-analysis") OR DT=(Review OR "Systematic Review")
**5**	#1 AND #2
**6**	#5 AND #3
**7**	#6 NOT #4
**8**	#7 AND LA=(English)

Table S3. Search strategy of Cochrane Central Register of Controlled Trials (CENTRAL)

#	Searches
**1**	[mh "Anthocyanins"] OR [mh "Flavonoids"] OR anthocyanin*:ti,ab,kw OR anthocyanidin*:ti,ab,kw OR flavanol*:ti,ab,kw OR "flavan-3-ol":ti,ab,kw OR "flavan-3-ols":ti,ab,kw OR flavonoid*:ti,ab,kw OR polyphenol*:ti,ab,kw OR phenolic*:ti,ab,kw OR proanthocyanidin*:ti,ab,kw OR "plant extract":ti,ab,kw OR "fruit extract":ti,ab,kw OR "berry extract":ti,ab,kw OR "tart cherry":ti,ab,kw OR "sour cherry":ti,ab,kw OR "cherry juice":ti,ab,kw OR "Montmorency cherry":ti,ab,kw OR pomegranate:ti,ab,kw OR blueberry:ti,ab,kw OR blackcurrant:ti,ab,kw OR elderberry:ti,ab,kw OR bilberry:ti,ab,kw OR aronia:ti,ab,kw OR "Aronia melanocarpa":ti,ab,kw OR cocoa:ti,ab,kw OR "dark chocolate":ti,ab,kw OR "green tea":ti,ab,kw OR "black tea":ti,ab,kw OR EGCG:ti,ab,kw OR epigallocatechin*:ti,ab,kw OR catechin*:ti,ab,kw OR pycnogenol:ti,ab,kw OR "pine bark":ti,ab,kw OR "grape seed extract":ti,ab,kw OR "grape polyphenol":ti,ab,kw OR "grape juice":ti,ab,kw
**2**	[mh "Muscle, Skeletal"] OR [mh "Exercise"] OR "exercise-induced muscle damage":ti,ab,kw OR EIMD:ti,ab,kw OR "delayed onset muscle soreness":ti,ab,kw OR DOMS:ti,ab,kw OR "muscle soreness":ti,ab,kw OR "muscle damage":ti,ab,kw OR "muscle recovery":ti,ab,kw OR "muscle function":ti,ab,kw OR "muscle strength":ti,ab,kw OR "maximal voluntary contraction":ti,ab,kw OR "peak torque":ti,ab,kw OR "range of motion":ti,ab,kw OR "countermovement jump":ti,ab,kw OR "creatine kinase":ti,ab,kw OR "lactate dehydrogenase":ti,ab,kw OR myoglobin:ti,ab,kw OR "interleukin-6":ti,ab,kw OR "IL-6":ti,ab,kw OR "C-reactive protein":ti,ab,kw OR "tumor necrosis factor":ti,ab,kw OR "TNF-alpha":ti,ab,kw OR "oxidative stress":ti,ab,kw OR malondialdehyde:ti,ab,kw OR "eccentric exercise":ti,ab,kw OR "eccentric contraction":ti,ab,kw OR "eccentric training":ti,ab,kw OR "downhill running":ti,ab,kw OR "damaging exercise":ti,ab,kw OR "strenuous exercise":ti,ab,kw
**3**	randomized:ti,ab,kw OR randomised:ti,ab,kw OR placebo:ti,ab,kw OR crossover:ti,ab,kw OR "cross-over":ti,ab,kw OR (double NEXT blind*):ti,ab,kw
**4**	#1 AND #2
**5**	#4 AND #3
**6**	"systematic review":ti,ab,kw OR meta-analy*:ti,ab,kw
**7**	#5 NOT #6

Table S4. Search strategy of Embase

#	Searches
**1**	'anthocyanin'/exp OR 'flavonoid'/exp OR 'polyphenol'/exp OR 'catechin'/exp OR 'proanthocyanidin'/exp OR 'epigallocatechin gallate'/exp OR anthocyanin*:ti,ab OR anthocyanidin*:ti,ab OR flavanol*:ti,ab OR 'flavan-3-ol':ti,ab OR 'flavan-3-ols':ti,ab OR flavonoid*:ti,ab OR polyphenol*:ti,ab OR phenolic*:ti,ab OR proanthocyanidin*:ti,ab OR 'plant extract*':ti,ab OR 'fruit extract*':ti,ab OR 'berry extract*':ti,ab OR 'tart cherry':ti,ab OR 'sour cherry':ti,ab OR 'cherry juice':ti,ab OR 'Montmorency cherry':ti,ab OR pomegranate:ti,ab OR blueberry:ti,ab OR blackcurrant:ti,ab OR elderberry:ti,ab OR bilberry:ti,ab OR aronia:ti,ab OR 'Aronia melanocarpa':ti,ab OR cocoa:ti,ab OR 'dark chocolate':ti,ab OR 'green tea':ti,ab OR 'black tea':ti,ab OR EGCG:ti,ab OR epigallocatechin*:ti,ab OR catechin*:ti,ab OR pycnogenol:ti,ab OR 'pine bark':ti,ab OR 'grape seed extract*':ti,ab OR 'grape polyphenol*':ti,ab OR 'grape juice':ti,ab
**2**	'skeletal muscle'/exp OR 'muscle injury'/exp OR 'muscle fatigue'/exp OR 'delayed onset muscle soreness'/exp OR 'creatine kinase'/exp OR 'interleukin 6'/exp OR 'C-reactive protein'/exp OR 'tumor necrosis factor'/exp OR 'malondialdehyde'/exp OR 'oxidative stress'/exp OR 'exercise-induced muscle damage':ti,ab OR EIMD:ti,ab OR 'delayed onset muscle soreness':ti,ab OR DOMS:ti,ab OR 'muscle soreness':ti,ab OR 'muscle damage':ti,ab OR 'muscle recovery':ti,ab OR 'muscle function':ti,ab OR 'muscle strength':ti,ab OR 'maximal voluntary contraction':ti,ab OR 'peak torque':ti,ab OR 'range of motion':ti,ab OR 'countermovement jump':ti,ab OR 'creatine kinase':ti,ab OR 'lactate dehydrogenase':ti,ab OR myoglobin:ti,ab OR 'interleukin-6':ti,ab OR 'IL-6':ti,ab OR 'C-reactive protein':ti,ab OR 'tumor necrosis factor':ti,ab OR 'TNF-alpha':ti,ab OR 'TNF alpha':ti,ab OR 'oxidative stress':ti,ab OR malondialdehyde:ti,ab OR 'eccentric exercise':ti,ab OR 'eccentric contraction*':ti,ab OR 'eccentric training':ti,ab OR 'downhill running':ti,ab OR 'damaging exercise':ti,ab OR 'strenuous exercise':ti,ab
**3**	'randomized controlled trial'/exp OR 'controlled clinical trial'/exp OR 'crossover procedure'/exp OR 'double blind procedure'/exp OR 'placebo'/exp OR randomized:ti,ab OR randomised:ti,ab OR placebo:ti,ab OR crossover:ti,ab OR 'cross-over':ti,ab OR 'double-blind':ti,ab OR 'double blind':ti,ab OR 'randomly allocated':ti,ab OR 'randomly assigned':ti,ab
**4**	'systematic review'/exp OR 'meta analysis'/exp OR 'systematic review':ti,ab OR meta-analy*:ti,ab
**5**	#1 AND #2
**6**	#5 AND #3
**7**	#6 NOT #4
**8**	#7 AND [humans]/lim AND [english]/lim

Appendix 2: Variables extracted from the included studies

Data category	List of variables
Study	First author, year of publication, country/region, study design, sample size, and treatment period used for crossover trials
Participant characteristics	Sex, age, training status, athletic level, and other baseline characteristics if reported
EIMD protocol	Standardised EIMD category, specific exercise protocol, exercise intensity/load, muscle group, and exercise duration/volume
Intervention and comparator characteristics	Plant source, final NMA node, polyphenol class, main active compound(s), product form, dose, supplementation timing, intervention duration, and comparator type, placebo or control composition, including ingredients, energy content, macronutrient profile, colour, flavour, appearance, and volume where reported; matching characteristics between intervention and comparator products; blinding procedures
Outcomes	Outcome domain, instrument/measure, assessment time points, analysed sample size, direction of measurement and reverse-coding decisions where applicable, and extractable statistics for each arm, including delayed onset muscle soreness, muscle function recovery, biomarkers of muscle damage, inflammation, oxidative stress, and other recovery-related outcomes if reported.
Additional methodological information	Randomisation, blinding, attrition, and other methodological notes relevant to sensitivity analyses or node classification

Appendix 3: Treatment node definitions and study allocation in the network meta-analysis

Plant Source / Extract Type	Main Active Compound(s)	Product Forms Used Across Studies	No. of Studies	Studies Included (Author Year)
Tart Cherry	Anthocyanins; polyphenols	Concentrate juice / reconstituted concentrate (×7); juice / ready-to-drink beverage (×1); gel (×1); freeze-dried or concentrated extract capsules/powder (×3); spray-dried extract capsules (×1)	13	Bell 2015; Bell 2016; Bowtell 2011; Brown 2019; Hillman 2017; Kupusarevic 2019; Levers 2015; Levers 2016; McCormick 2016; Morehen 2021; Quinlan 2020; Ortega 2023; Squires 2026
Pomegranate	Punicalagins; tannins; anthocyanins; ellagic acid derivatives; total polyphenols	Juice / beverage formulations (×3); extract capsules (×1)	4	Ammar 2016; Trombold 2011; Torregrosa-García 2019; Trombold 2010
Tea Extract	Catechins (including EGCG); theaflavins; tea polyphenols	Capsules (×5)	5	Arent 2010; Beyer 2017; Jajtner 2016; Jówko 2012; Jówko 2015
Blueberry	Anthocyanins; polyphenols	Freeze-dried powder (×1); smoothie / blueberry beverage (×1)	2	McLeay 2012; Nieman 2023
Blackcurrant	Anthocyanins	Capsules (×1); juice / nectar (×1)	2	Costello 2020; Hutchison 2016
Cocoa Flavanols	Flavanols	Cocoa / cacao drink (×1); flavanol supplement combined with milk beverage (×1)	2	de Carvalho 2019; Morgan 2018
Grape	Polyphenols	Freeze-dried powder in water (×1)	1	O'Connor 2013
Mixed Polyphenols	Trans-resveratrol; catechins; anthocyanins; flavonols; quercetin	Capsules (×1)	1	Jo 2019

Appendix 4: Characteristics and classification of placebo and control conditions

Study	Intervention	Control condition	Energy matching	Macronutrient matching	Color/flavor /appearance matching	Vehicle matching	Blinding	Matching with the intervention group	Energy content category
Ammar 2016	Pomegranate juice	Commercial pomegranate -flavored beverage (water, citric acid, flavoring, sweetener, and gum arabic; no polyphenols)	NR	NR	Yes (flavor)	Yes	NR	Partially matched or unclear comparator	Minimal-nutrient placebo
Arent 2010	Theaflavin-enriched black tea extract capsules	Placebo capsules	N/A (capsule)	N/A (capsule)	Yes (appearance)	Yes	Double-blind	Inert or minimal-nutrient placebo	Minimal-nutrient placebo
Bell 2015	Montmorency tart cherry juice concentrate	Mixed-berry juice concentrate (<5% fruit juice) + water + maltodextrin	Yes	Yes (carbohydrate matched)	Yes	Yes	Double-blind	Energy, carbohydrate, and sensory matched comparator	Energy-containing placebo
Bell 2016	Montmorency tart cherry juice concentrate	Fruit syrup (<5% fruit juice) + water + maltodextrin	Yes	Yes (carbohydrate matched)	Yes	Yes	Double-blind	Energy, carbohydrate, and sensory matched comparator	Energy-containing placebo
Beyer 2017	Tea polyphenol complex capsules	Microcrystalline cellulose placebo capsules, similar in shape and size to the intervention capsules and administered at the same dose	N/A (capsule)	N/A (capsule)	Yes (appearance)	Yes	Double-blind	Inert or minimal-nutrient placebo	Minimal-nutrient placebo

Bowtell 2011	Montmorency tart cherry juice concentrate	Synthetic fruit juice concentrate	Yes	Partial: primarily carbohydrate matched through isoenergetic design; exact placebo macronutrient values NR	Yes	Yes	Double-blind	Energy, carbohydrate, and sensory matched comparator	Energy-containing placebo
Brown 2019	Montmorency tart cherry juice concentrate	Artificial fruit-flavored beverage + maltodextrin + unflavored whey protein	Yes	Yes	Yes	Yes	Double-blind	Energy, carbohydrate, and sensory matched comparator	Energy-containing placebo
Costello 2020	New Zealand blackcurrant extract capsules	Microcrystalline cellulose (2 × 300 mg capsules)	N/A (capsule)	N/A (capsule)	Yes (appearance)	Yes	Double-blind	Inert or minimal-nutrient placebo	Minimal-nutrient placebo
de Carvalho 2019	Low-fat chocolate milk supplemented with cocoa flavanols	Plain low-fat chocolate milk (the intervention group received additional cocoa flavanols in the same base)	Yes	Yes	Yes	Yes (same base vehicle)	Double-blind	Energy, carbohydrate, and sensory matched comparator	Protein, milk, or smoothie based control
Hillman 2017	Tart cherry juice plus whey protein beverage	Cherry-flavored Kool-Aid beverage with pure cane sugar	No: 97 kcal per 240 mL in placebo vs approximately 160 kcal per	No: placebo contained 24 g sugar and 0 g protein vs intervention with 27 g sugar and 8 g protein	Partial: cherry flavor matched; color and appearance NR	Yes	Single-blind	Sensory matched but nutritionally non-equivalent comparator	Nutrient-mismatched control

			240 mL in interventi on						
Hutchiso n 2016	Blackcurrant nectar	Black cherry Kool-Aid powder mixed with water + added sucrose	Yes	Yes	Yes	Yes	Double-blind	Energy, carbohydrate, and sensory matched comparator	Energy-containing placebo
Jajtner 2016	Green and black tea polyphenol extract capsules	Microcrystalline cellulose placebo capsules, similar in shape, color, and size to the intervention capsules and administered at the same dose	N/A (capsule)	N/A (capsule)	Yes (appearance)	Yes	Blinding maintained; blinding level NR	Inert or minimal-nutrient placebo	Minimal-nutrient placebo
Jo 2019	Trans-resveratrol and mixed polyphenol extract capsules	Placebo capsules	Yes	N/A (capsule)	Yes	Yes	Double-blind	Inert or minimal-nutrient placebo	Minimal-nutrient placebo
Jówko 2012	Green tea polyphenol capsules	Capsules containing microcrystalline cellulose + magnesium stearate + maltodextrin	N/A (capsule)	N/A (capsule)	Yes (appearance)	Yes	Double-blind	Inert or minimal-nutrient placebo	Minimal-nutrient placebo
Jówko 2015	Green tea extract capsules	Dark gelatin placebo capsules containing microcrystalline cellulose, magnesium stearate, and maltodextrin; identical in size, shape, and color to the intervention capsules, with the same dosing regimen	N/A (capsule)	N/A (capsule)	Yes (appearance)	Yes	Double-blind	Inert or minimal-nutrient placebo	Minimal-nutrient placebo

Kupusarevic 2019	Tart cherry gel	Commercial cherry-flavored isotonic energy gel	Yes	Yes (carbohydrate and protein)	Yes	Yes	Double-blind	Energy, carbohydrate, and sensory matched comparator	Energy-containing placebo
Levers 2015	Montmorency tart cherry powder capsules	Rice flour capsules	N/A (capsule)	N/A (capsule)	Yes (appearance)	Yes	Double-blind	Inert or minimal-nutrient placebo	Minimal-nutrient placebo
Levers 2016	Montmorency tart cherry powder capsules	Rice flour capsules	N/A (capsule)	N/A (capsule)	Yes	Yes	Double-blind	Inert or minimal-nutrient placebo	Minimal-nutrient placebo
Trombold 2011	Pomegranate juice	Isoenergetic flavored placebo beverage containing maltodextrin and sucralose	Yes	NR	NR	Yes	Double-blind	Energy, carbohydrate, and sensory matched comparator	Energy-containing placebo
McCormick 2016	Montmorency tart cherry juice concentrate	Lime/cranberry/raspberry mixed syrup + water + maltodextrin + artificial coloring	NR	Yes	Yes	Yes	Double-blind	Energy, carbohydrate, and sensory matched comparator	Energy-containing placebo
McLeay 2012	Blueberry smoothie	Smoothie base (banana + apple juice + dextrose; no blueberries)	Yes	Yes	NR	Yes	NR	Partially matched or unclear comparator	Protein, milk, or smoothie based control
Morehen 2021	Montmorency tart cherry juice concentrate	Fruit syrup + water + maltodextrin	Yes	Yes	Yes	Yes	Single-blind	Energy, carbohydrate, and sensory matched comparator	Energy-containing placebo

Morgan 2018	Flavanol-rich cocoa pulp juice	Glucose + invert sugar syrup + water + citric acid + flavoring + coloring	Yes	Yes	Yes	Yes	Double-blind	Energy, carbohydrate, and sensory matched comparator	Energy-containing placebo
O'Connor 2013	Freeze-dried grape powder beverage	Powder mixture of starch, sugar, cellulose, acid, grape flavoring, and food coloring	Yes	Yes	Yes	Yes	Double-blind	Energy, carbohydrate, and sensory matched comparator	Energy-containing placebo
Torregrosa-García 2019	Pomegranate extract capsules	Maltodextrin capsules	N/A (capsule)	N/A (capsule)	Yes (appearance)	Yes	Double-blind	Inert or minimal-nutrient placebo	Minimal-nutrient placebo
Trombold 2010	Pomegranate ellagitannin extract beverage	Maltodextrin + sucralose + water + flavoring + coloring	NR	Yes (carbohydrate)	Yes	Yes	Double-blind	Energy, carbohydrate, and sensory matched comparator	Energy-containing placebo
Nieman 2023	Freeze-dried blueberry powder	Placebo powder (mixed into water or food for consumption)	NR	Yes (carbohydrate/sugar)	Yes	Yes	Double-blind	Energy, carbohydrate, and sensory matched comparator	Energy-containing placebo
Quinlan 2020	Montmorency tart cherry juice concentrate	Commercial beverage containing <5% fruit juice + water + maltodextrin	NR	Partially matched (carbohydrate)	Yes	Yes	Single-blind	Energy, carbohydrate, and sensory matched comparator	Energy-containing placebo
Ortega 2023	Tart cherry supplement capsules	Capsules containing dextrose + natural red coloring	N/A (capsule)	N/A (capsule)	Yes (appearance)	Yes	Double-blind	Inert or minimal-nutrient placebo	Minimal-nutrient placebo
Squires 2026	Vistula tart cherry extract capsules	Placebo capsules	Yes	N/A (capsule)	Yes (appearance)	Yes	Double-blind	Inert or minimal-nutrient placebo	Minimal-nutrient placebo

Note: *1. Abbreviations: N/A, not applicable; NR, not reported.*
2. For capsule formulations, when the nutritional contributions of both the intervention and placebo are negligible, energy and macronutrient matching are coded as "N/A"; comparability is assessed primarily on capsule appearance, dose, and vehicle.
3. "Partially matched" indicates that only one macronutrient component (usually carbohydrate) was matched and equivalence of the complete macronutrient composition could not be established.

Appendix 5: Risk of bias of randomized clinical trials

Figure S5: Distribution of RoB 2 judgments across included studies by bias domain (% of studies). Green = low risk; yellow = some concerns; red = high risk.

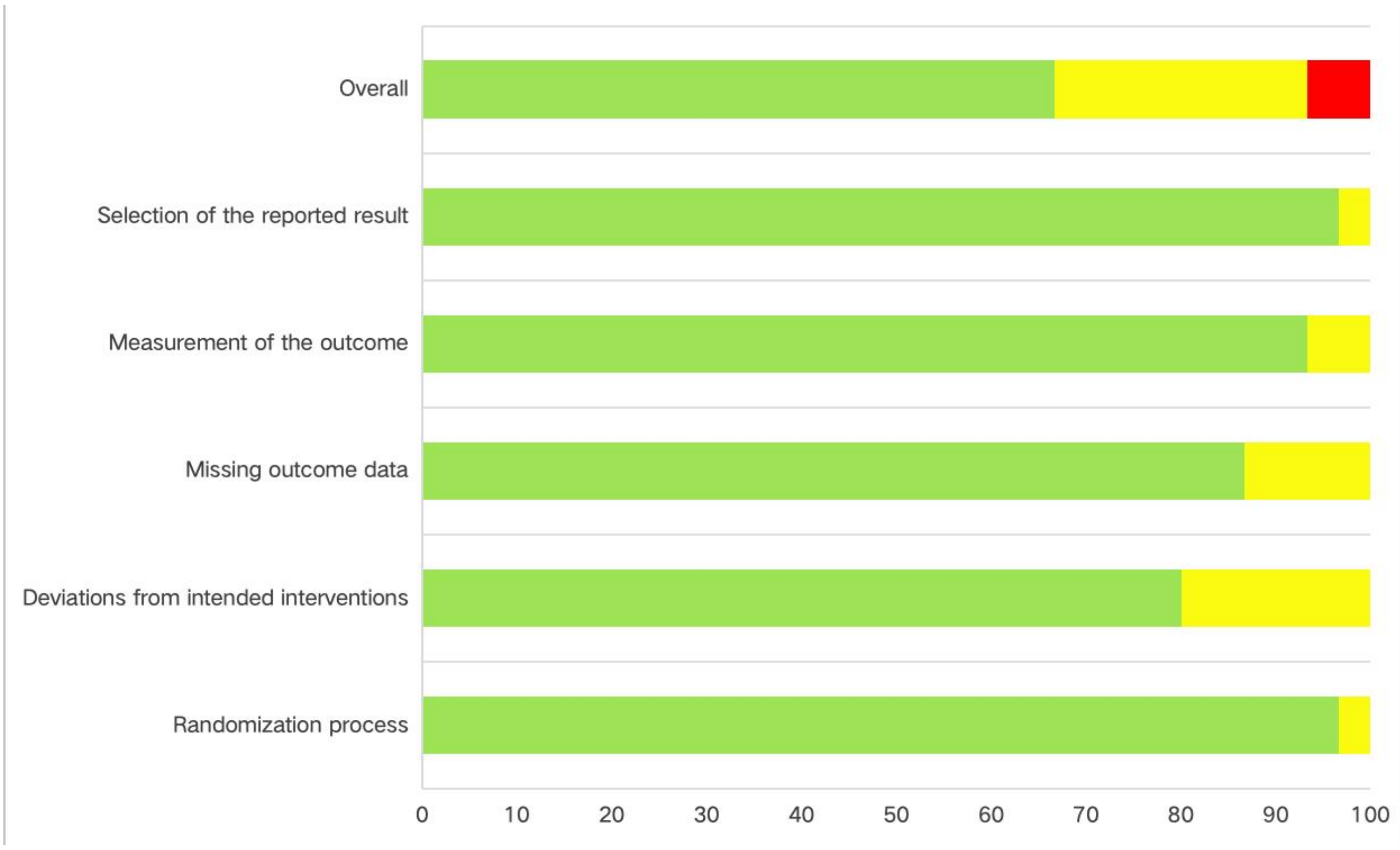

Table S5: Study-level risk of bias assessments for the included randomized controlled trials, conducted using the Cochrane Risk of Bias 2 tool.

Unique ID	Randomization process	Deviations from intended interventions	Missing outcome data	Measuremen t of the outcome	Selection of the reported result	Over all
Ammar 2016	Some concerns	Some concerns	Low	Some concerns	Low	High
Arent 2010	Low	Low	Low	Low	Low	Low
Bell 2015	Low	Low	Low	Low	Low	Low
Bell 2016	Low	Low	Low	Low	Low	Low
Beyer 2017	Low	Low	Some concerns	Low	Low	Some concerns
Bowtell 2011	Low	Low	Low	Low	Low	Low
Brown 2019	Low	Low	Low	Low	Low	Low
Costello 2020	Low	Low	Low	Low	Low	Low
de Carvalho 2019	Low	Low	Some concerns	Some concerns	Some concerns	High
Hillman 2017	Low	Some concerns	Low	Low	Low	Some concerns
Hutchison 2016	Low	Low	Low	Low	Low	Low
Jajtner 2016	Low	Low	Low	Low	Low	Low
Jo 2019	Low	Low	Low	Low	Low	Low
Jówko 2012	Low	Low	Low	Low	Low	Low
Jówko 2015	Low	Low	Low	Low	Low	Low
Kupusarevic 2019	Low	Some concerns	Low	Low	Low	Some concerns
Levers 2015	Low	Low	Some concerns	Low	Low	Some concerns
Levers 2016	Low	Low	Low	Low	Low	Low
Trombold 2011	Low	Low	Low	Low	Low	Low
McCormick 2016	Low	Low	Low	Low	Low	Low
McLeay 2012	Low	Some concerns	Low	Low	Low	Some concerns
Morehen 2021	Low	Some concerns	Low	Low	Low	Some concerns
Morgan 2018	Low	Low	Low	Low	Low	Low
O'Connor 2013	Low	Low	Low	Low	Low	Low
Torregrosa-García 2019	Low	Low	Low	Low	Low	Low
Trombold 2010	Low	Low	Some concerns	Low	Low	Some concerns
Nieman 2023	Low	Low	Low	Low	Low	Low
Quinlan 2020	Low	Some concerns	Low	Low	Low	Some concerns
Ortega 2023	Low	Low	Low	Low	Low	Low
Squires 2026	Low	Low	Low	Low	Low	Low

Appendix 6: Evaluation of inconsistency and heterogeneity

Table S6: Assessment of network structure and heterogeneity for each outcome and timepoint

Outcome	Timepoint	Chi square	P value	Tau²
DOMS	24 h	0.14	0.7056	<0.04
DOMS	48 h	0.03	0.8618	0.13
DOMS	72 h	0.05	0.8205	0.12
Muscle strength/ torque	24 h	0.54	0.4604	<0.04
Muscle strength/ torque	48 h	0.13	0.7139	0.13
Muscle strength/ torque	72 h	1.85	0.1743	<0.04
CK	24 h	0.17	0.6786	<0.04
CK	48 h	0.00	1.0000	0.06
CK	72 h	0.82	0.3646	0.11
CRP	24 h	0.19	0.6657	0.07
CRP	48 h	1.17	0.2797	<0.04
CRP	72 h	0.83	0.3617	<0.04

Note: Chi-square and P values were derived from the global inconsistency test; Tau² represents the between-study heterogeneity estimate for each outcome and timepoint.

Appendix 7: Conventional pairwise random-effects meta-analyses for the main 48-h outcomes

Figure S7.1: Conventional pairwise random-effects meta-analysis of 48-h DOMS. Negative SMD values favour plant-derived supplements.

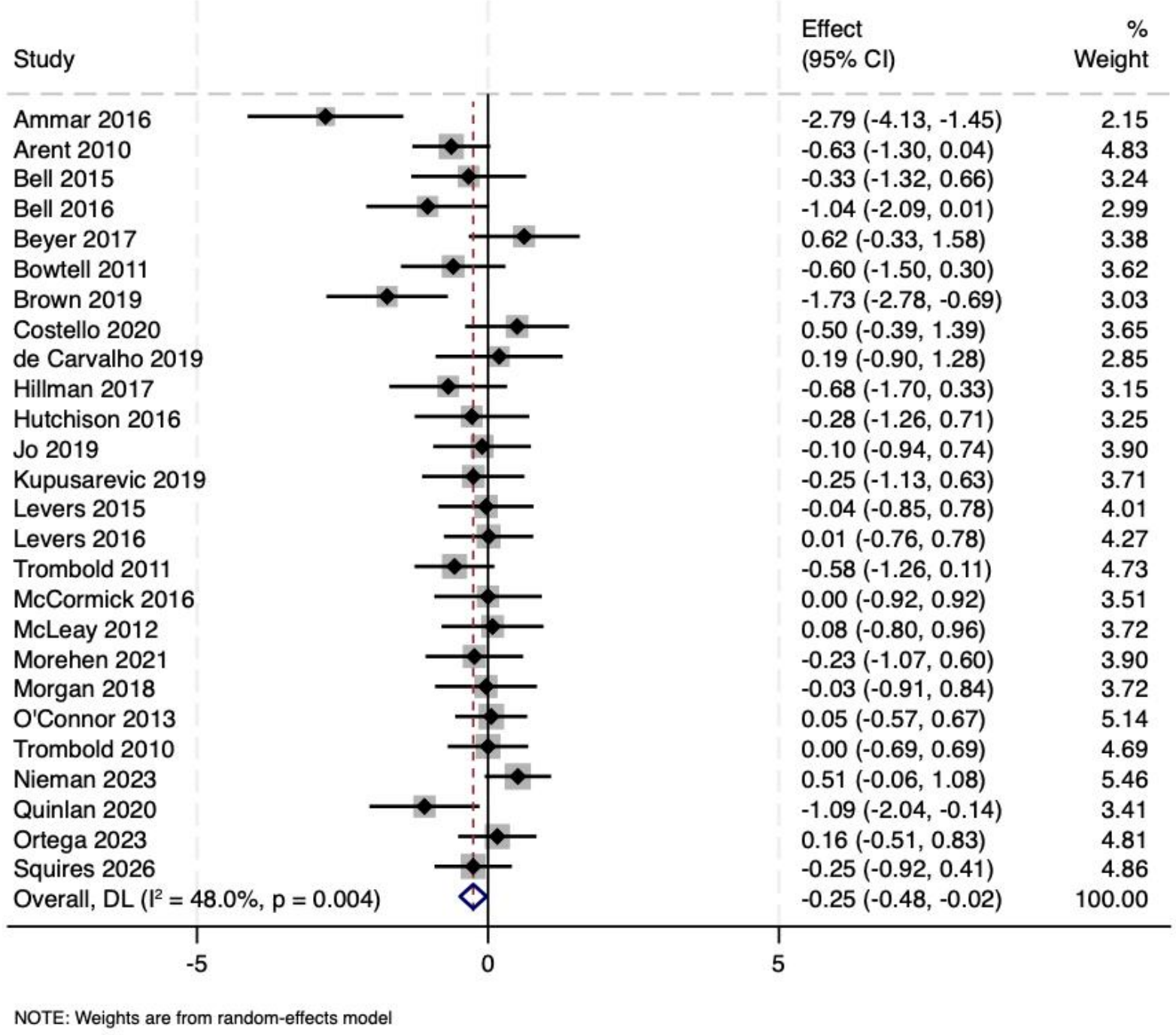

Figure S7.2: Conventional pairwise random-effects meta-analysis of 48-h muscle strength-related outcomes. Positive SMD values favour plant-derived supplements.

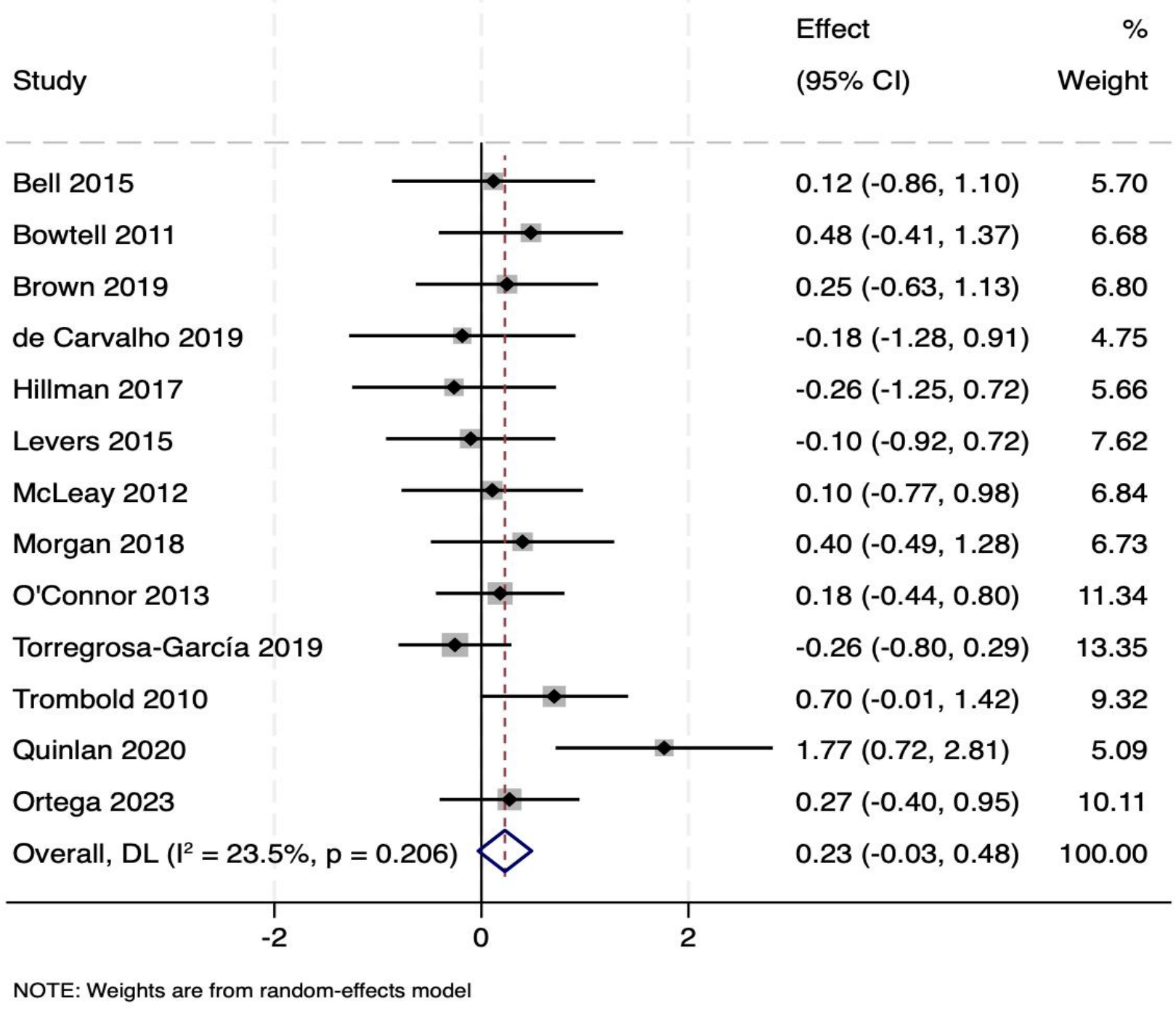

Figure S7.3: Conventional pairwise random-effects meta-analysis of 48-h CK. Negative SMD values favour plant-derived supplements.

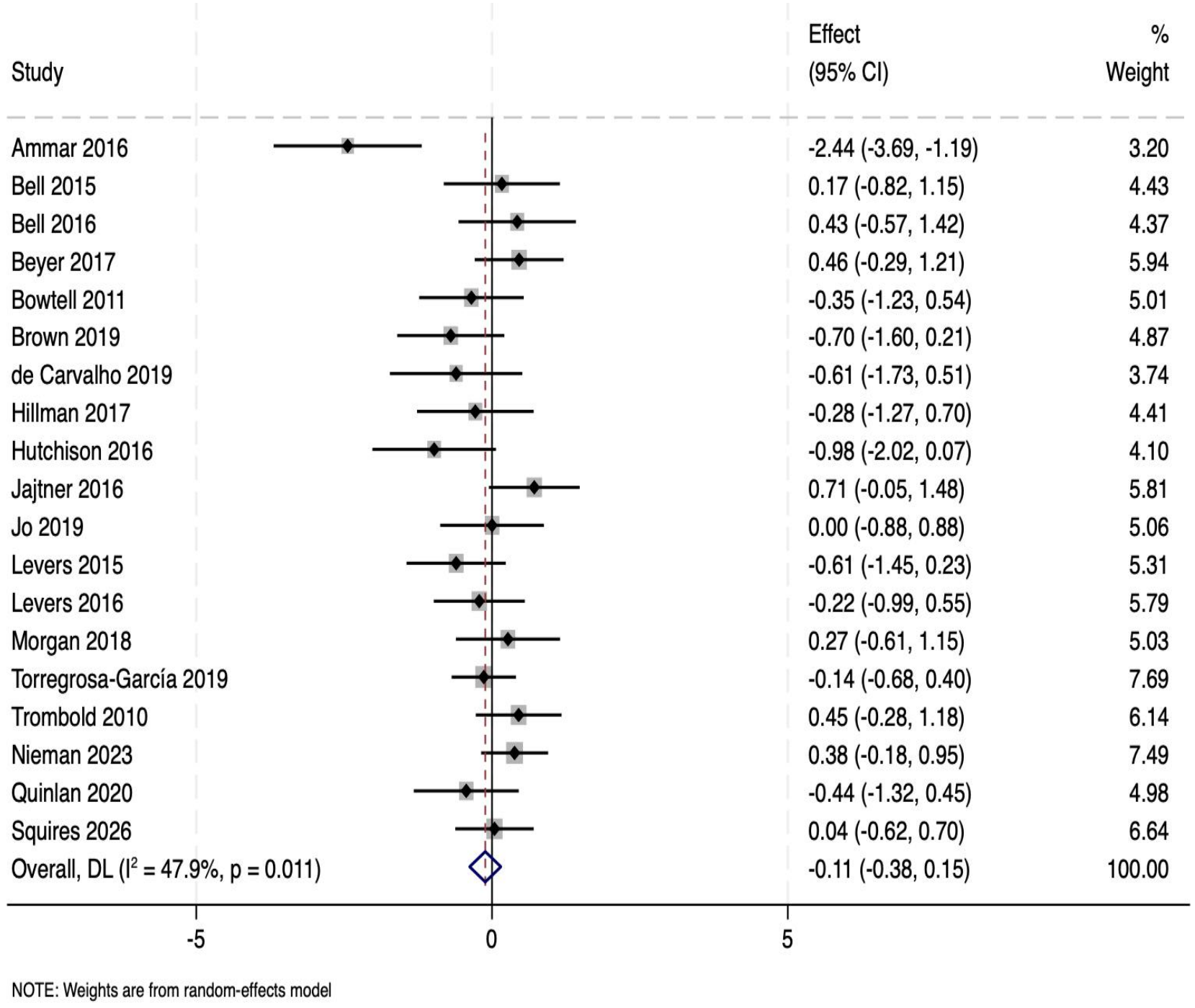

Figure S7.4: Conventional pairwise random-effects meta-analysis of 48-h CRP. Negative SMD values favour plant-derived supplements.

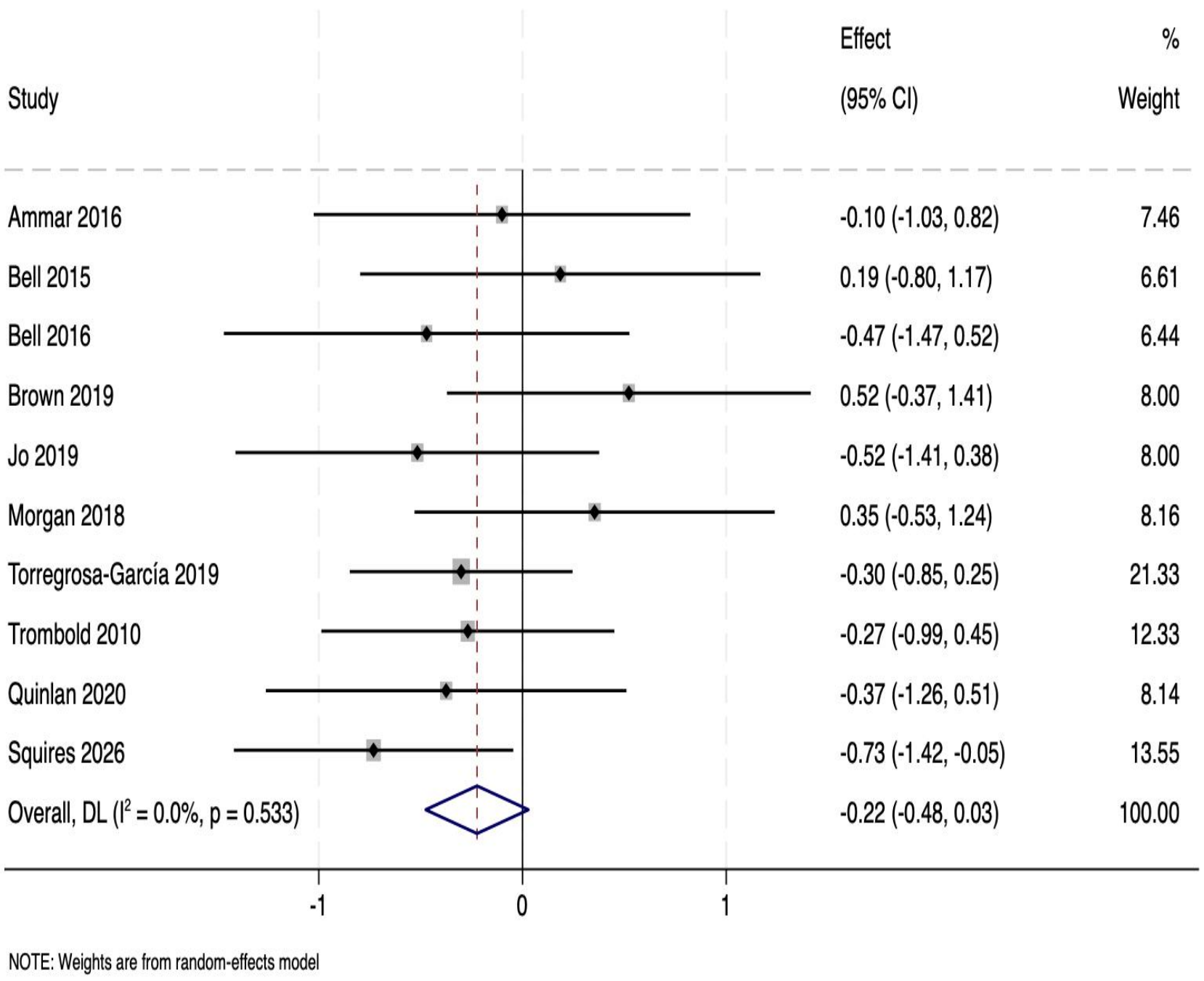

Appendix 8: Network maps and forest plots of secondary outcomes additional time points

Figure S8.1: Network plot and forest plot for 24-h DOMS. Node size is proportional to the number of participants, and edge thickness is proportional to the number of studies for each comparison. The placebo node pools all eligible control conditions, including matched placebo and vehicle-matched controls. Negative SMD values favour plant-derived supplements.

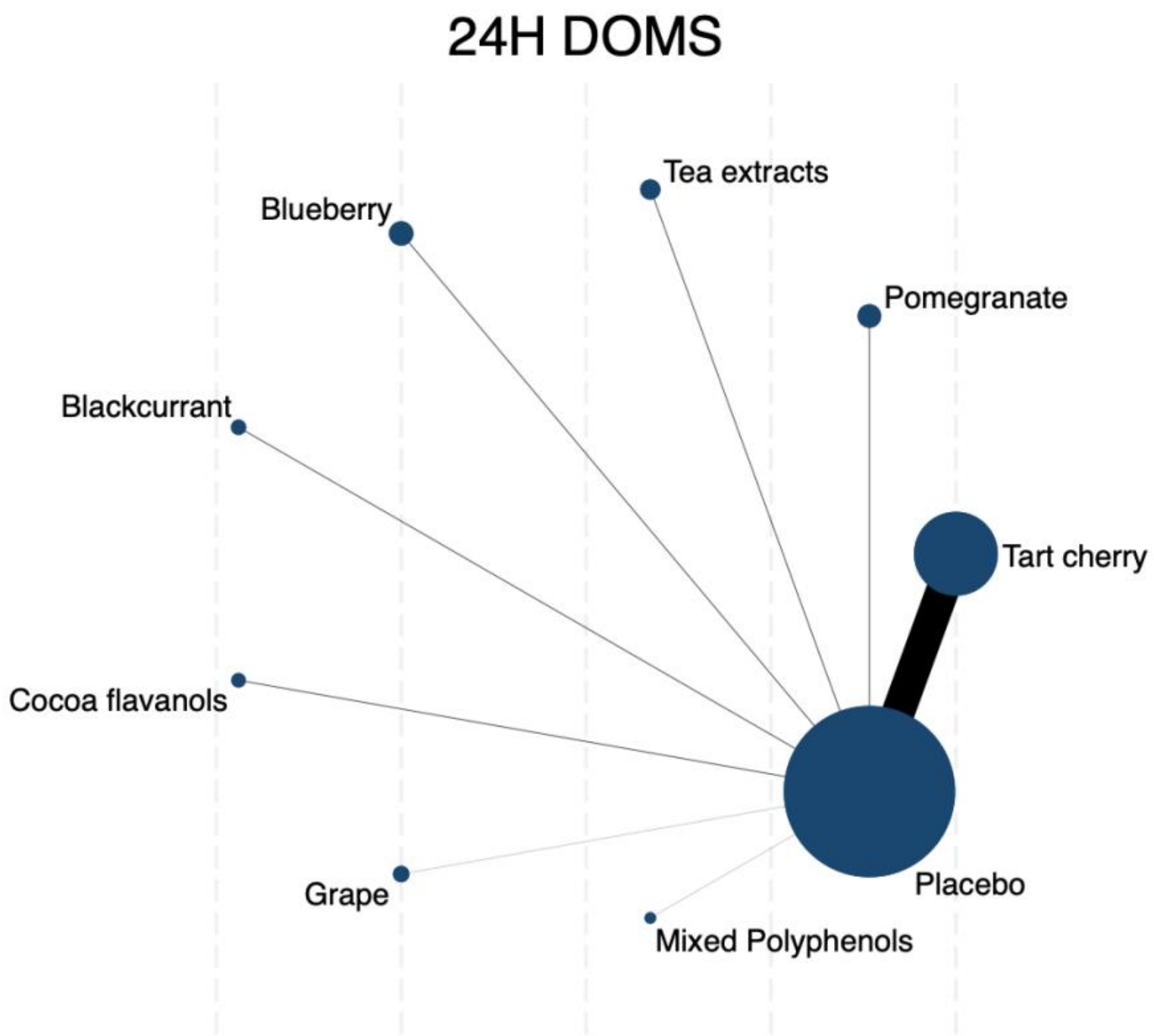

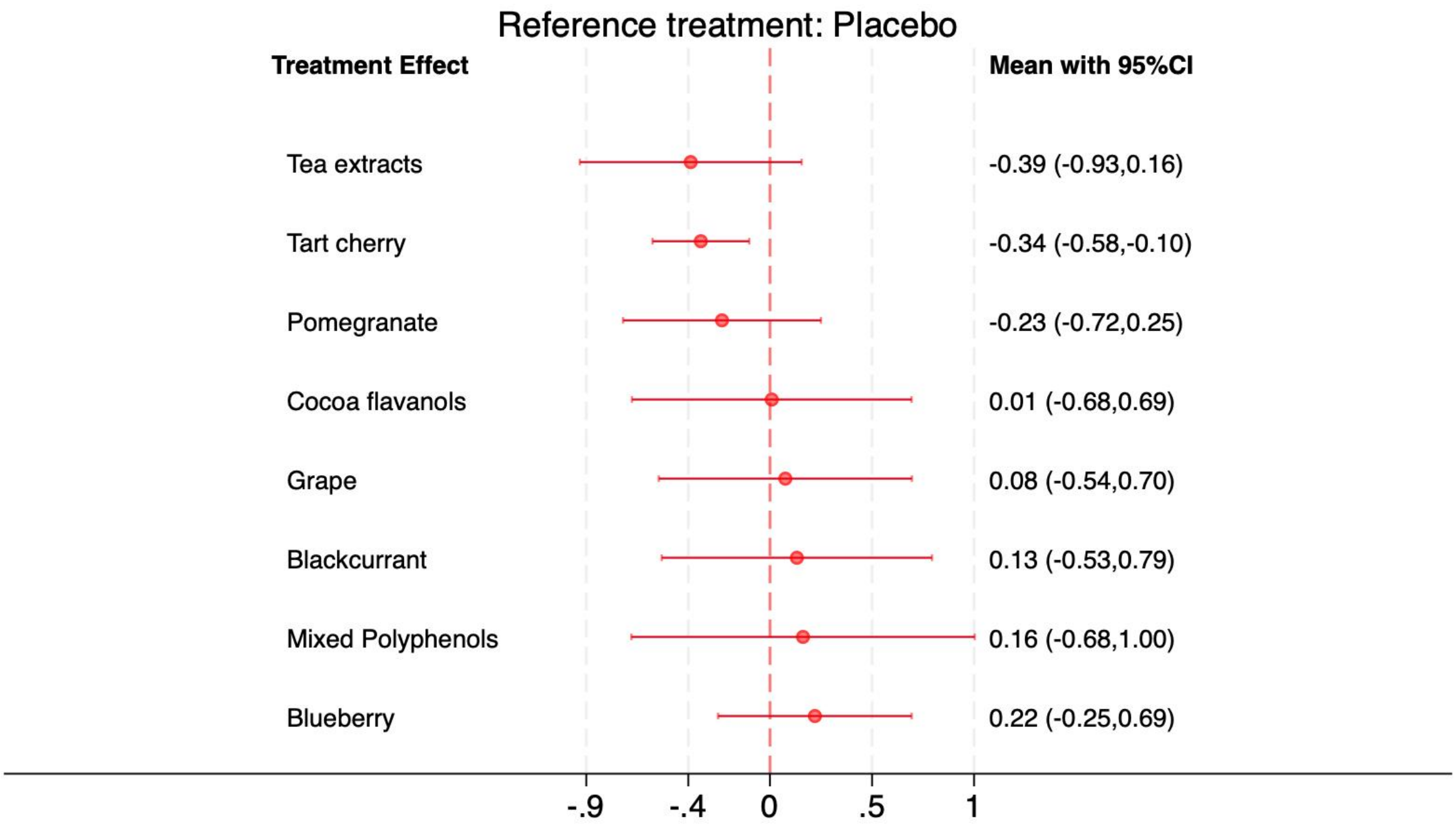

Figure S8.2: Network plot and forest plot for the 24-h pooled strength/torque outcome, based on the prespecified hierarchy of MVIC, isometric peak torque, isokinetic peak torque, and 1RM. Node size is proportional to the number of participants, and edge thickness is proportional to the number of studies for each comparison. The placebo node pools all eligible control conditions, including matched placebo and vehicle-matched controls. Positive SMD values favour plant-derived supplements.

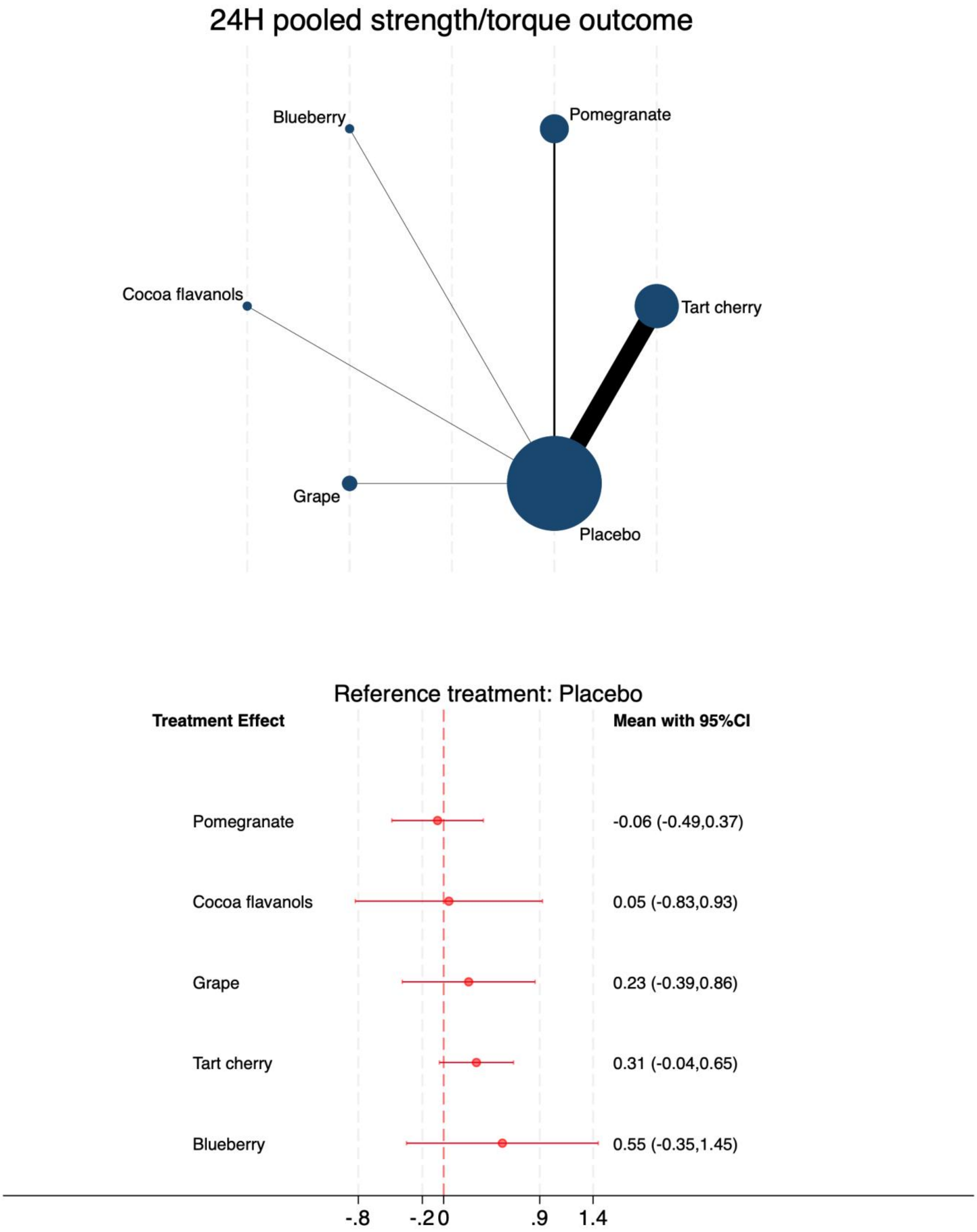

Figure S8.3: Network plot and forest plot for 24-h CK outcome. Node size is proportional to the number of participants, and edge thickness is proportional to the number of studies informing each comparison. The placebo node pools all eligible control conditions, including matched placebo and vehicle-matched controls. Negative SMD values favour plant-derived supplements.

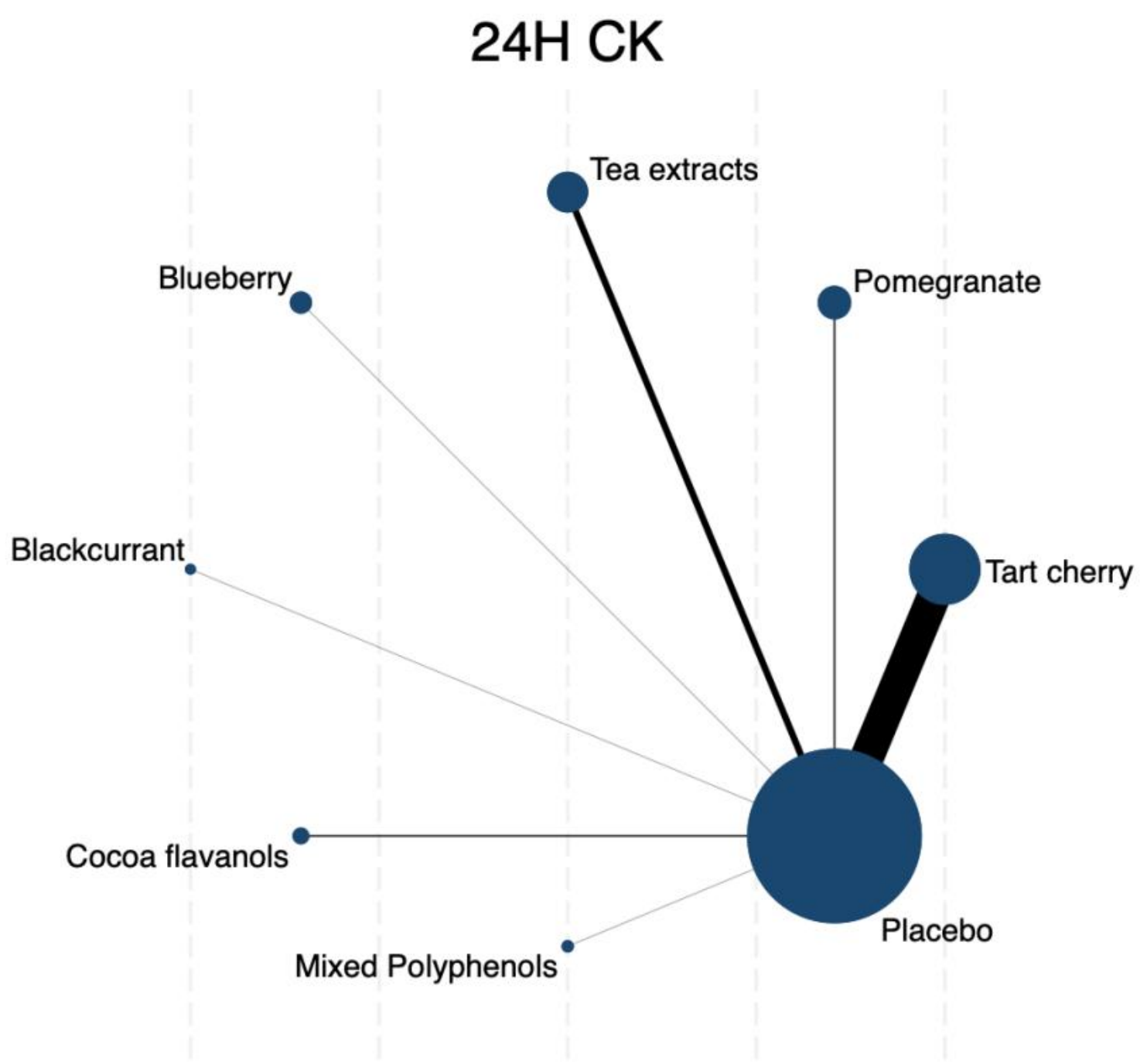

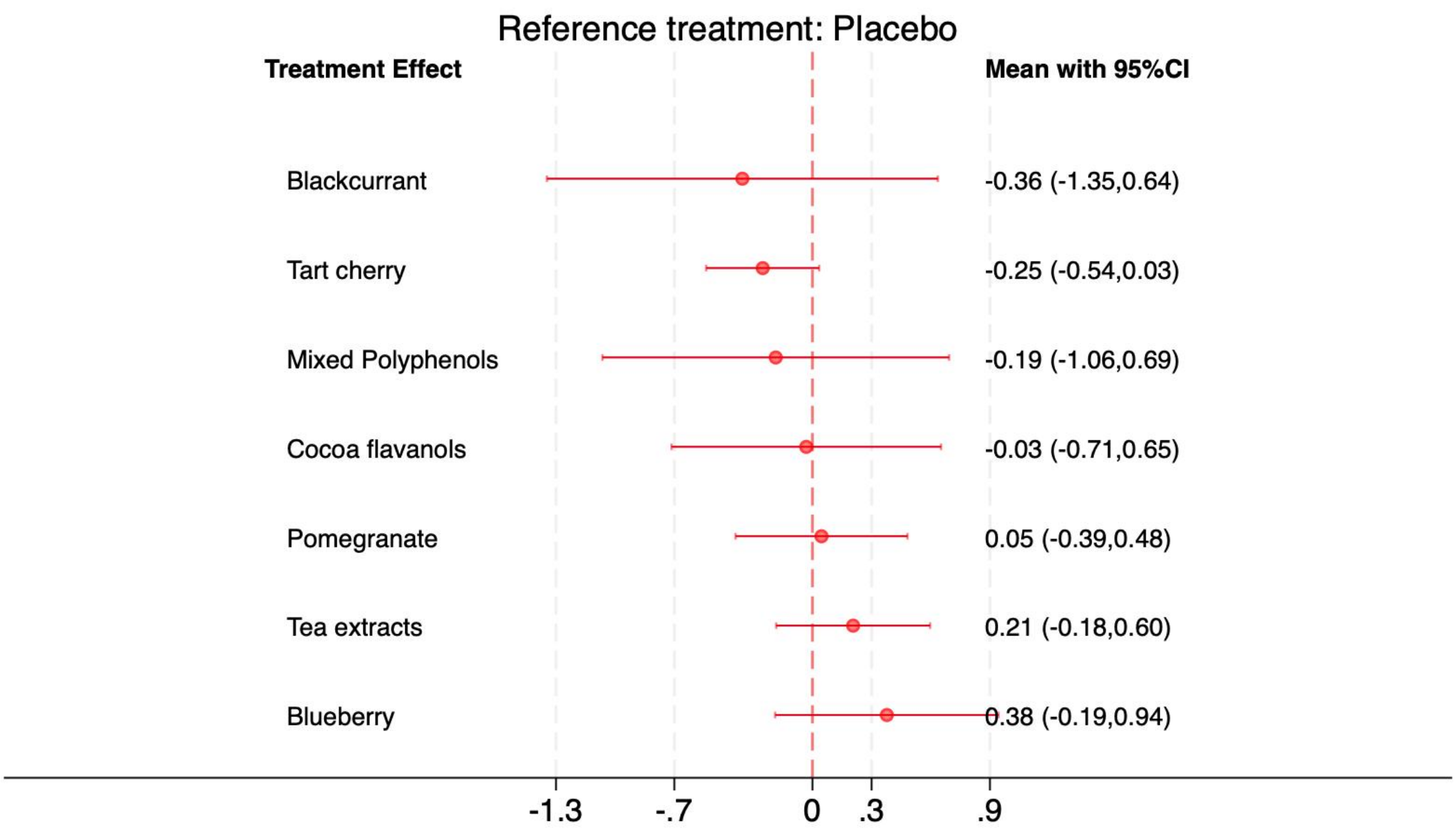

Figure S8.4: Network plot and forest plot for the 24-h CRP outcome. Node size is proportional to the number of participants, and edge thickness is proportional to the number of studies for each comparison. The placebo node pools all eligible control conditions, including matched placebo and vehicle-matched controls. Negative SMD values favour plant-derived supplements.

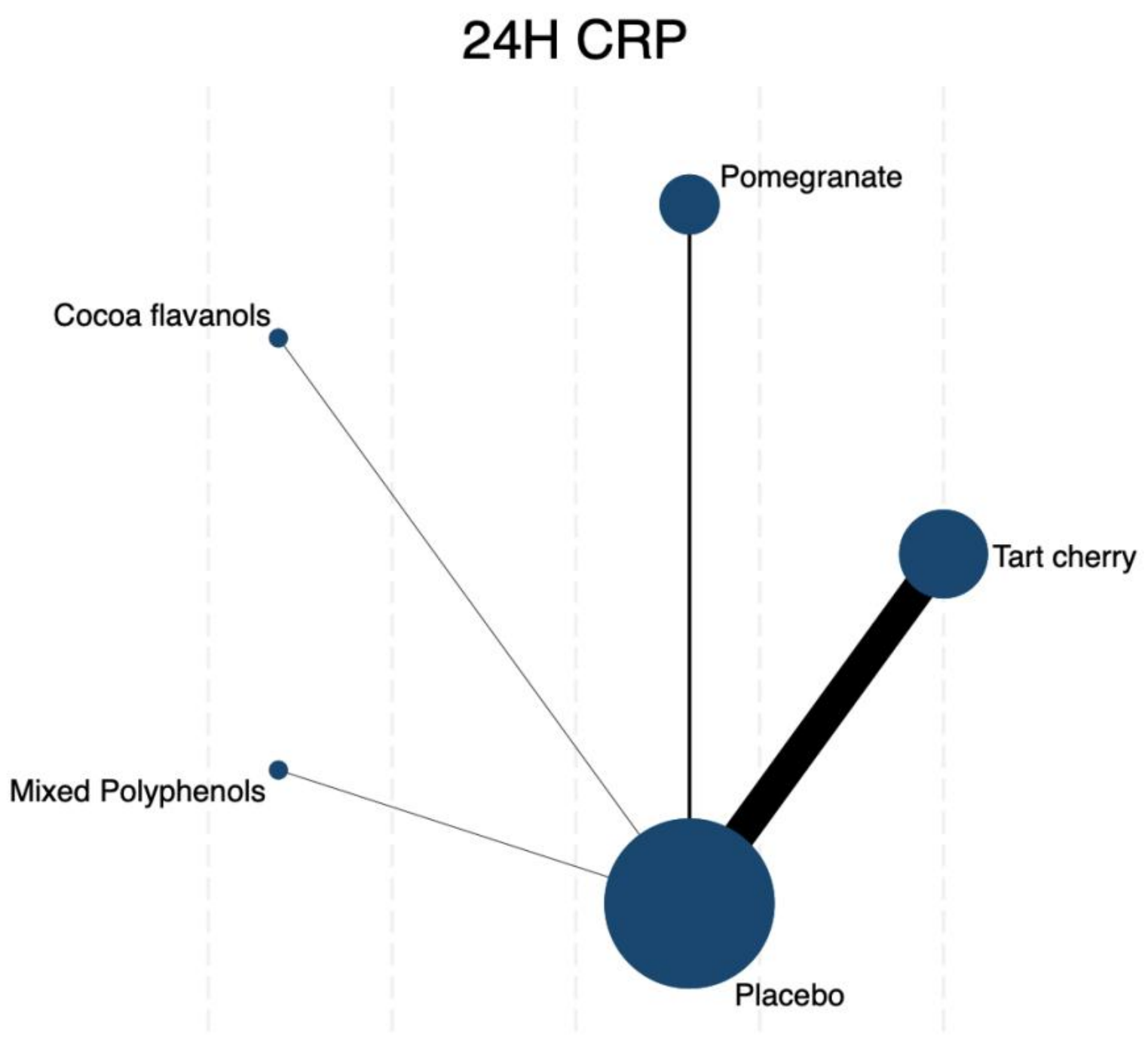

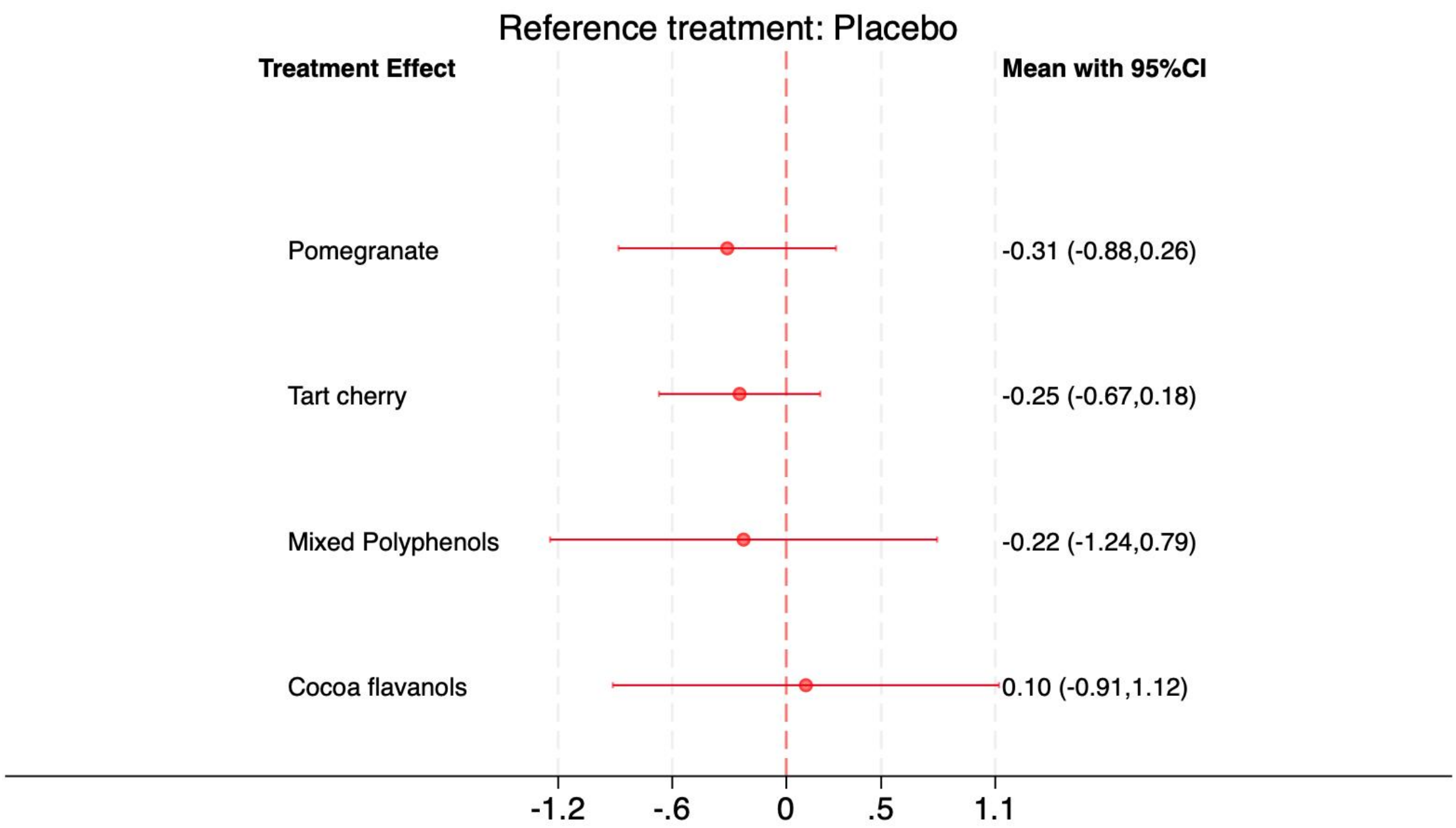

Figure S8.5: Network plot and forest plot for 72-h DOMS. Node size is proportional to the number of participants, and edge thickness is proportional to the number of studies for each comparison. Negative SMD values favour plant-derived supplements.

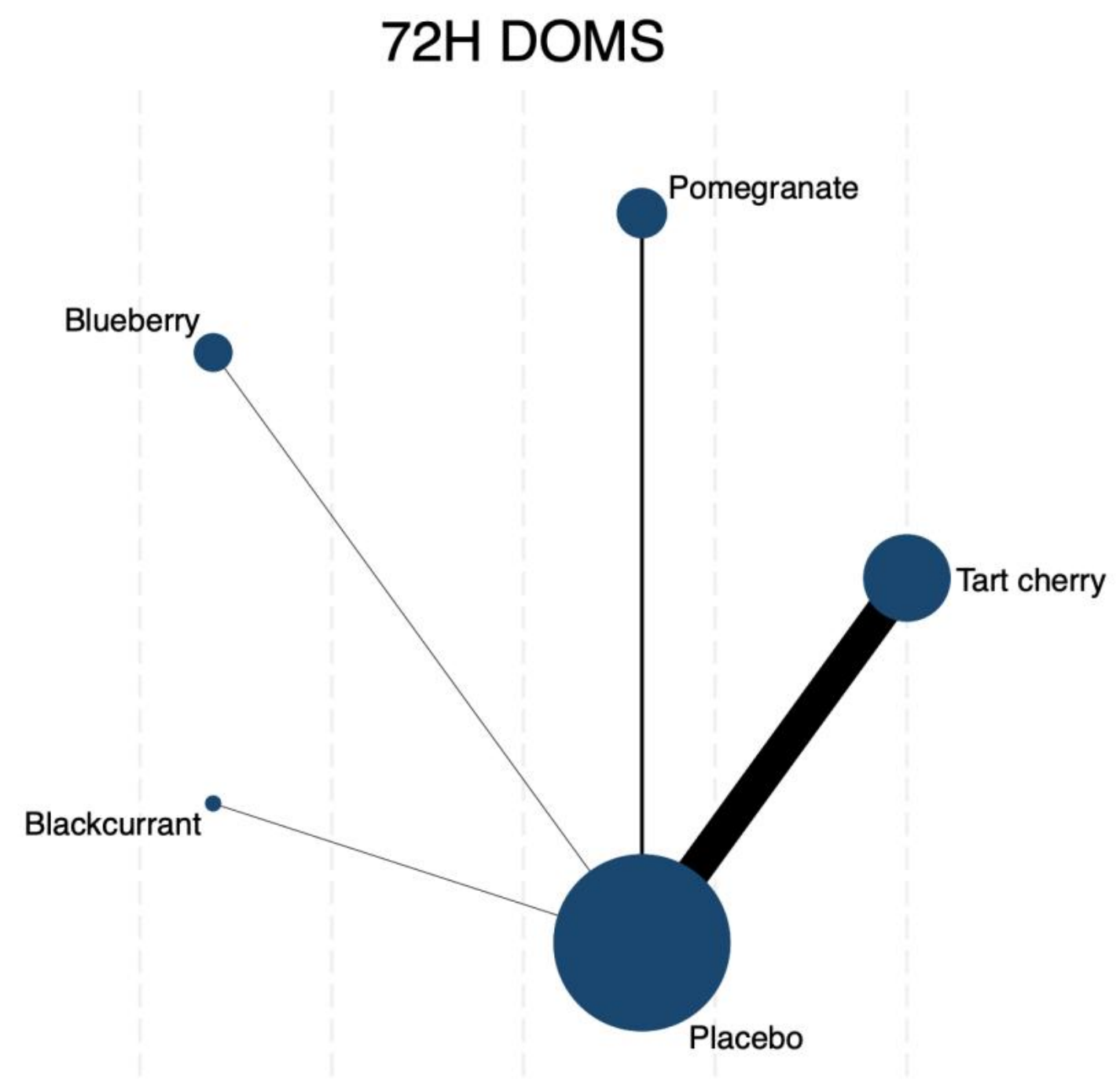

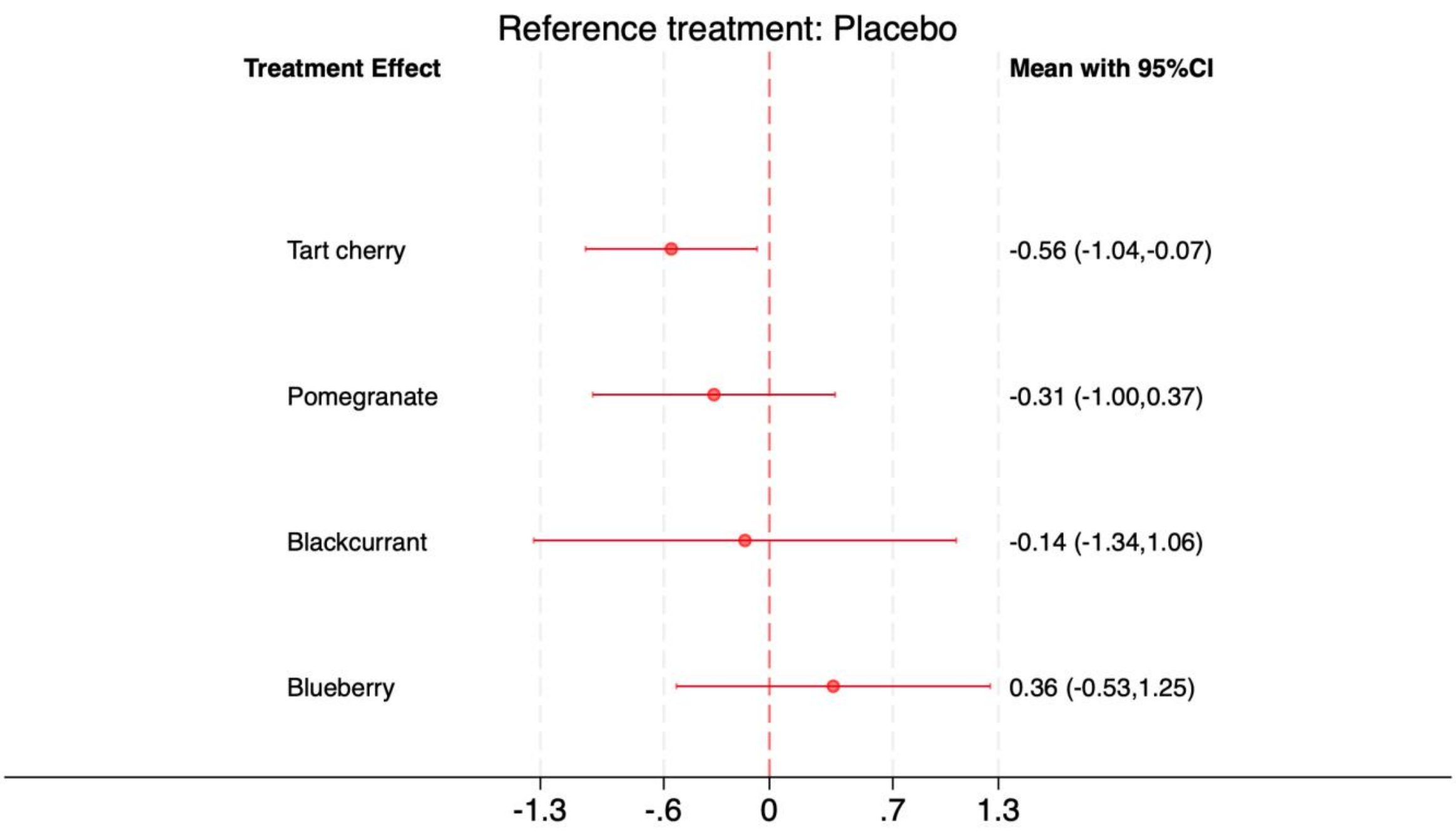

Figure S8.6: Network plot and forest plot for the 72-h pooled strength/torque outcome, based on the prespecified hierarchy of MVIC, isometric peak torque, isokinetic peak torque, and 1RM. Node size is proportional to the number of participants, and edge thickness is proportional to the number of studies for each comparison. The placebo node pools all eligible control conditions, including matched placebo and vehicle-matched controls. Positive SMD values favour plant-derived supplements.

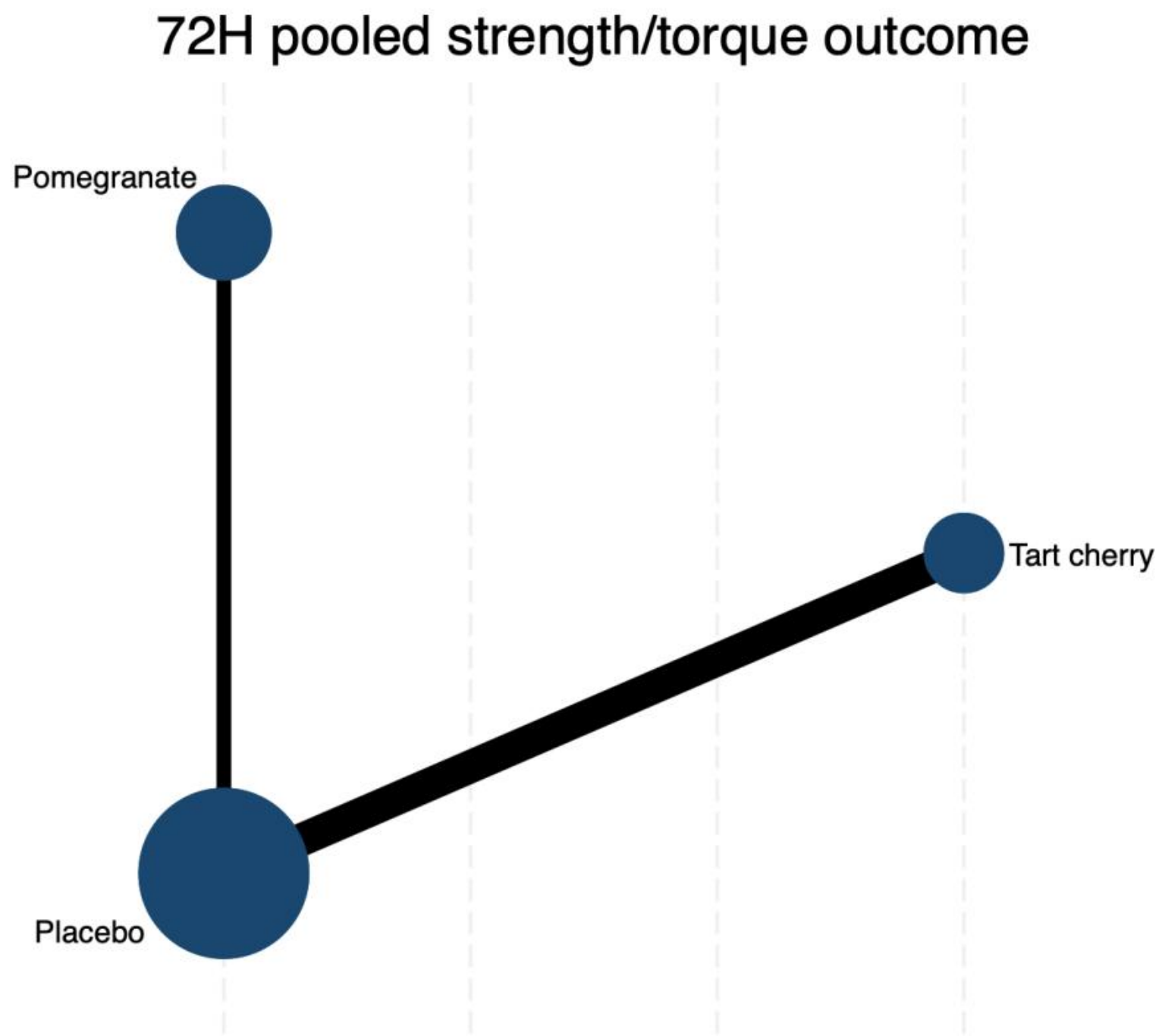

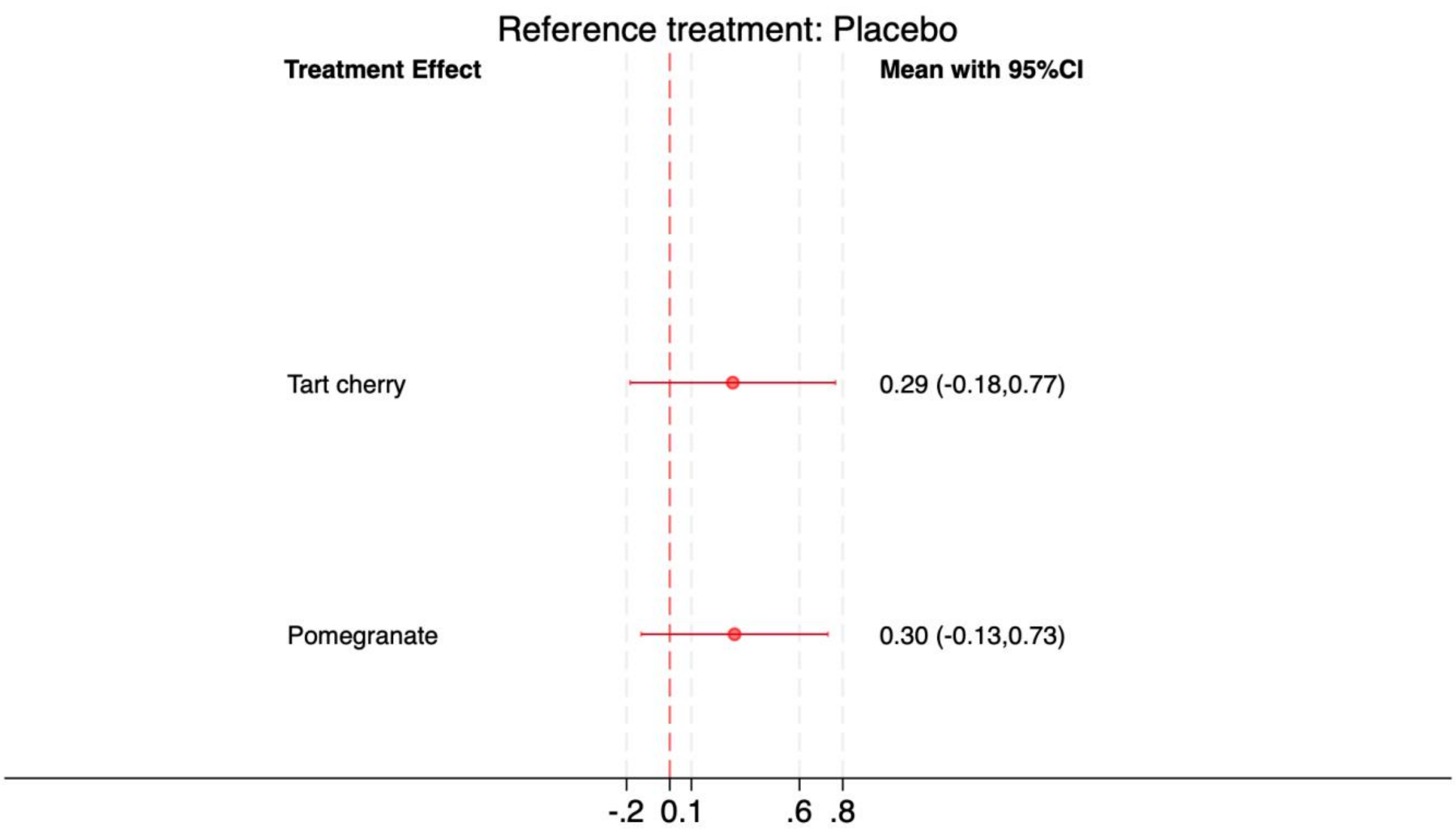

Figure S8.7: Network plot and forest plot for 72-h CK outcome. Node size is proportional to the number of participants, and edge thickness is proportional to the number of studies informing each comparison. Negative SMD values favour plant-derived supplements.

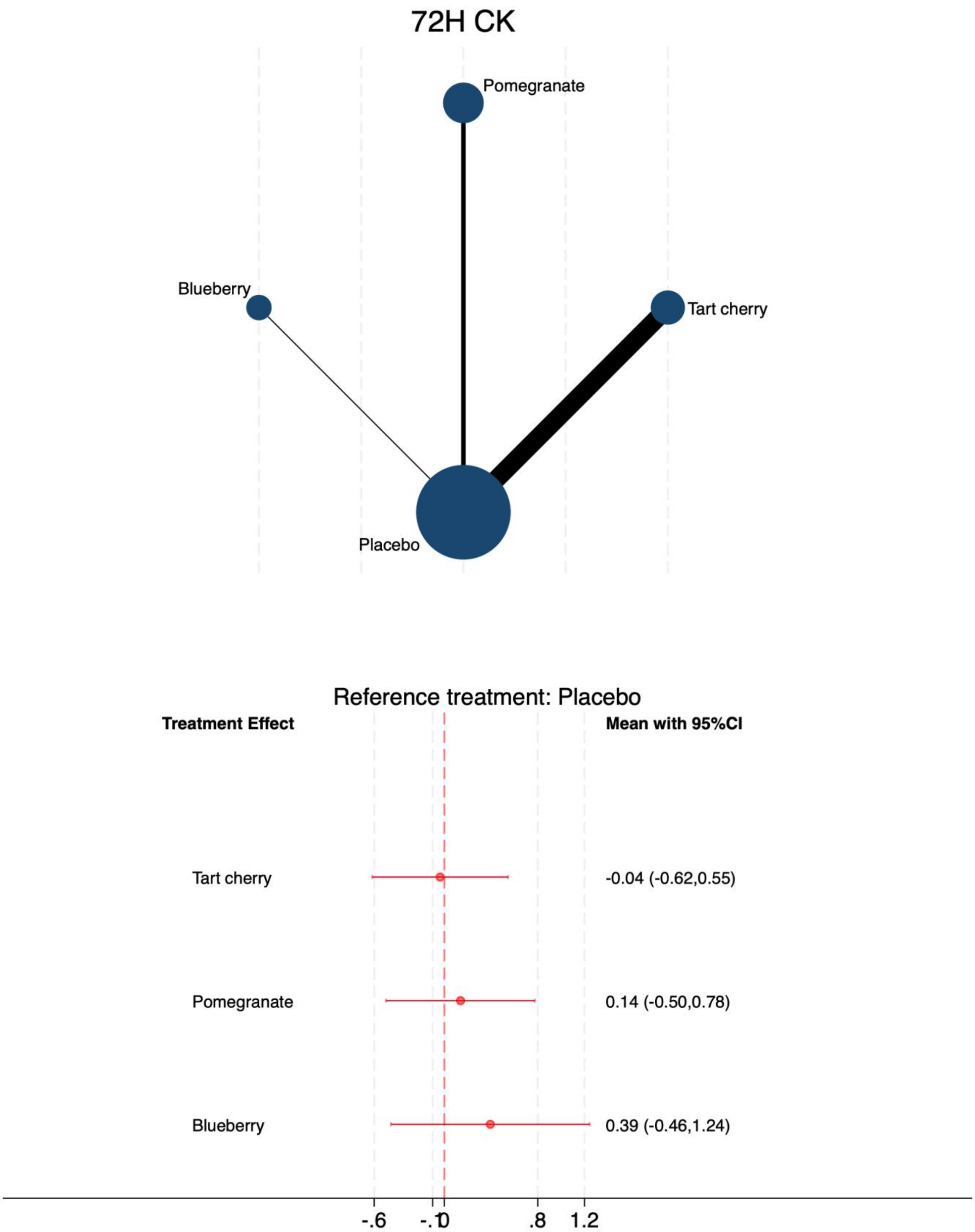

Figure S8.8: Network plot and forest plot for the 72-h CRP outcome. Node size is proportional to the number of participants, and edge thickness is proportional to the number of studies for each comparison. The placebo node pools all eligible control conditions, including matched placebo and vehicle-matched controls. Negative SMD values favour plant-derived supplements.

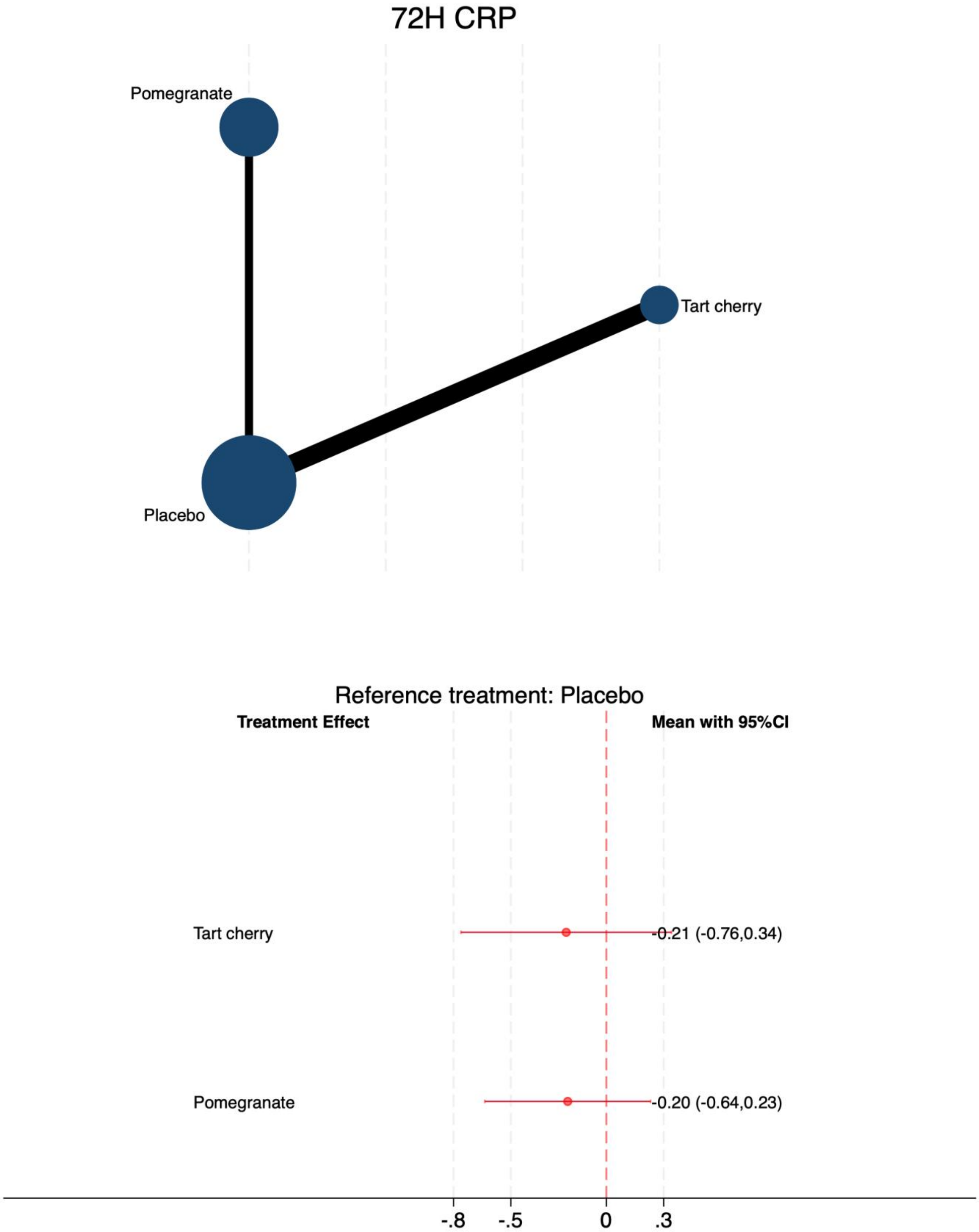

Appendix 9: SUCRA and cumulative probability plots

Figure S9.1: Cumulative ranking curves for interventions on 48-h DOMS in the network meta-analysis. A larger surface under the curve indicates a higher probability of a more favorable ranking, corresponding to lower 48-h DOMS values. The placebo node pools all eligible control conditions, including matched placebo and vehicle-matched controls.

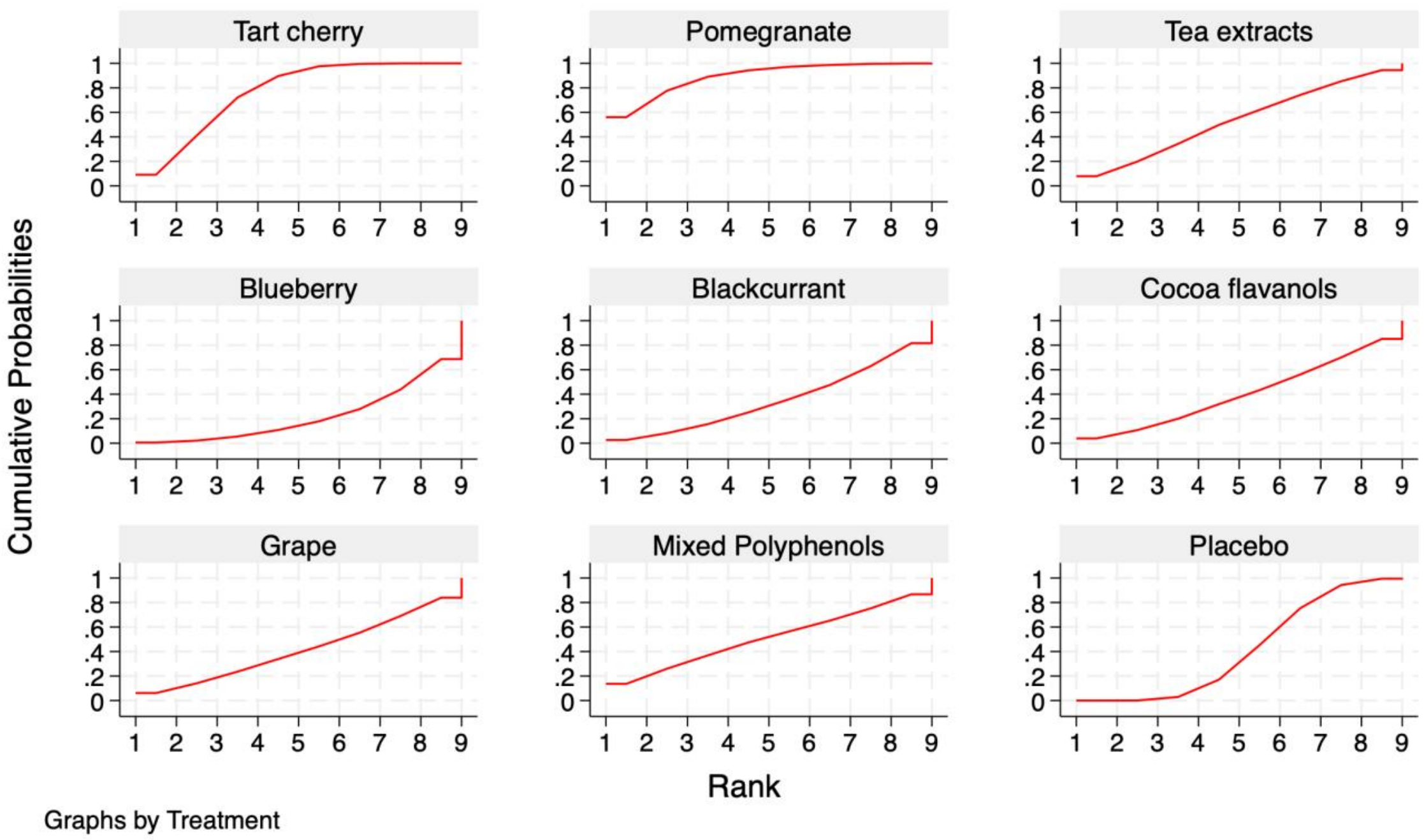

Table S9.1: SUCRA of the effects of the included interventions on 48-h DOMS.

Treatment	SUCRA	PrBest	MeanRank
Tart cherry	76.0	8.4	2.9
Pomegranate	89.3	56.9	1.9
Tea extracts	54.4	8.1	4.6
Blueberry	21.3	0.5	7.3
Blackcurrant	34.6	2.6	6.2
Cocoa flavanols	40.1	4.0	5.8
Grape	41.1	5.9	5.7
Mixed Polyphenols	51.2	13.7	4.9
Placebo	41.9	0	5.6

Abbreviations: SUCRA, surface under the cumulative ranking curve.

Figure S9.2: Cumulative ranking curves for interventions on the 48-h pooled strength/torque outcome in the network meta-analysis. A larger surface under the curve indicates a higher probability of a more favorable ranking, corresponding to greater strength/torque values. The placebo node pools all eligible control conditions, including matched placebo and vehicle-matched controls.

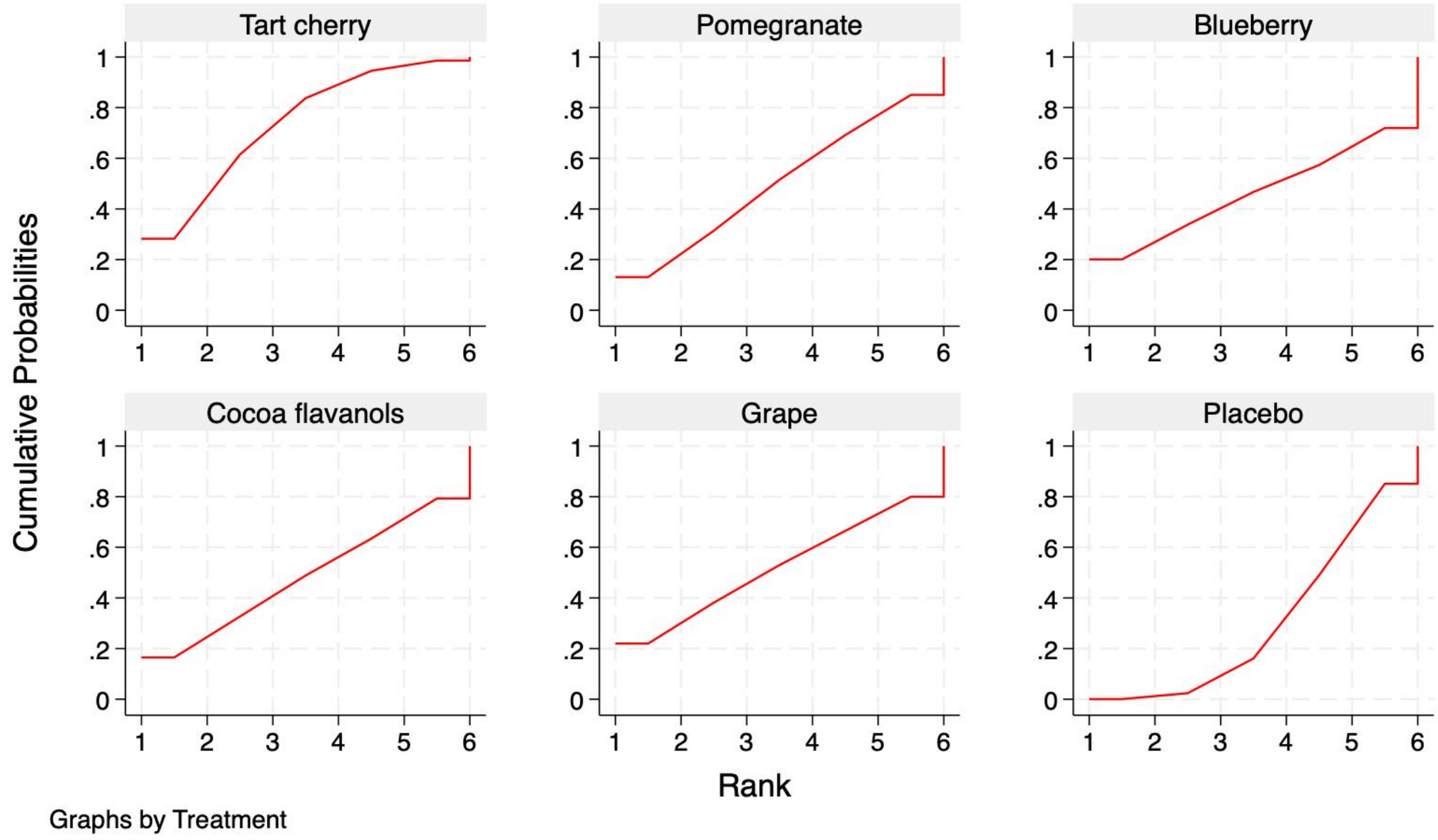

Table S9.2: SUCRA values for the effects of the included interventions on the 48-h pooled strength/torque outcome.

Treatment	SUCRA	PrBest	MeanRank
Tart cherry	73.3	28.2	2.3
Pomegranate	50.1	13.1	3.5
Blueberry	46	20.1	3.7
Cocoa flavanols	48.2	16.5	3.6
Grape	51.9	22	3.4
Placebo	30.5	0.1	4.5

Abbreviations: SUCRA, surface under the cumulative ranking curve.

Figure S9.3: Cumulative ranking curves for interventions on the 48-h CK outcome in the network meta-analysis. As lower CK values indicate a more favorable outcome, a larger surface under the curve reflects a higher probability of ranking among the best interventions for reducing 48-h CK. The placebo node pools all eligible control conditions, including matched placebo and vehicle-matched controls.

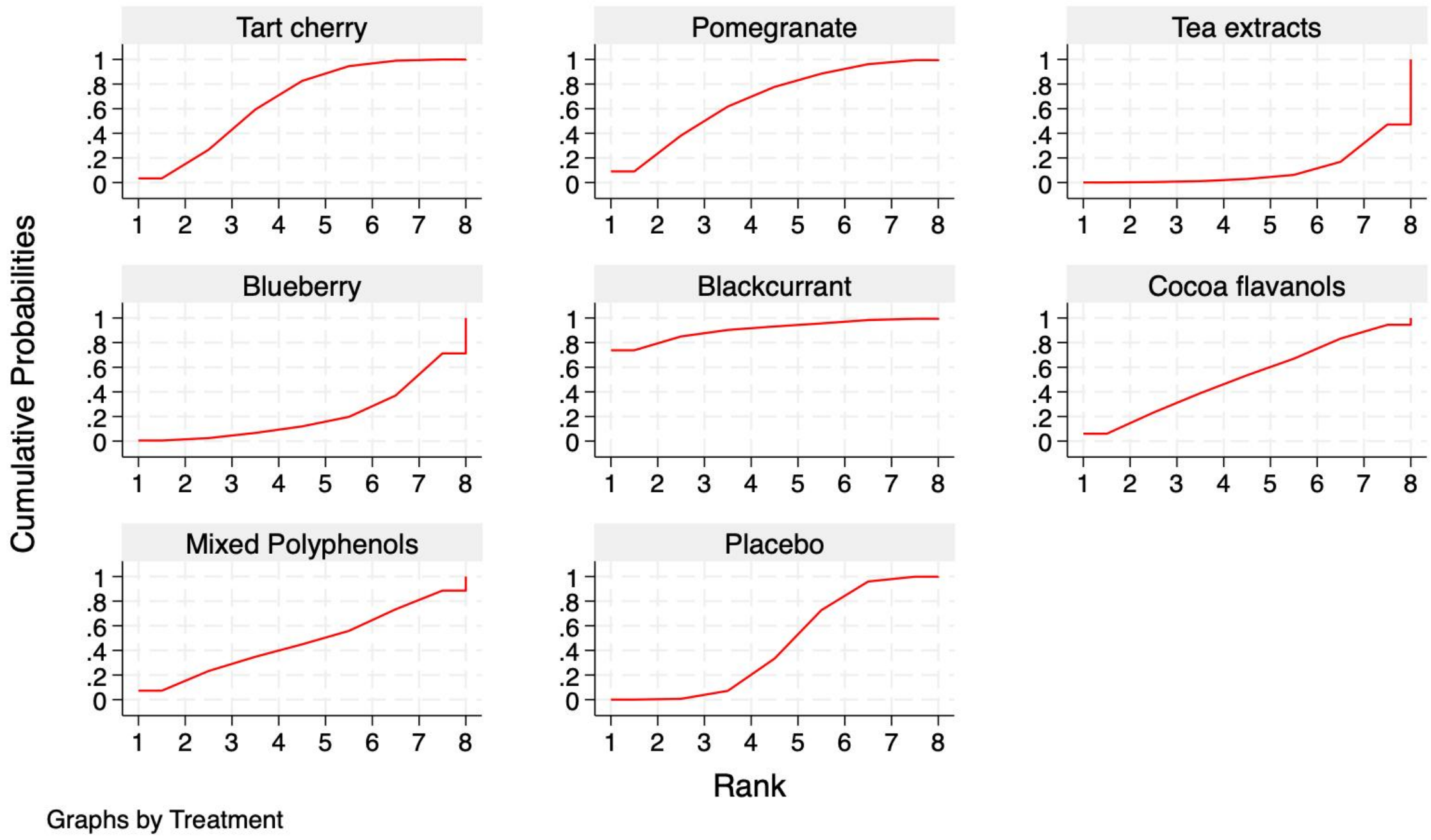

Table S9.3: SUCRA values for the effects of the included interventions on the 48-h CK outcome.

Treatment	SUCRA	PrBest	MeanRank
Tart cherry	66.5	3.4	3.3
Pomegranate	67.2	9	3.3
Tea extracts	10.7	0.1	7.3
Blueberry	21.4	0.5	6.5
Blackcurrant	90.8	73.8	1.6
Cocoa flavanols	52.3	6	4.3
Mixed Polyphenols	46.9	7.3	4.7
Placebo	44.2	0	4.9

Abbreviations: SUCRA, surface under the cumulative ranking curve.

Figure S9.4: Cumulative ranking curves for interventions on the CRP outcome in the network meta-analysis. As lower CRP values indicate a more favorable outcome, a larger surface under the curve reflects a higher probability of ranking among the best interventions for reducing 48-h CRP. The placebo node pools all eligible control conditions, including matched placebo and vehicle-matched controls.

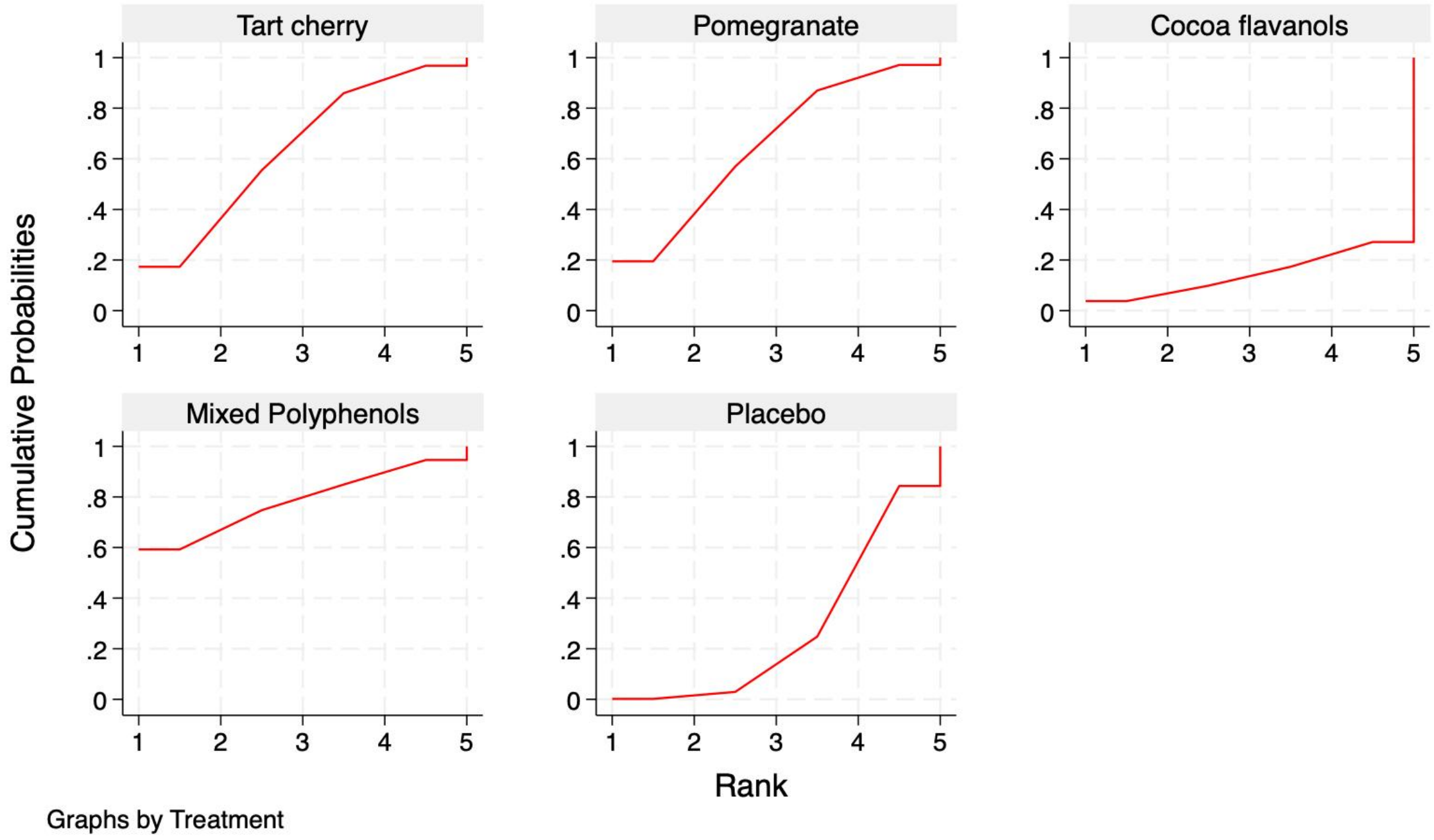

Table S9.4: SUCRA values for the effects of the included interventions on the CRP.

Treatment	SUCRA	PrBest	MeanRank
Tart cherry	63.9	17.3	2.4
Pomegranate	65.1	19.5	2.4
Cocoa flavanols	14.5	3.8	4.4
Mixed Polyphenols	78.4	59.2	1.9
Placebo	28.1	0.2	3.9

Abbreviations: SUCRA, surface under the cumulative ranking curve

Figure S9.5: Cumulative ranking curves for interventions on 24-h DOMS in the network meta -analysis. A larger surface under the curve indicates a higher probability of a more favorable ranking, corresponding to lower 24-h DOMS values. The placebo node pools all eligible control conditions, including matched placebo and vehicle-matched controls.

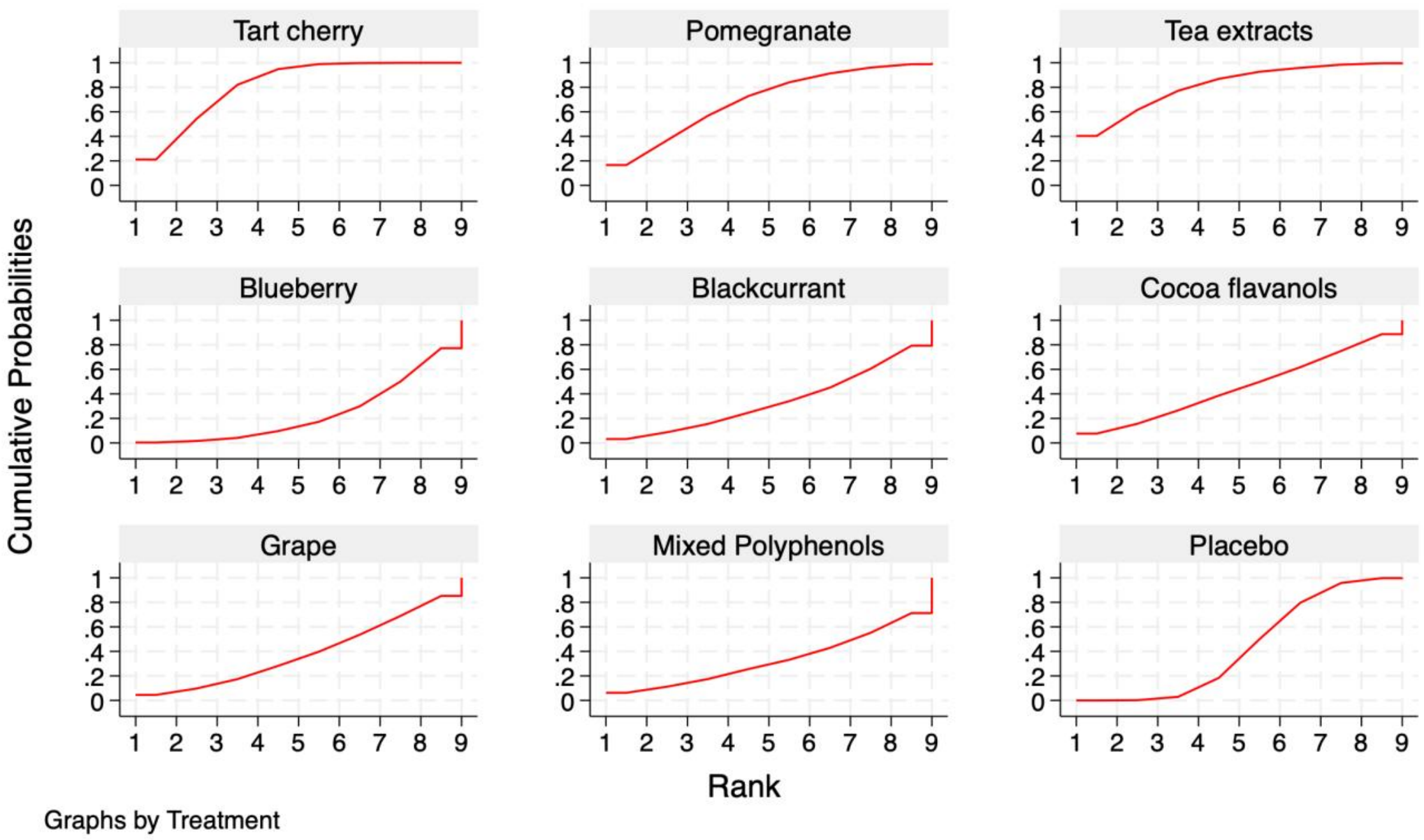

Table S9.5: SUCRA of the effects of the included interventions on 24-h DOMS.

Treatment	SUCRA	PrBest	MeanRank
Tart cherry	81.7	20.9	2.5
Pomegranate	69	16.9	3.5
Tea extracts	81	40.8	2.5
Blueberry	24.2	0.5	7.1
Blackcurrant	33.7	3.3	6.3
Cocoa flavanols	44.1	7	5.5
Grape	39.5	4	5.8
Mixed Polyphenols	33.2	6.6	6.3
Placebo	43.6	0	5.5

Abbreviations: SUCRA, surface under the cumulative ranking curve.

Figure S9.6: Cumulative ranking curves for interventions on the 24-h pooled strength/torque outcome in the network meta-analysis. A larger surface under the curve indicates a higher probability of a more favorable ranking, corresponding to greater strength/torque values. The placebo node pools all eligible control conditions, including matched placebo and vehicle-matched controls.

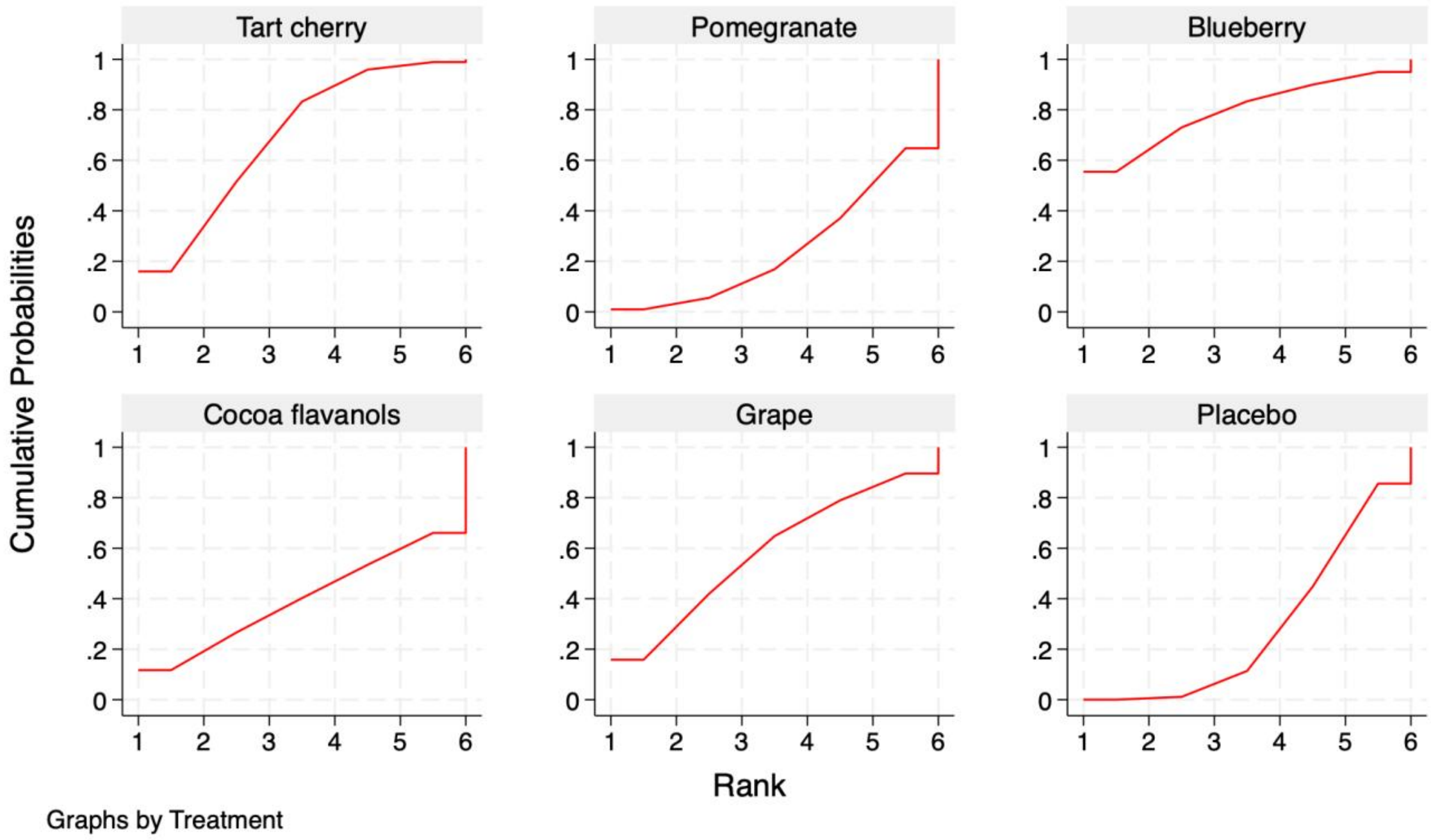

Table S9.6: SUCRA values for the effects of the included interventions on the 24-h pooled strength/torque outcome.

Treatment	SUCRA	PrBest	MeanRank
Tart cherry	69.1	16	2.5
Pomegranate	25	1	4.7
Blueberry	79.4	55.5	2
Cocoa flavanols	39.6	11.7	4
Grape	58.2	15.8	3.1
Placebo	28.6	0	4.6

Abbreviations: SUCRA, surface under the cumulative ranking curve.

Figure S9.7: Cumulative ranking curves for interventions on the 24-h CK outcome in the network meta-analysis. As lower CK values indicate a more favorable outcome, a larger surface under the curve reflects a higher probability of ranking among the best interventions for reducing 24-h CK. The placebo node pools all eligible control conditions, including matched placebo and vehicle-matched controls.

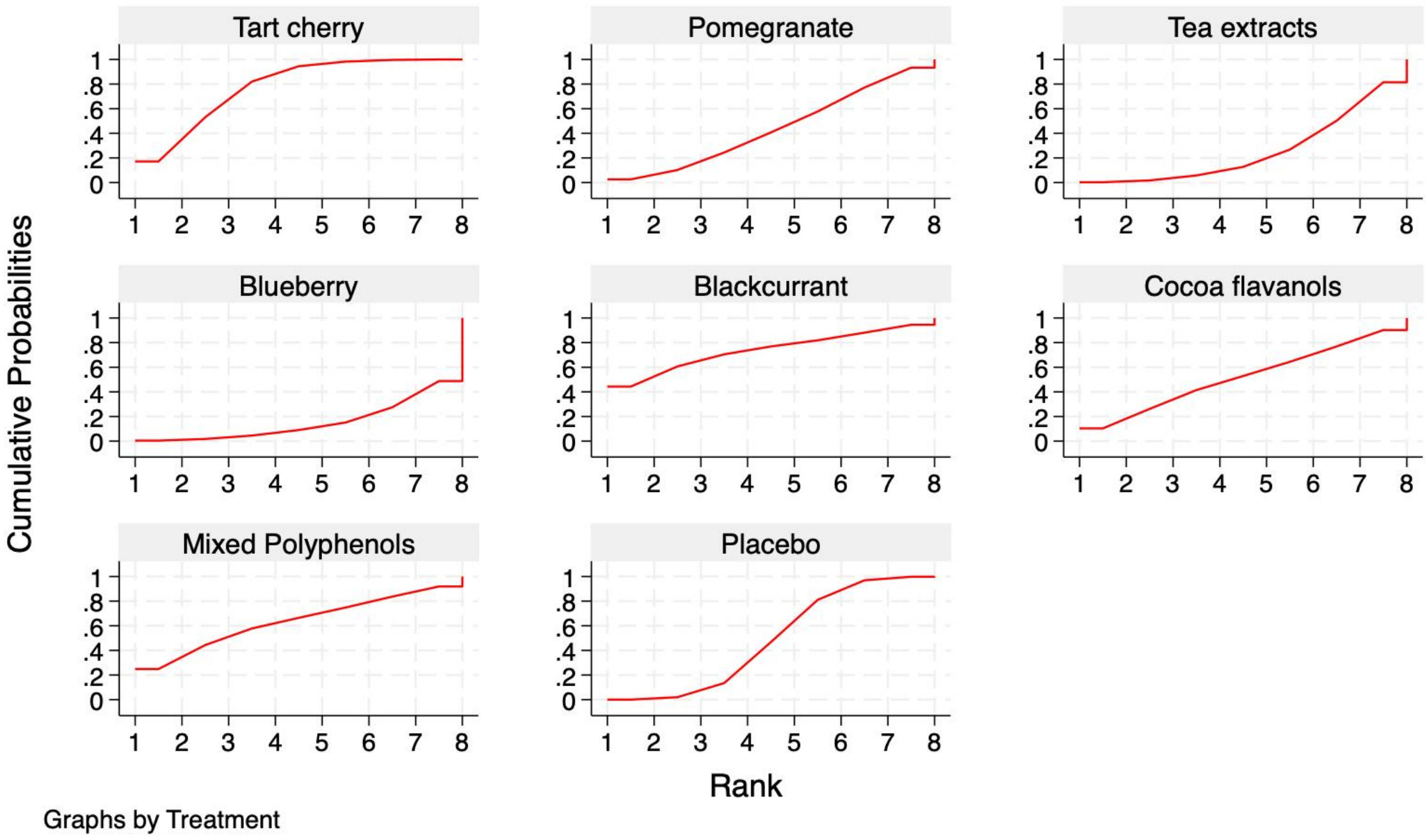

Table S9.7: SUCRA values for the effects of the included interventions on the 24-h CK outcome.

Treatment	SUCRA	PrBest	MeanRank
Tart cherry	77.8	17.2	2.6
Pomegranate	43.7	2.6	4.9
Tea extracts	25.6	0.3	6.2
Blueberry	15.3	0.4	6.9
Blackcurrant	73.8	44.3	2.8
Cocoa flavanols	51.8	10.4	4.4
Mixed Polyphenols	63.4	24.9	3.6
Placebo	48.6	0	4.6

Abbreviations: SUCRA, surface under the cumulative ranking curve.

Figure S9.8: Cumulative ranking curves for interventions on the CRP outcome in the network meta-analysis. As lower CRP values indicate a more favorable outcome, a larger surface under the curve reflects a higher probability of ranking among the best interventions for reducing 24-h CRP. The placebo node pools all eligible control conditions, including matched placebo and vehicle-matched controls.

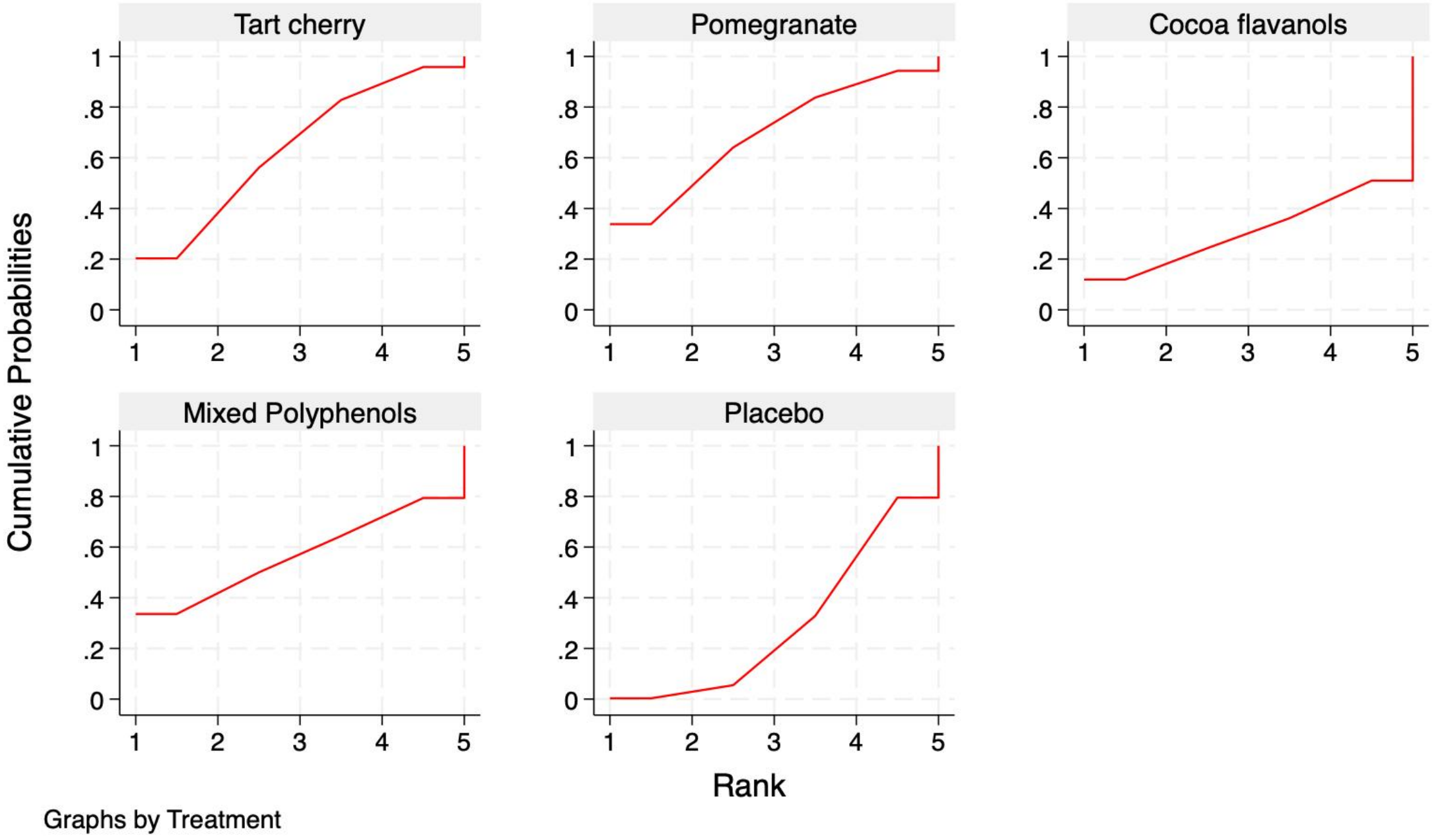

Table S9.8: SUCRA values for the effects of the included interventions on the CRP outcome.

Treatment	SUCRA	PrBest	MeanRank
Tart cherry	63.8	20.3	2.4
Pomegranate	69	33.8	2.2
Cocoa flavanols	30.8	12	3.8
Mixed Polyphenols	56.9	33.6	2.7
Placebo	29.5	0.3	3.8

Abbreviations: SUCRA, surface under the cumulative ranking curve

Figure S9.9: Cumulative ranking curves for interventions on 72-h DOMS in the network meta-analysis. A larger surface under the curve indicates a higher probability of a more favorable ranking, corresponding to lower 72-h DOMS values. The placebo node pools all eligible control conditions, including matched placebo and vehicle-matched controls.

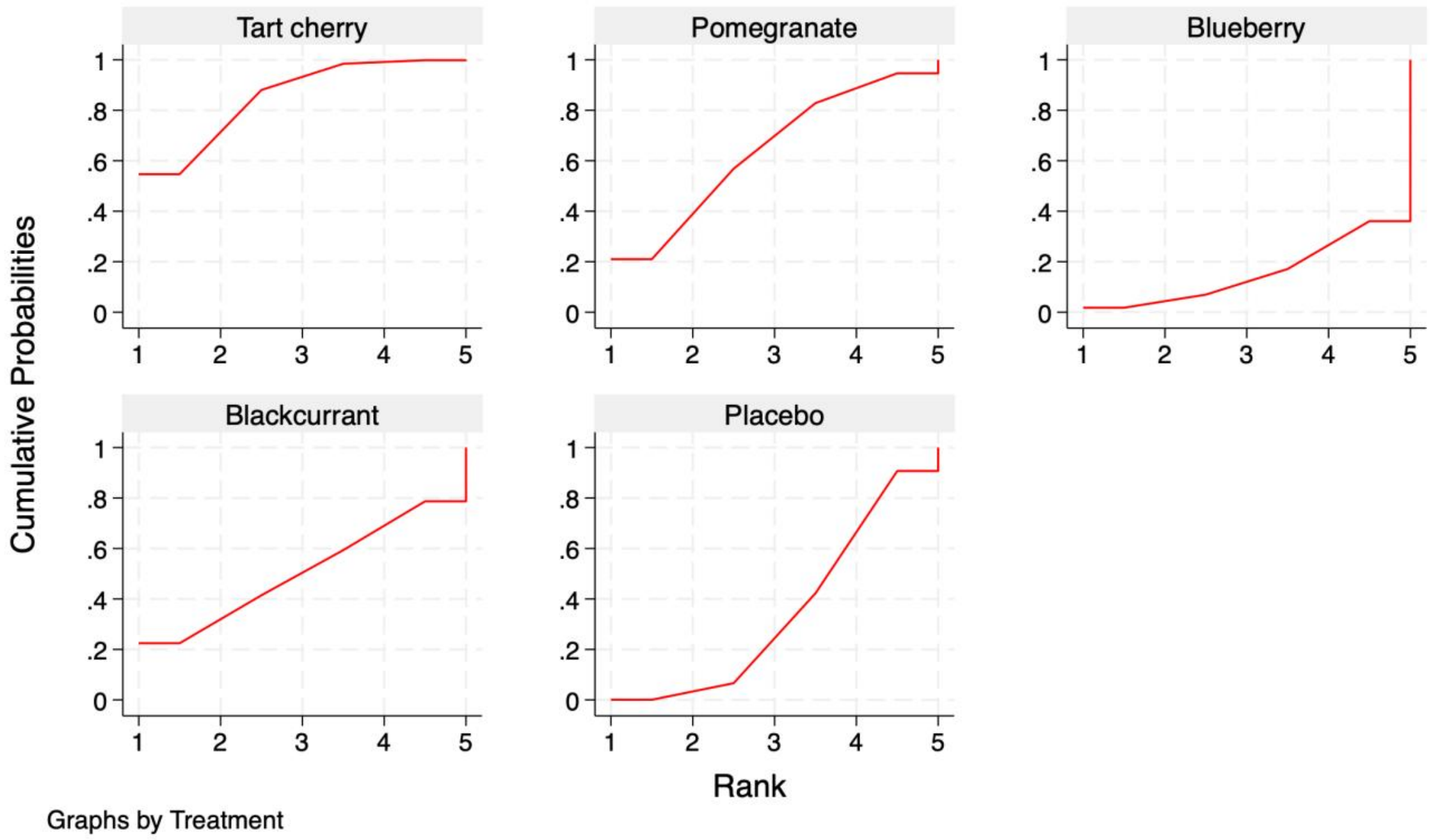

Table S9.9: SUCRA of the effects of the included interventions on 72-h DOMS.

Treatment	SUCRA	PrBest	MeanRank
Tart cherry	85.3	54.7	1.6
Pomegranate	63.8	21	2.4
Blueberry	15.5	1.8	4.4
Blackcurrant	50.5	22.5	3
Placebo	34.9	0.1	3.6

Abbreviations: SUCRA, surface under the cumulative ranking curve.

Figure S9.10: Cumulative ranking curves for interventions on the 72-h pooled strength/torque outcome in the network meta-analysis. A larger surface under the curve indicates a higher probability of a more favorable ranking, corresponding to greater strength/torque values. The placebo node pools all eligible control conditions, including matched placebo and vehicle-matched controls.

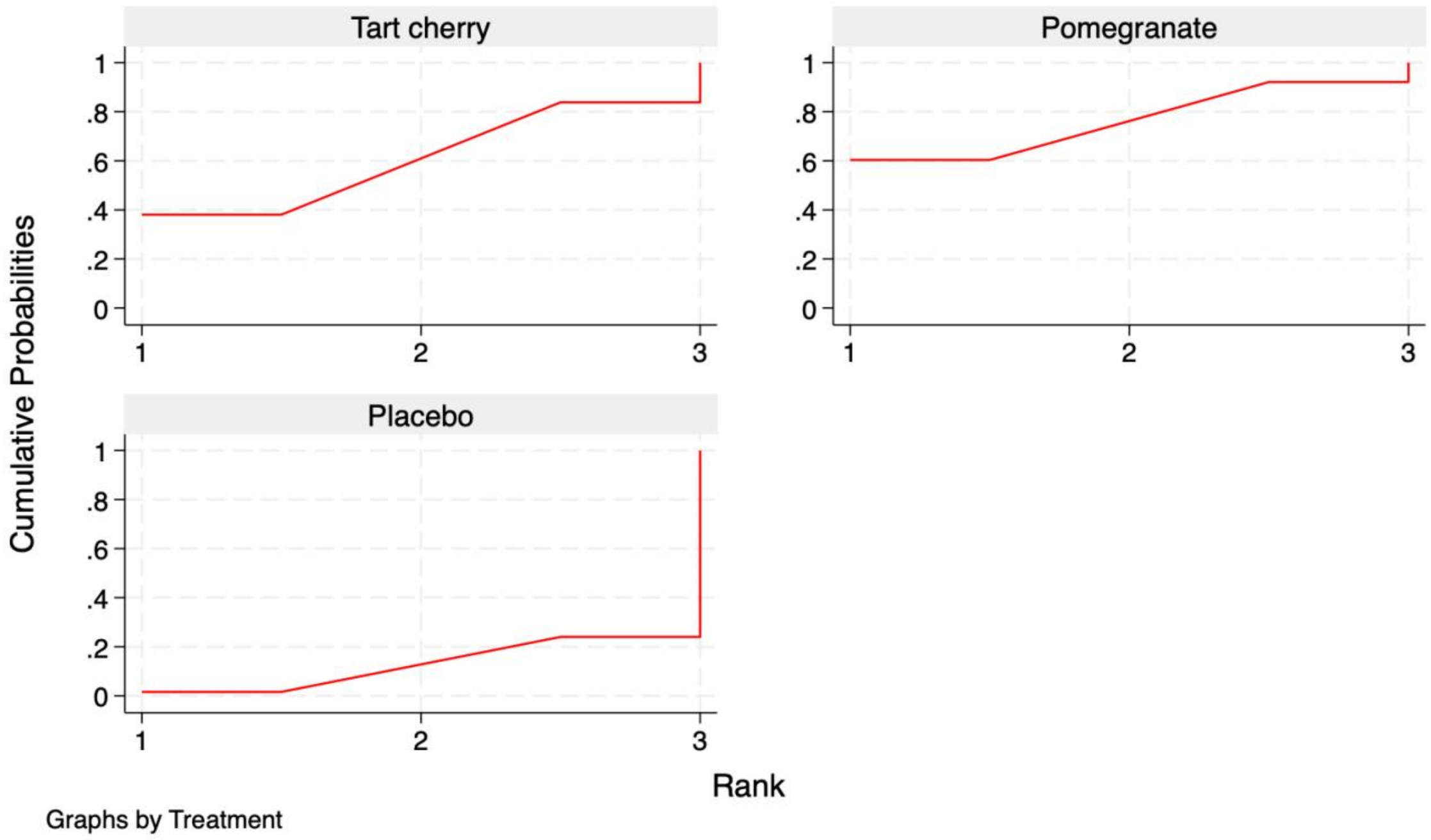

Table S9.10: SUCRA values for the effects of the included interventions on the 72-h pooled strength/torque outcome.

Treatment	SUCRA	PrBest	MeanRank
Tart cherry	68.7	48.5	1.6
Pomegranate	71.1	50.6	1.6
Placebo	10.2	0.9	2.8

Abbreviations: SUCRA, surface under the cumulative ranking curve.

Figure S9.11: Cumulative ranking curves for interventions on the 72-h CK outcome in the network meta-analysis. As lower CK values indicate a more favorable outcome, a larger surface under the curve reflects a higher probability of ranking among the best interventions for reducing 72-h CK. The placebo node pools all eligible control conditions, including matched placebo and vehicle-matched controls.

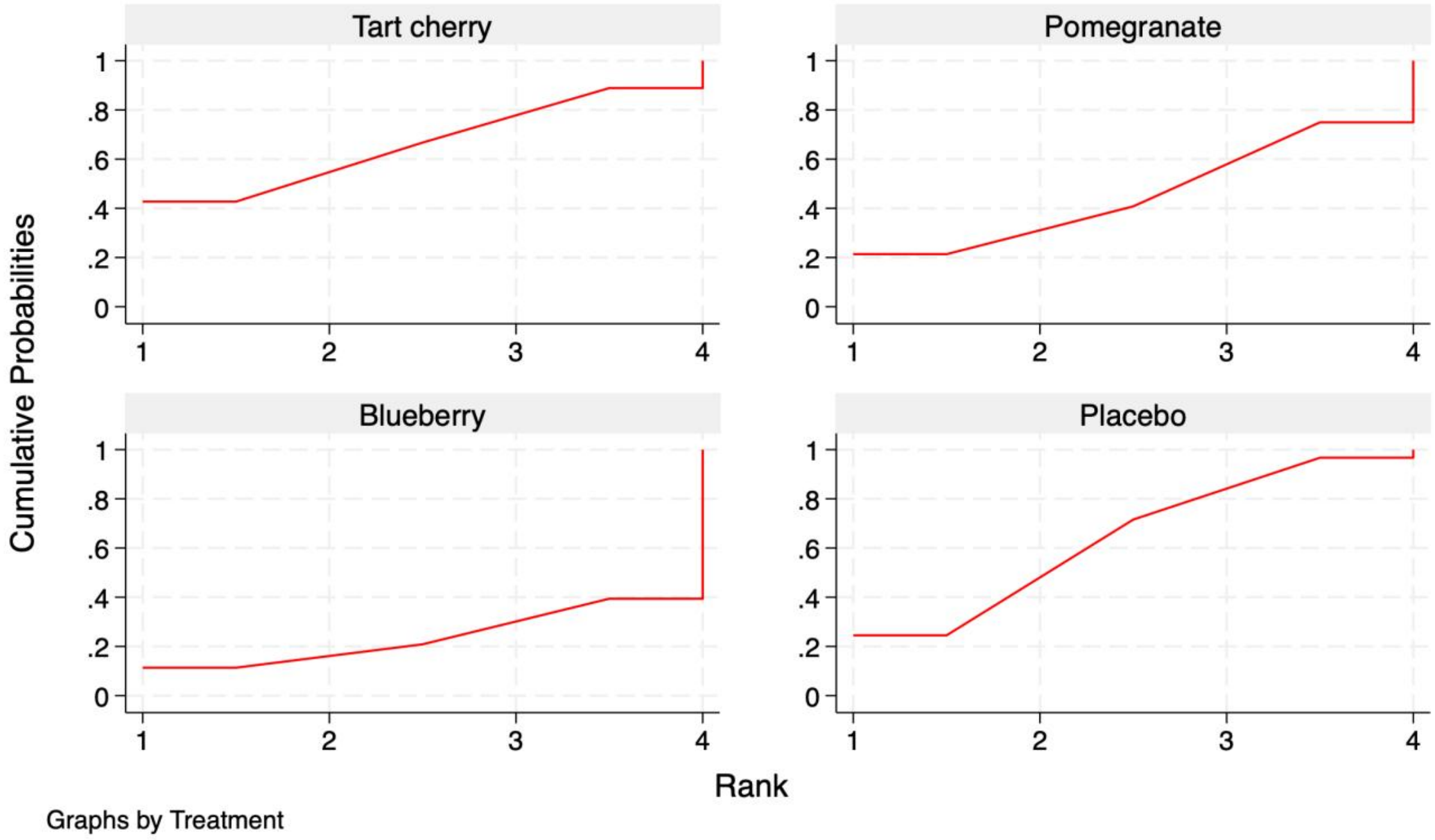

Table S9.11: SUCRA values for the effects of the included interventions on the 72-h CK outcome.

Treatment	SUCRA	PrBest	MeanRank
Tart cherry	66.1	42.7	2
Pomegranate	45.7	21.4	2.6
Blueberry	23.9	11.4	3.3
Placebo	64.3	24.5	2.1

Abbreviations: SUCRA, surface under the cumulative ranking curve.

Figure S9.12: Cumulative ranking curves for interventions on the CRP outcome in the network meta-analysis. As lower CRP values indicate a more favorable outcome, a larger surface under the curve reflects a higher probability of ranking among the best interventions for reducing 72-h CRP. The placebo node pools all eligible control conditions, including matched placebo and vehicle-matched controls.

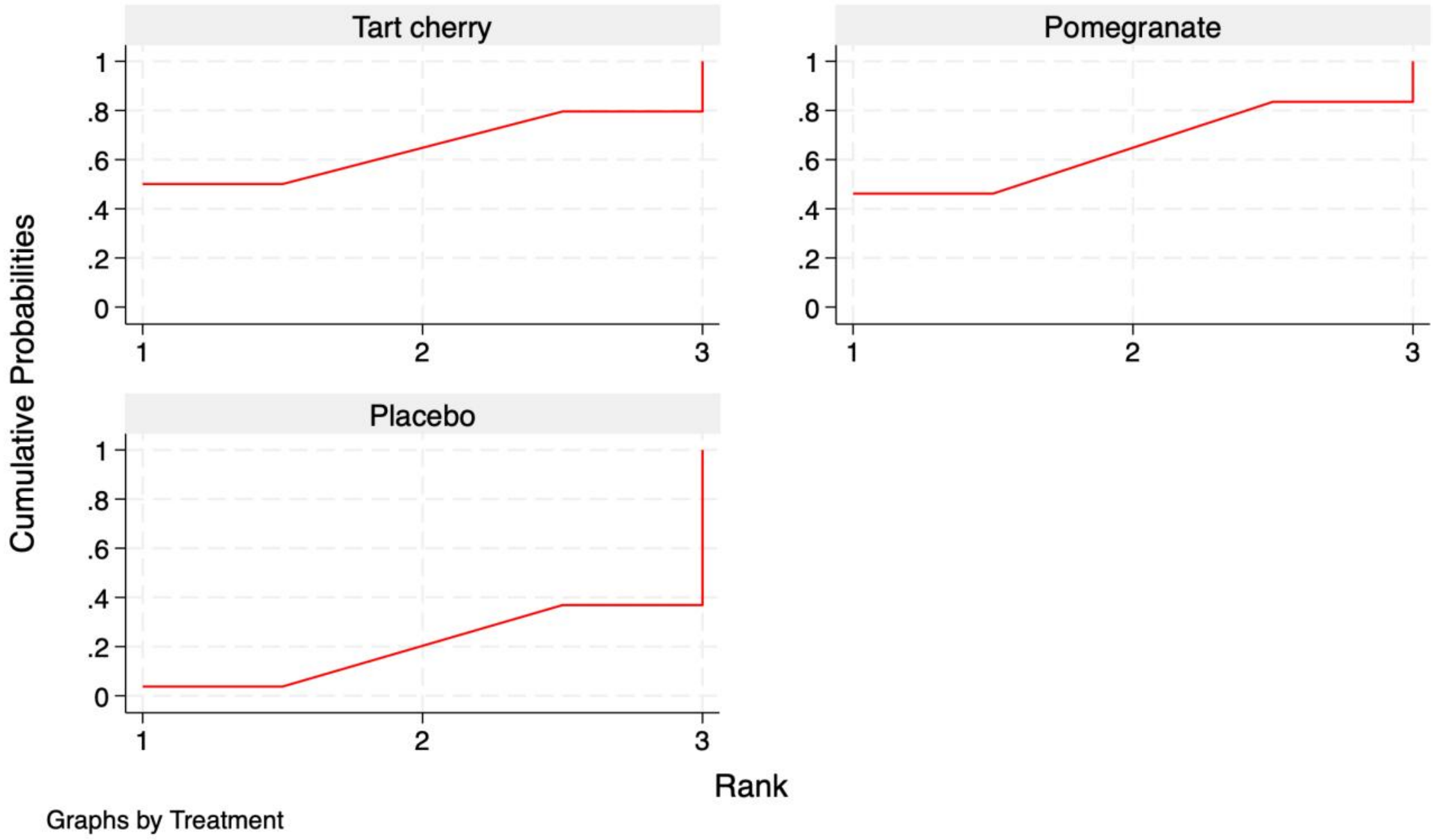

Table S9.12: SUCRA values for the effects of the included interventions on the CRP outcome.

Treatment	SUCRA	PrBest	MeanRank
Tart cherry	64.5	49.9	1.7
Pomegranate	64.8	45.9	1.7
Placebo	20.7	4.1	2.6

Abbreviations: SUCRA, surface under the cumulative ranking curve

Appendix 10: League tables of summary estimates from the network meta-analysis

Table S10.1: DOMS at 48 h Post-Exercise

Each cell presents the mean difference(SMD) and 95% confidence interval for the column intervention versus the row intervention. For DOMS, lower values indicate less pain; therefore, SMD < 0 favors the column intervention, whereas SMD > 0 favors the row intervention. Cells with statistically significant results are colour-coded: pink shading indicates that the column intervention is favored (SMD < 0), green shading indicates that the row intervention is favored (SMD > 0), and white cells indicate no statistically significant difference. The placebo node pools all eligible control conditions, including matched placebo and vehicle-matched controls.

Tart Cherry								
+0.30 (-0.42,1.02)	Pomegranate							
-0.25 (-1.08,0.57)	-0.55 (-1.57,0.47)	Tea Extracts						
-0.72 (-1.49,0.05)	-1.02 (-1.98,-0.06)	-0.47 (-1.51,0.57)	Blueberry					
-0.52 (-1.40,0.37)	-0.82 (-1.87,0.24)	-0.26 (-1.39,0.86)	+0.20 (-0.88,1.29)	Blackcurrant				
-0.44 (-1.35,0.46)	-0.74 (-1.82,0.34)	-0.19 (-1.32,0.95)	+0.28 (-0.82,1.38)	+0.08 (-1.11,1.26)	Cocoa Flavanols			
-0.44 (-1.42,0.55)	-0.74 (-1.88,0.41)	-0.19 (-1.39,1.02)	+0.28 (-0.89,1.45)	+0.08 (-1.17,1.33)	-0.00 (-1.26,1.26)	Grape		
-0.29 (-1.42,0.85)	-0.59 (-1.86,0.69)	-0.04 (-1.36,1.29)	+0.43 (-0.87,1.73)	+0.23 (-1.14,1.60)	+0.15 (-1.23,1.54)	+0.15 (-1.29,1.59)	Mixed Polyphenols	
-0.39 (-0.70,-0.07)	-0.68 (-1.34,-0.02)	-0.13 (-0.89,0.62)	0.34 (-0.37,1.04)	+0.13 (-0.69,0.96)	+0.06 (-0.79,0.90)	+0.05 (-0.88,0.99)	-0.10 (-1.19,1.00)	Placebo

Note: Only pink-shaded off-diagonal cells appear because all statistically significant comparisons favored the column intervention.

Table S10.2: Muscle Strength/Torque at 48 h Post-Exercise

Each cell presents the mean difference (SMD) and 95% confidence interval for the column intervention versus the row intervention. For muscle strength/torque, higher values indicate a more favorable outcome; therefore, SMD > 0 favors the column intervention, whereas SMD < 0 favors the row intervention. White cells indicate no statistically significant difference. The placebo node pools all eligible control conditions, including matched placebo and vehicle-matched controls.

Tart cherry					
+0.23 (-0.59,1.05)	Pomegranate				
+0.29 (-0.93,1.51)	+0.06 (-1.26,1.38)	Blueberry			
+0.25 (-0.73,1.23)	+0.02 (-1.08,1.12)	-0.04 (-1.46,1.38)	Cocoa flavanols		
+0.21 (-0.84,1.27)	-0.02 (-1.18,1.15)	-0.08 (-1.55,1.40)	-0.04 (-1.32,1.24)	Grape	
+0.39 (-0.07,0.85)	+0.16 (-0.52,0.84)	+0.10 (-1.03,1.23)	+0.14 (-0.72,1.00)	+0.18 (-0.77,1.13)	Placebo

Note: No off-diagonal cells were shaded green or pink because none of the pairwise comparisons reached statistical significance.

Table S10.3: CK at 48 h Post-Exercise

Each cell presents the mean difference (SMD) and 95% confidence interval for the column intervention versus the row intervention. For 48-h CK, lower values indicate a more favorable outcome; therefore, SMD < 0 favors the column intervention, whereas SMD > 0 favors the row intervention. Cells with statistically significant results are colour-coded: pink shading indicates that the column intervention is favored (SMD < 0), green shading indicates that the row intervention is favored (SMD > 0), and white cells indicate no statistically significant difference. The placebo node pools all eligible control conditions, including matched placebo and vehicle-matched controls.

Tart cherry							
+0.04<br>(-0.64, 0.71)	Pomegranate						
**-0.78<br>(-1.50, -0.06)**	-0.82<br>(-1.68, 0.05)	Tea extracts					
-0.59<br>(-1.41, 0.23)	-0.62<br>(-1.57, 0.33)	+0.19<br>(-0.79, 1.18)	Blueberry				
+0.70<br>(-0.50, 1.90)	+0.66<br>(-0.64, 1.96)	**+1.48<br>(0.16, 2.80)**	+1.29<br>(-0.09, 2.67)	Blackcurrant			
-0.14<br>(-0.99, 0.71)	-0.18<br>(-1.14 ,0.78)	+0.64<br>(-0.37, 1.65)	+0.45<br>(-0.63, 1.53)	-0.84<br>(-2.24, 0.56)	Cocoa flavanols		
-0.21<br>(-1.27,0.84)	-0.25<br>(-1.41,0.91)	0.57<br>(-0.62,1.76)	0.37<br>(-0.88,1.63)	-0.91<br>(-2.45,0.62)	-0.07<br>(-1.34,1.20)	Mixed Polyphenols	
-0.21<br>(-0.54, 0.12)	-0.25<br>(-0.84, 0.34)	+0.57<br>(-0.07, 1.21)	+0.37<br>(-0.37, 1.12)	-0.91<br>(-2.07, 0.25)	-0.07<br>(-0.85, 0.71)	0.00<br>(-1.00,1.00)	Placebo

Table S10.4: CRP at 48 h Post-Exercise

Each cell presents the mean difference (SMD) and 95% confidence interval for the column intervention versus the row intervention. For 48-h CRP, lower values indicate a more favorable outcome; therefore, SMD < 0 favors the column intervention, whereas SMD > 0 favors the row intervention. Cells with statistically significant results are colour-coded: pink shading indicates that the column intervention is favored (SMD < 0), green shading indicates that the row intervention is favored (SMD > 0), and white cells indicate no statistically significant difference. The placebo node pools all eligible control conditions, including matched placebo and vehicle-matched controls.

**Tart cherry**				
+0.01 (-0.54,0.56)	**Pomegranate**			
-0.58 (-1.54,0.39)	-0.59 (-1.56,0.38)	**Cocoa flavanols**		
+0.25 (-0.72,1.23)	+0.24 (-0.73,1.22)	+0.83 (-0.43,2.09)	Mixed Polyphenols	
-0.24 (-0.63,0.15)	-0.25 (-0.64,0.15)	+0.34 (-0.55,1.22)	-0.49 (-1.39,0.40)	**Placebo**

Note: No off-diagonal cells were shaded green or pink because none of the pairwise comparisons reached statistical significance.

Table S10.5: DOMS at 24 h Post-Exercise

Each cell presents the mean difference (SMD) and 95% confidence interval for the column intervention versus the row intervention. For DOMS, lower values indicate less pain; therefore, SMD < 0 favors the column intervention, whereas SMD > 0 favors the row intervention. Cells with statistically significant results are colour-coded: pink shading indicates that the column intervention is favored (SMD < 0), green shading indicates that the row intervention is favored (SMD > 0), and white cells indicate no statistically significant difference. The placebo node pools all eligible control conditions, including matched placebo and vehicle-matched controls.

**Tart cherry**								
-0.10 (-0.64,0.45)	**Pomegranate**							
0.05 (-0.54,0.64)	+0.15 (-0.58,0.88)	**Tea extracts**						
-0.56 (-1.09,-0.03)	-0.46 (-1.13,0.22)	-0.61 (-1.33,0.11)	**Blueberry**					
-0.47 (-1.17,0.23)	-0.37 (-1.19,0.45)	-0.52 (-1.38,0.34)	+0.09 (-0.73,0.90)	**Blackcurrant**				
-0.35 (-1.07,0.38)	-0.24 (-1.08,0.60)	-0.40 (-1.27,0.48)	+0.21 (-0.62,1.04)	+0.12 (-0.83,1.08)	**Cocoa flavanols**			
-0.41 (-1.08,0.25)	-0.31 (-1.10,0.48)	-0.46 (-1.29,0.36)	+0.14 (-0.64,0.93)	+0.06 (-0.85,0.96)	-0.07 (-0.99,0.86)	**Grape**		
-0.50 (-1.37,0.37)	-0.40 (-1.37,0.57)	-0.55 (-1.55,0.45)	+0.06 (-0.91,1.02)	-0.03 (-1.10,1.04)	-0.15 (-1.24,0.93)	-0.09 (-1.13,0.96)	**Mixed Polyphenols**	
-0.34 (-0.58,-0.10)	-0.23 (-0.72,0.25)	-0.39 (-0.93,0.16)	+0.22 (-0.25,0.69)	+0.13 (-0.53,0.79)	+0.01 (-0.68,0.69)	+0.08 (-0.54,0.70)	+0.16 (-0.68,1.00)	**Placebo**

Note: Only pink-shaded off-diagonal cells appear because all statistically significant comparisons favored the column intervention.

Table S10.6: Muscle Strength/Torque at 24 h Post-Exercise

Each cell presents the mean difference (SMD) and 95% confidence interval for the column intervention versus the row intervention. For muscle strength/torque, higher values indicate a more favorable outcome; therefore, SMD > 0 favors the column intervention, whereas SMD < 0 favors the row intervention. White cells indicate no statistically significant difference. The placebo node pools all eligible control conditions, including matched placebo and vehicle-matched controls.

**Tart cherry**					
+0.36 (-0.19,0.92)	**Pomegranate**				
-0.24 (-1.21,0.72)	-0.61 (-1.60,0.39)	**Blueberry**			
+0.26 (-0.68,1.20)	-0.11 (-1.08,0.87)	+0.50 (-0.75,1.76)	**Cocoa flavanols**		
+0.07 (-0.64,0.78)	-0.29 (-1.05,0.46)	+0.32 (-0.78,1.41)	-0.19 (-1.26,0.89)	**Grape**	
+0.31 (-0.04,0.65)	-0.06 (-0.49,0.37)	+0.55 (-0.35,1.45)	+0.05 (-0.83,0.93)	+0.23 (-0.39,0.86)	**Placebo**

Note: No off-diagonal cells were shaded green or pink because none of the pairwise comparisons reached statistical significance.

Table S10.7: CK at 24 h Post-Exercise

Each cell presents the mean difference (SMD) and 95% confidence interval for the column intervention versus the row intervention. For 24-h CK, lower values indicate a more favorable outcome; therefore, SMD < 0 favors the column intervention, whereas SMD > 0 favors the row intervention. Cells with statistically significant results are colour-coded: pink shading indicates that the column intervention is favored (SMD < 0), green shading indicates that the row intervention is favored (SMD > 0), and white cells indicate no statistically significant difference. The placebo node pools all eligible control conditions, including matched placebo and vehicle-matched controls.

**Tart cherry**							
-0.30 (-0.82,0.22)	**Pomegranate**						
-0.46 (-0.94,0.03)	-0.16 (-0.75,0.42)	**Tea extracts**					
-0.63 (-1.26,0.01)	-0.33 (-1.05,0.38)	-0.17 (-0.86,0.52)	**Blueberry**				
+0.10 (-0.93,1.13)	+0.40 (-0.68,1.48)	+0.56 (-0.50,1.63)	+0.73 (-0.41,1.87)	**Blackcurrant**			
-0.22 (-0.96,0.52)	+0.08 (-0.73,0.89)	+0.24 (-0.55,1.02)	+0.41 (-0.48,1.30)	-0.32 (-1.53,0.88)	**Cocoa flavanols**		
-0.07 (-0.99,0.86)	+0.23 (-0.75,1.21)	+0.39 (-0.57,1.35)	+0.56 (-0.48,1.61)	-0.17 (-1.49,1.16)	+0.15 (-0.96,1.27)	**Mixed Polyphenols**	
-0.25 (-0.54,0.03)	+0.05 (-0.39,0.48)	+0.21 (-0.18,0.60)	+0.38 (-0.19,0.94)	-0.36 (-1.35,0.64)	-0.03 (-0.71,0.65)	-0.19 (-1.06,0.69)	**Placebo**

Note: No green-shaded and pink shading cells appear in this table as no row intervention significantly outperformed its comparator.

Table S10.8: CRP at 24 h Post-Exercise

Each cell presents the mean difference (SMD) and 95% confidence interval for the column intervention versus the row intervention. For 24-h CRP, lower values indicate a more favorable outcome; therefore,SMD < 0 favors the column intervention, whereas SMD > 0 favors the row intervention. Cells with statistically significant results are colour-coded: pink shading indicates that the column intervention is favored (SMD < 0), green shading indicates that the row intervention is favored (SMD > 0), and white cells indicate no statistically significant difference. The placebo node pools all eligible control conditions, including matched placebo and vehicle-matched controls.

**Tart cherry**				
+0.07 (-0.65,0.78)	**Pomegranate**			
-0.35 (-1.45,0.75)	-0.41 (-1.58,0.75)	**Cocoa flavanols**		
-0.02 (-1.12,1.08)	-0.09 (-1.25,1.08)	+0.33 (-1.11,1.77)	Mixed Polyphenols	
-0.25 (-0.67,0.18)	-0.31 (-0.88,0.26)	+0.10 (-0.91,1.12)	-0.22 (-1.24,0.79)	**Placebo**

Note: No off-diagonal cells were shaded green or pink because none of the pairwise comparisons reached statistical significance.

Table S10.9: DOMS at 72 h Post-Exercise

Each cell presents the mean difference (SMD) and 95% confidence interval for the column intervention versus the row intervention. For DOMS, lower values indicate less pain; therefore, SMD < 0 favors the column intervention, whereas SMD > 0 favors the row intervention. Cells with statistically significant results are colour-coded: pink shading indicates that the column intervention is favored (SMD < 0), green shading indicates that the row intervention is favored (SMD > 0), and white cells indicate no statistically significant difference. The placebo node pools all eligible control conditions, including matched placebo and vehicle-matched controls.

**Tart cherry**				
-0.24 (-1.08,0.60)	**Pomegranate**			
-0.92 (-1.93,0.10)	-0.68 (-1.80,0.45)	**Blueberry**		
-0.42 (-1.71,0.88)	-0.18 (-1.56,1.21)	+0.50 (-0.99,1.99)	**Blackcurrant**	
-0.56 (-1.04,-0.07)	-0.31 (-1.00,0.37)	+0.36 (-0.53,1.25)	-0.14 (-1.34,1.06)	**Placebo**

Note: Only pink-shaded off-diagonal cells appear because all statistically significant comparisons favored the column intervention.

Table S10.10: Muscle Strength/Torque at 72 h Post-Exercise

Each cell presents the mean difference (SMD) and 95% confidence interval for the column intervention versus the row intervention. For muscle strength/torque, higher values indicate a more favorable outcome; therefore, SMD > 0 favors the column intervention, whereas SMD < 0 favors the row intervention. White cells indicate no statistically significant difference. The placebo node pools all eligible control conditions, including matched placebo and vehicle-matched controls.

**Tart cherry**		
-0.01 (-0.65,0.63)	**Pomegranate**	
+0.29 (-0.18,0.77)	+0.30 (-0.13,0.73)	**Placebo**

Note: No off-diagonal cells were shaded green or pink because none of the pairwise comparisons reached statistical significance.

Table S10.11: CK at 72 h Post-Exercise

Each cell presents the mean difference (SMD) and 95% confidence interval for the column intervention versus the row intervention. For 72-h CK, lower values indicate a more favorable outcome; therefore, SMD < 0 favors the column intervention, whereas SMD > 0 favors the row intervention. Cells with statistically significant results are colour-coded: pink shading indicates that the column intervention is favored (SMD < 0), green shading indicates that the row intervention is favored (SMD > 0), and white cells indicate no statistically significant difference. The placebo node pools all eligible control conditions, including matched placebo and vehicle-matched controls.

**Tart cherry**			
-0.17 (-1.03,0.69)	**Pomegranate**		
-0.43 (-1.46,0.60)	-0.25 (-1.32,0.81)	**Blueberry**	
-0.04 (-0.62,0.55)	+0.14 (-0.50,0.78)	+0.39 (-0.46,1.24)	**Placebo**

Note: No off-diagonal cells were shaded green or pink because none of the pairwise comparisons reached statistical significance.

Table S10.12: CRP at 72 h Post-Exercise

Each cell presents the mean difference (SMD) and 95% confidence interval for the column intervention versus the row intervention. For 72-h CRP, lower values indicate a more favorable outcome; therefore, SMD < 0 favors the column intervention, whereas SMD > 0 favors the row intervention. Cells with statistically significant results are colour-coded: pink shading indicates that the column intervention is favored (SMD < 0), green shading indicates that the row intervention is favored (SMD > 0), and white cells indicate no statistically significant difference. The placebo node pools all eligible control conditions, including matched placebo and vehicle-matched controls.

**Tart cherry**		
-0.01 (-0.71,0.69)	**Pomegranate**	
-0.21 (-0.76,0.34)	-0.20 (-0.64,0.23)	**Placebo**

Note: No off-diagonal cells were shaded green or pink because none of the pairwise comparisons reached statistical significance.

Appendix 11: CINeMA Assessment

The certainty of evidence for each network estimate was evaluated using the CINeMA approach. Within-study bias was classified into three levels: low, moderate, and high risk of bias. Studies were considered at moderate risk when none of the five assessed domains showed high risk, whereas studies were judged to be at high risk when one or more domains were rated as high risk. The detailed risk-of-bias assessment is shown in Appendix 5, while the risk-of-bias contribution for each pairwise treatment comparison is displayed in Figures S11.1 – S11.8.

Figure S11.1: Risk of bias contribution by intervention group for 48-h DOMS

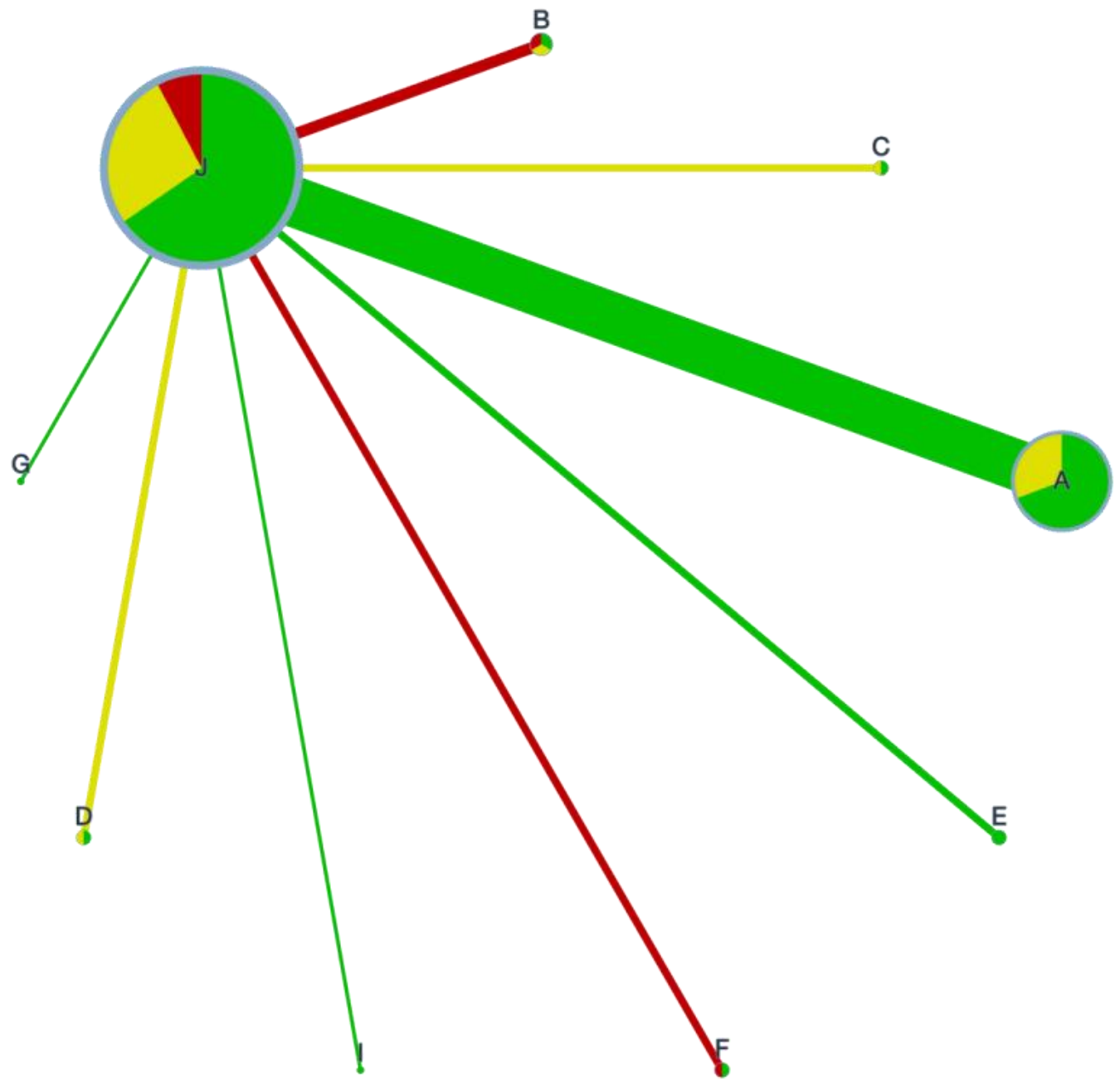

Figure S11.2: Overall risk of bias by treatment comparison in 48-h DOMS

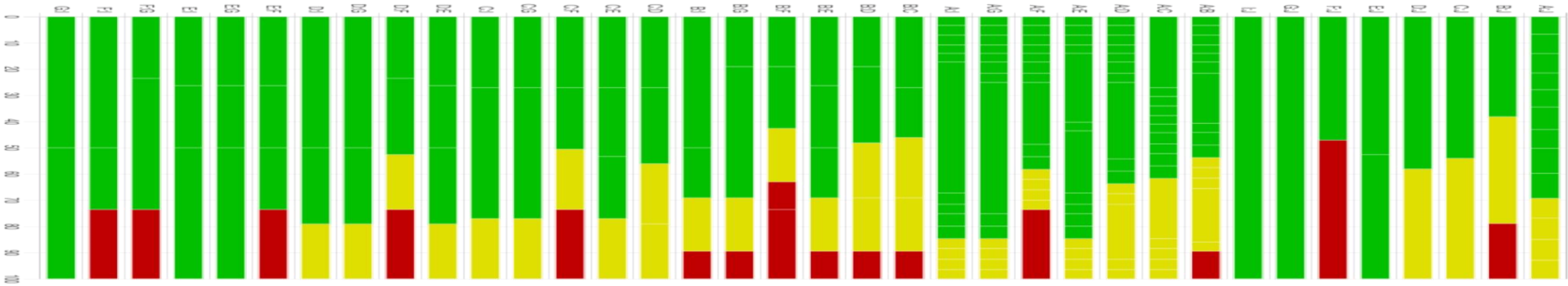

Figure S11.3: Risk of bias contribution by intervention group for 48-h Muscle Strength

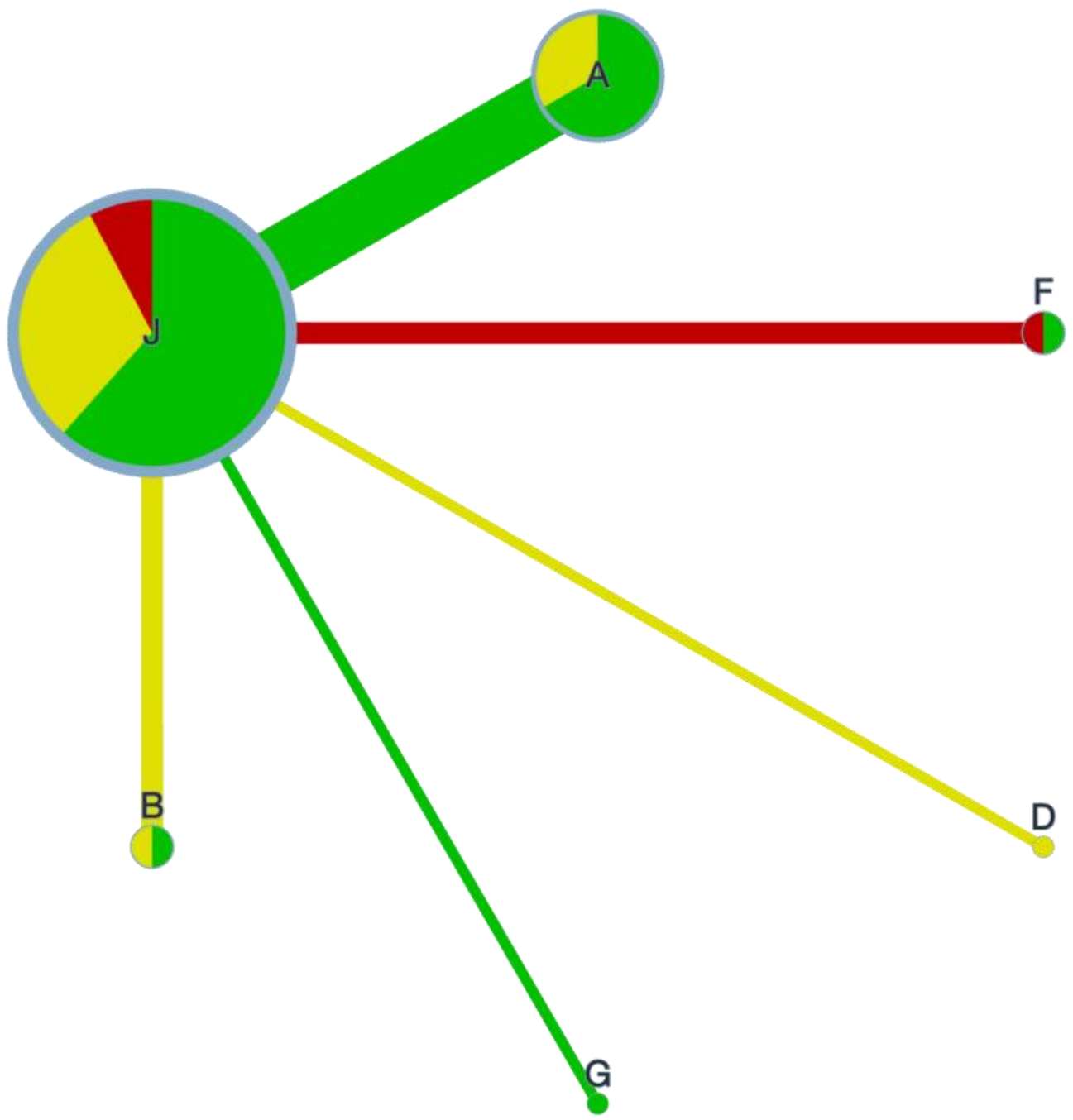

Figure S11.4: Overall risk of bias by treatment comparison in Muscle Strength

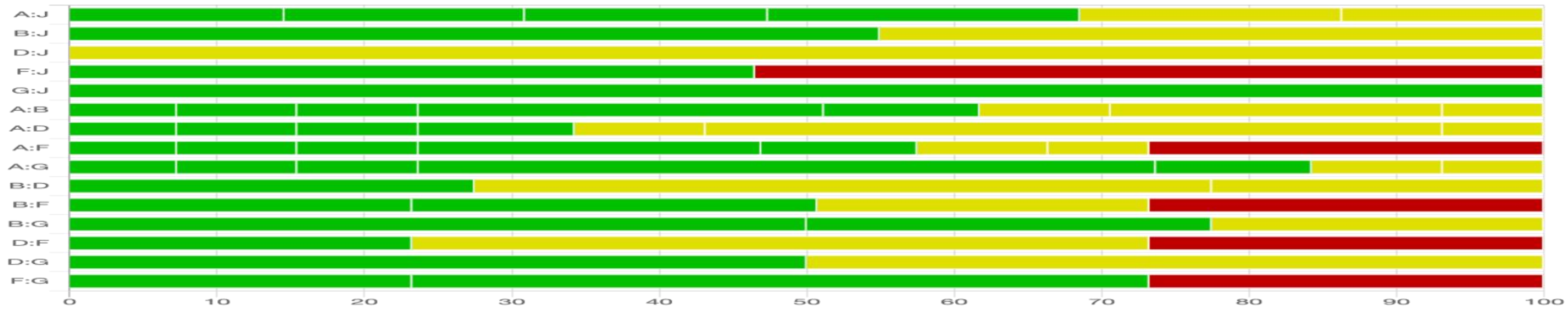

Figure S11.5: Risk of bias contribution by intervention group for 48-h CK

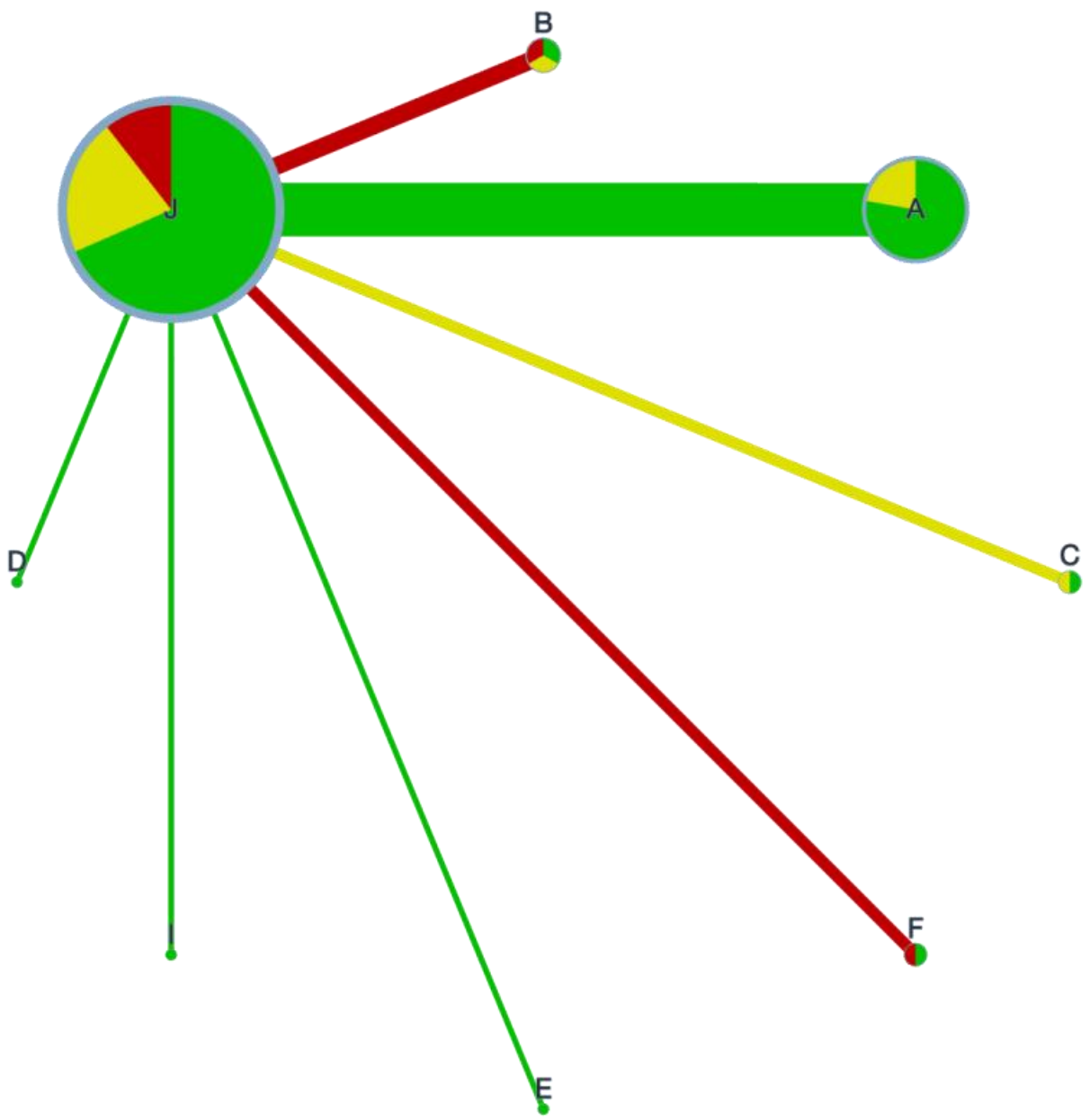

Figure S11.6: Overall risk of bias by treatment comparison in 48-h CK

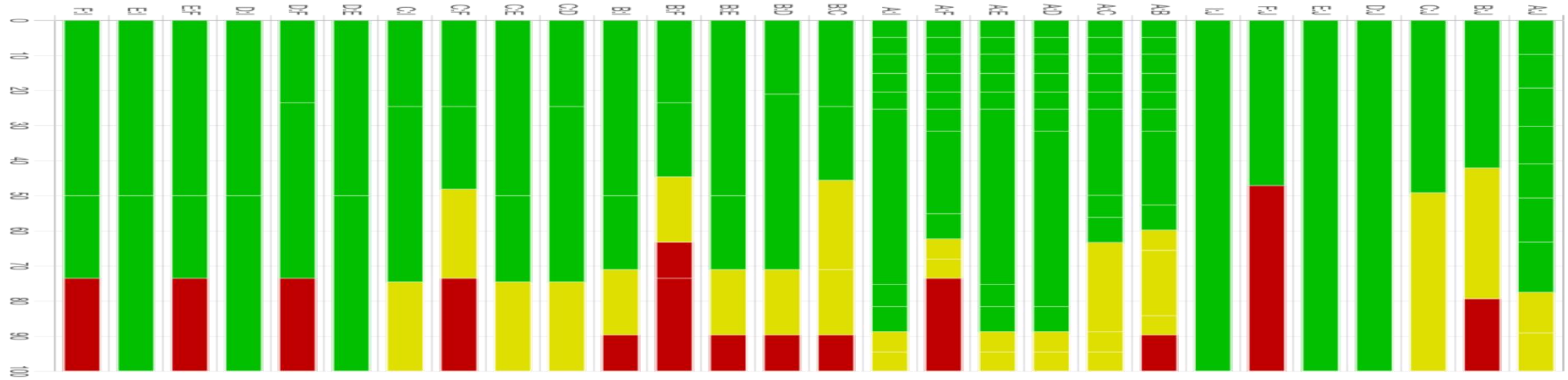

Figure S11.7: Risk of bias contribution by intervention group for 48-h CRP

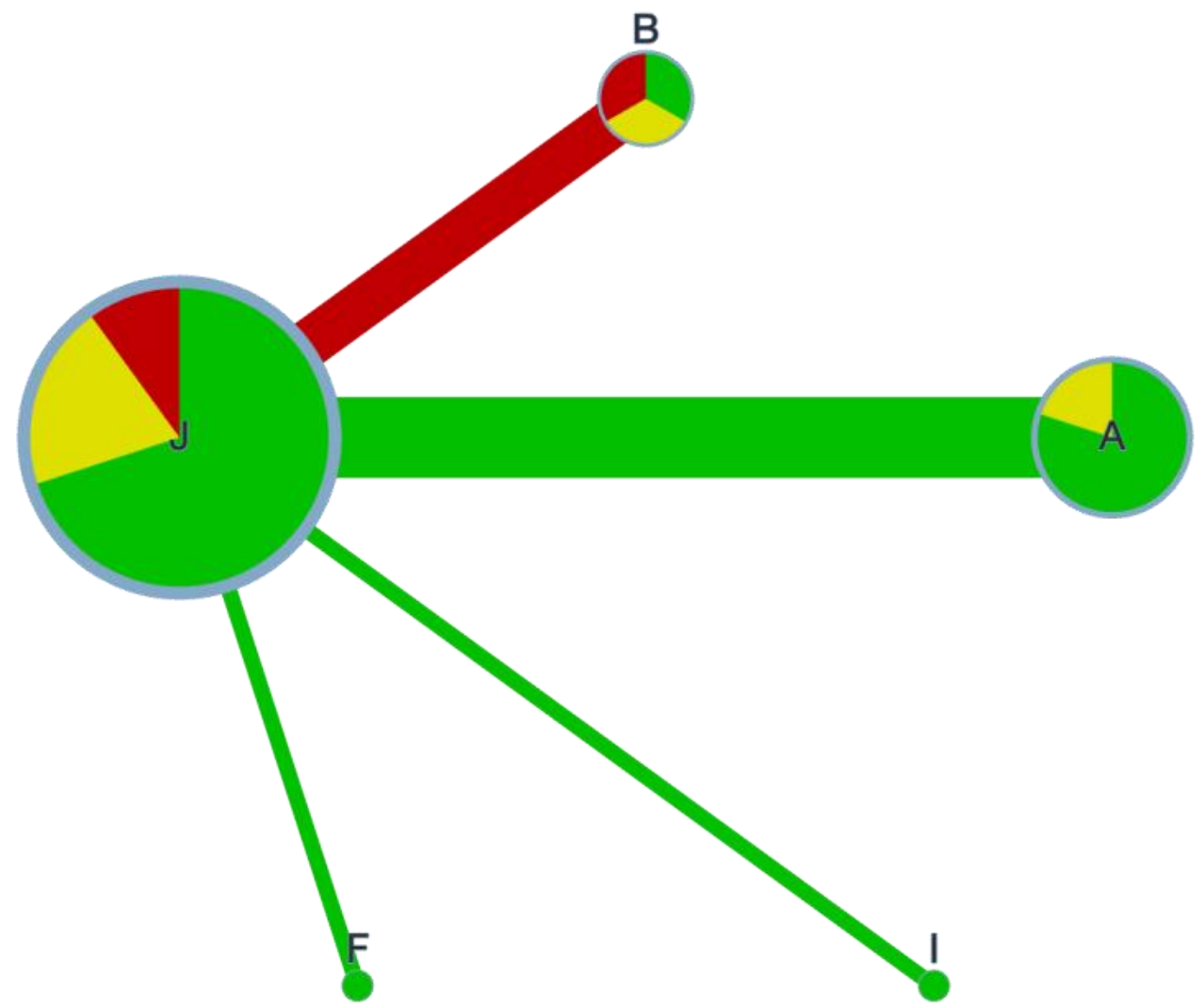

Figure S11.8: Overall risk of bias by treatment comparison in 48-h CRP

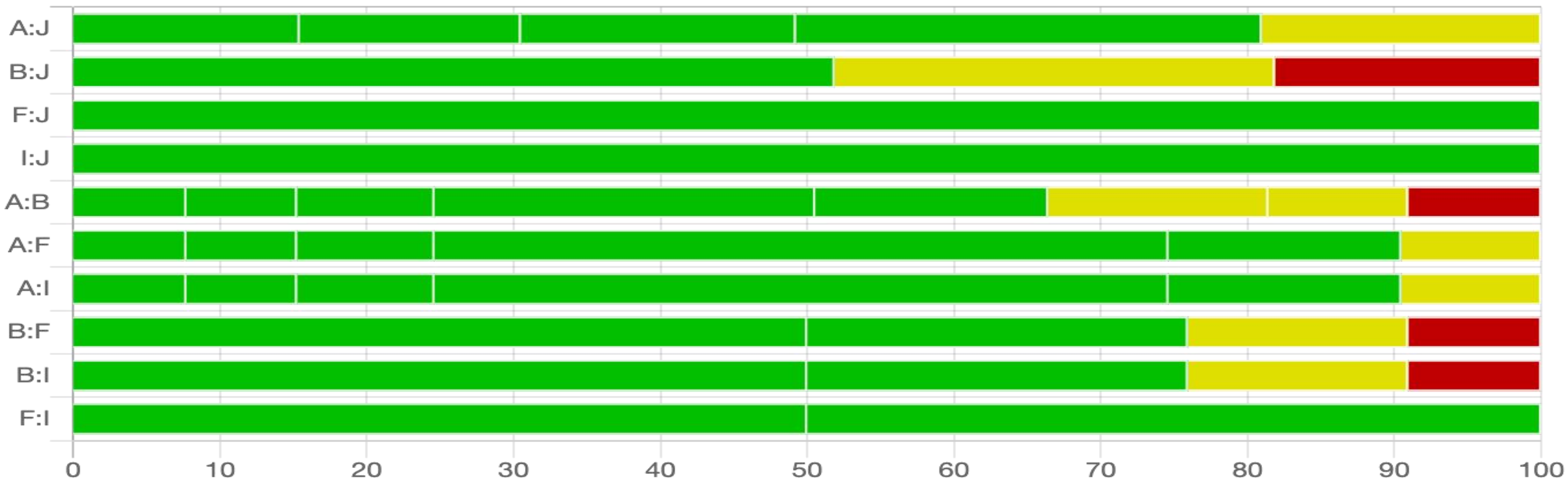

Table S11.1: Transitivity (Indirectness) Assessment

	Baseline variable (Mean ± SD)		
Intervention	Age (years)	Training status	Supplement timing
Tart cherry	24.78 ± 7.09	Mostly trained	Mostly chronic peri-exercise
Pomegranate	27.29 ± 8.75	Mostly trained	Mostly chronic peri-exercise
Tea extracts	21.89 ± 2.71	Mixed	Mixed
Blueberry	33.71 ± 7.93	Mixed	Mostly chronic peri-exercise
Blackcurrant	25.33 ± 6.11	Mostly untrained	Mostly chronic peri-exercise
Cocoa flavanols	21.61 ± 2.61	Mixed	Mostly chronic peri-exercise
Grape	19.85 ± 1.96	Mostly untrained	Mostly chronic peri-exercise
Mixed Polyphenols	22.9 ± 2.83	Mostly trained	Mostly chronic peri-exercise

Imprecision: Certainty of evidence for each network estimate was evaluated using the CINeMA approach. Imprecision was assessed by examining whether the 95% confidence intervals crossed the prespecified threshold for a clinically important effect, in accordance with CINeMA recommendations.

Heterogeneity: The level of concern regarding heterogeneity was determined by comparing both confidence intervals and prediction intervals with the same threshold for clinical importance used in the imprecision assessment. Judgments were made following the CINeMA framework.

Inconsistency: Inconsistency was not formally assessed using node-splitting because the present network did not include closed loops. As a result, local inconsistency testing was not applicable. The plausibility of indirect comparisons was therefore considered mainly through the assessment of transitivity and network geometry.

Table S11.2: CINeMA Results of 48-h DOMS

Comparison	Within-study bias	Reporting bias	Indirectness	Imprecision	Heterogeneity	Incoherence	Confidence rating
Tart cherry vs Placebo	No concerns	Low risk	No concerns	No concerns	Major concerns	Major concerns	Low
Pomegranate vs Placebo	Some concerns	Low risk	No concerns	No concerns	Major concerns	Major concerns	Low
Tea extracts vs Placebo	No concerns	Low risk	No concerns	Major concerns	No concerns	Major concerns	Low
Blueberry vs Placebo	No concerns	Low risk	No concerns	Major concerns	No concerns	Major concerns	Low
Blackcurrant vs Placebo	No concerns	Low risk	No concerns	Major concerns	No concerns	Major concerns	Low
Cocoa flavanols vs Placebo	Major concerns	Low risk	No concerns	Major concerns	No concerns	Major concerns	Low
Grape vs Placebo	No concerns	Low risk	No concerns	Major concerns	No concerns	Major concerns	Low
Mixed Polyphenols vs Placebo	No concerns	Low risk	No concerns	Major concerns	No concerns	Major concerns	Low
Tart cherry vs Pomegranate	No concerns	Low risk	No concerns	Major concerns	No concerns	Major concerns	Low
Tart cherry vs Tea extracts	No concerns	Low risk	No concerns	Major concerns	No concerns	Major concerns	Low
Tart cherry vs Blueberry	No concerns	Low risk	No concerns	Some concerns	Some concerns	Major concerns	Low
Tart cherry vs Blackcurrant	No concerns	Low risk	No concerns	Major concerns	No concerns	Major concerns	Low
Tart cherry vs Cocoa flavanols	No concerns	Low risk	No concerns	Major concerns	No concerns	Major concerns	Low

Tart cherry vs Grape	No concerns	Low risk	No concerns	Major concerns	No concerns	Major concerns	Low
Tart cherry vs Mixed Polyphenols	No concerns	Low risk	No concerns	Major concerns	No concerns	Major concerns	Low
Pomegranate vs Tea extracts	No concerns	Low risk	No concerns	Major concerns	No concerns	Major concerns	Low
Pomegranate vs Blueberry	No concerns	Low risk	No concerns	No concerns	Major concerns	Major concerns	Low
Pomegranate vs Blackcurrant	No concerns	Low risk	No concerns	Major concerns	No concerns	Major concerns	Low
Pomegranate vs Cocoa flavanols	No concerns	Low risk	No concerns	Major concerns	No concerns	Major concerns	Low
Pomegranate vs Grape	No concerns	Low risk	No concerns	Major concerns	No concerns	Major concerns	Low
Pomegranate vs Mixed Polyphenols	No concerns	Low risk	No concerns	Major concerns	No concerns	Major concerns	Low
Tea extracts vs Blueberry	No concerns	Low risk	No concerns	Major concerns	No concerns	Major concerns	Low
Tea extracts vs Blackcurrant	No concerns	Low risk	No concerns	Major concerns	No concerns	Major concerns	Low
Tea extracts vs Cocoa flavanols	No concerns	Low risk	No concerns	Major concerns	No concerns	Major concerns	Low
Tea extracts vs Grape	No concerns	Low risk	No concerns	Major concerns	No concerns	Major concerns	Low
Tea extracts vs Mixed Polyphenols	No concerns	Low risk	No concerns	Major concerns	No concerns	Major concerns	Low
Blueberry vs Blackcurrant	No concerns	Low risk	No concerns	Major concerns	No concerns	Major concerns	Low
Blueberry vs Cocoa flavanols	No concerns	Low risk	No concerns	Major concerns	No concerns	Major concerns	Low
Blueberry vs Grape	No concerns	Low risk	No concerns	Major concerns	No concerns	Major concerns	Low
Blueberry vs Mixed Polyphenols	No concerns	Low risk	No concerns	Major concerns	No concerns	Major concerns	Low
Blackcurrant vs Cocoa flavanols	No concerns	Low risk	No concerns	Major concerns	No concerns	Major concerns	Low
Blackcurrant vs Grape	No concerns	Low risk	No concerns	Major concerns	No concerns	Major concerns	Low
Blackcurrant vs Mixed Polyphenols	No concerns	Low risk	No concerns	Major concerns	No concerns	Major concerns	Low
Cocoa flavanols vs Grape	No concerns	Low risk	No concerns	Major concerns	No concerns	Major concerns	Low
Cocoa flavanols vs Mixed Polyphenols	No concerns	Low risk	No concerns	Major concerns	No concerns	Major concerns	Low
Grape vs Mixed Polyphenols	No concerns	Low risk	No concerns	Major concerns	No concerns	Major concerns	Low

Note: The placebo node pools all eligible control conditions, including matched placebo and vehicle-matched controls.

Table S11.3: CINeMA Results of 48-h Muscle Strength

Comparison	Within-study bias	Reporting bias	Indirectness	Imprecision	Heterogeneity	Incoherence	Confidence rating
Tart cherry vs Placebo	No concerns	Low risk	No concerns	Major concerns	No concerns	Major concerns	Low
Pomegranate vs Placebo	No concerns	Low risk	No concerns	Major concerns	No concerns	Major concerns	Low
Blueberry vs Placebo	Some concerns	Low risk	No concerns	Major concerns	No concerns	Major concerns	Low
Cocoa flavanols vs Placebo	Major concerns	Low risk	No concerns	Major concerns	No concerns	Major concerns	Low
Grape vs Placebo	No concerns	Low risk	No concerns	Major concerns	No concerns	Major concerns	Low
Tart cherry vs Pomegranate	No concerns	Low risk	No concerns	Major concerns	No concerns	Major concerns	Low
Tart cherry vs Blueberry	Some concerns	Low risk	No concerns	Major concerns	No concerns	Major concerns	Low
Tart cherry vs Cocoa flavanols	No concerns	Low risk	No concerns	Major concerns	No concerns	Major concerns	Low
Tart cherry vs Grape	No concerns	Low risk	No concerns	Major concerns	No concerns	Major concerns	Low
Pomegranate vs Blueberry	Some concerns	Low risk	No concerns	Major concerns	No concerns	Major concerns	Low
Pomegranate vs Cocoa flavanols	No concerns	Low risk	No concerns	Major concerns	No concerns	Major concerns	Low
Pomegranate vs Grape	No concerns	Low risk	No concerns	Major concerns	No concerns	Major concerns	Low
Blueberry vs Cocoa flavanols	Some concerns	Low risk	No concerns	Major concerns	No concerns	Major concerns	Low
Blueberry vs Grape	No concerns	Low risk	No concerns	Major concerns	No concerns	Major concerns	Low
Cocoa flavanols vs Grape	No concerns	Low risk	No concerns	Major concerns	No concerns	Major concerns	Low

Note: The placebo node pools all eligible control conditions, including matched placebo and vehicle-matched control

Table S11.4: CINeMA Results of 48-h CK

Comparison	Within-study bias	Reporting bias	Indirectness	Imprecision	Heterogeneity	Incoherence	Confidence rating
Tart cherry vs Placebo	No concerns	Low risk	No concerns	Major concerns	No concerns	Major concerns	Low
Pomegranate vs Placebo	No concerns	Low risk	No concerns	Major concerns	No concerns	Major concerns	Low
Tea extracts vs Placebo	Some concerns	Low risk	No concerns	Major concerns	No concerns	Major concerns	Low
Blueberry vs Placebo	No concerns	Low risk	No concerns	Major concerns	No concerns	Major concerns	Low
Blackcurrant vs Placebo	No concerns	Low risk	No concerns	Major concerns	No concerns	Major concerns	Low
Cocoa flavanols vs Placebo	Major concerns	Low risk	No concerns	Major concerns	No concerns	Major concerns	Low
Mixed Polyphenols vs Placebo	No concerns	Low risk	No concerns	Major concerns	No concerns	Major concerns	Low
Tart cherry vs Pomegranate	No concerns	Low risk	No concerns	Major concerns	No concerns	Major concerns	Low
Tart cherry vs Tea extracts	No concerns	Low risk	No concerns	Major concerns	No concerns	Major concerns	Low
Tart cherry vs Blueberry	No concerns	Low risk	No concerns	Major concerns	No concerns	Major concerns	Low
Tart cherry vs Blackcurrant	No concerns	Low risk	No concerns	Major concerns	No concerns	Major concerns	Low
Tart cherry vs Cocoa flavanols	No concerns	Low risk	No concerns	Major concerns	No concerns	Major concerns	Low
Tart cherry vs Mixed Polyphenols	No concerns	Low risk	No concerns	Major concerns	No concerns	Major concerns	Low
Pomegranate vs Tea extracts	No concerns	Low risk	No concerns	Major concerns	No concerns	Major concerns	Low
Pomegranate vs Blueberry	No concerns	Low risk	No concerns	Major concerns	No concerns	Major concerns	Low

Pomegranate vs Blackcurrant	No concerns	Low risk	No concerns	Major concerns	No concerns	Major concerns	Low
Pomegranate vs Cocoa flavanols	No concerns	Low risk	No concerns	Major concerns	No concerns	Major concerns	Low
Pomegranate vs Mixed Polyphenols	No concerns	Low risk	No concerns	Major concerns	No concerns	Major concerns	Low
Tea extracts vs Blueberry	No concerns	Low risk	No concerns	Major concerns	No concerns	Major concerns	Low
Tea extracts vs Blackcurrant	No concerns	Low risk	No concerns	No concerns	Major concerns	Major concerns	Low
Tea extracts vs Cocoa flavanols	No concerns	Low risk	No concerns	Major concerns	No concerns	Major concerns	Low
Tea extracts vs Mixed Polyphenols	No concerns	Low risk	No concerns	Major concerns	No concerns	Major concerns	Low
Blueberry vs Blackcurrant	No concerns	Low risk	No concerns	Major concerns	No concerns	Major concerns	Low
Blueberry vs Cocoa flavanols	No concerns	Low risk	No concerns	Major concerns	No concerns	Major concerns	Low
Blueberry vs Mixed Polyphenols	No concerns	Low risk	No concerns	Major concerns	No concerns	Major concerns	Low
Blackcurrant vs Cocoa flavanols	No concerns	Low risk	No concerns	Major concerns	No concerns	Major concerns	Low
Blackcurrant vs Mixed Polyphenols	No concerns	Low risk	No concerns	Major concerns	No concerns	Major concerns	Low
Cocoa flavanols vs Mixed Polyphenols	No concerns	Low risk	No concerns	Major concerns	No concerns	Major concerns	Low

Note: The placebo node pools all eligible control conditions, including matched placebo and vehicle-matched controls

Table S11.5: CINeMA Results of 48-h CRP

Comparison	Within-study bias	Reporting bias	Indirectness	Imprecision	Heterogeneity	Incoherence	Confidence rating
Tart cherry vs Placebo	No concerns	Low risk	No concerns	Major concerns	No concerns	No concerns	Low
Pomegranate vs Placebo	No concerns	Low risk	No concerns	Major concerns	No concerns	No concerns	Low
Cocoa flavanols vs Placebo	No concerns	Low risk	No concerns	Major concerns	No concerns	No concerns	Low
Mixed Polyphenols vs Placebo	No concerns	Low risk	No concerns	Major concerns	No concerns	No concerns	Low
Tart cherry vs Pomegranate	No concerns	Low risk	No concerns	Major concerns	No concerns	No concerns	Low
Tart cherry vs Cocoa flavanols	No concerns	Low risk	No concerns	Major concerns	No concerns	No concerns	Low
Tart cherry vs Mixed Polyphenols	No concerns	Low risk	No concerns	Major concerns	No concerns	No concerns	Low
Pomegranate vs Cocoa flavanols	No concerns	Low risk	No concerns	Major concerns	No concerns	No concerns	Low
Pomegranate vs Mixed Polyphenols	No concerns	Low risk	No concerns	Major concerns	No concerns	No concerns	Low
Cocoa flavanols vs Mixed Polyphenols	No concerns	Low risk	No concerns	Major concerns	No concerns	No concerns	Low

Note: The placebo node pools all eligible control conditions, including matched placebo and vehicle-matched controls

Appendix 12: Funnel plots

The funnel plots assess small-study effects and potential publication bias for the network meta-analysis comparing plant-derived polyphenol supplements on exercise-induced muscle damage outcomes. Each funnel plot includes all pairwise comparisons between each active intervention and placebo. Egger's test was used to formally assess funnel plot asymmetry; a P-value > 0.05 was considered indicative of no significant asymmetry, suggesting the absence of substantial publication bias, whereas P < 0.05 was interpreted as evidence of potential small-study effects.

Figure S12.1: DOMS at 48 h Post-Exercise

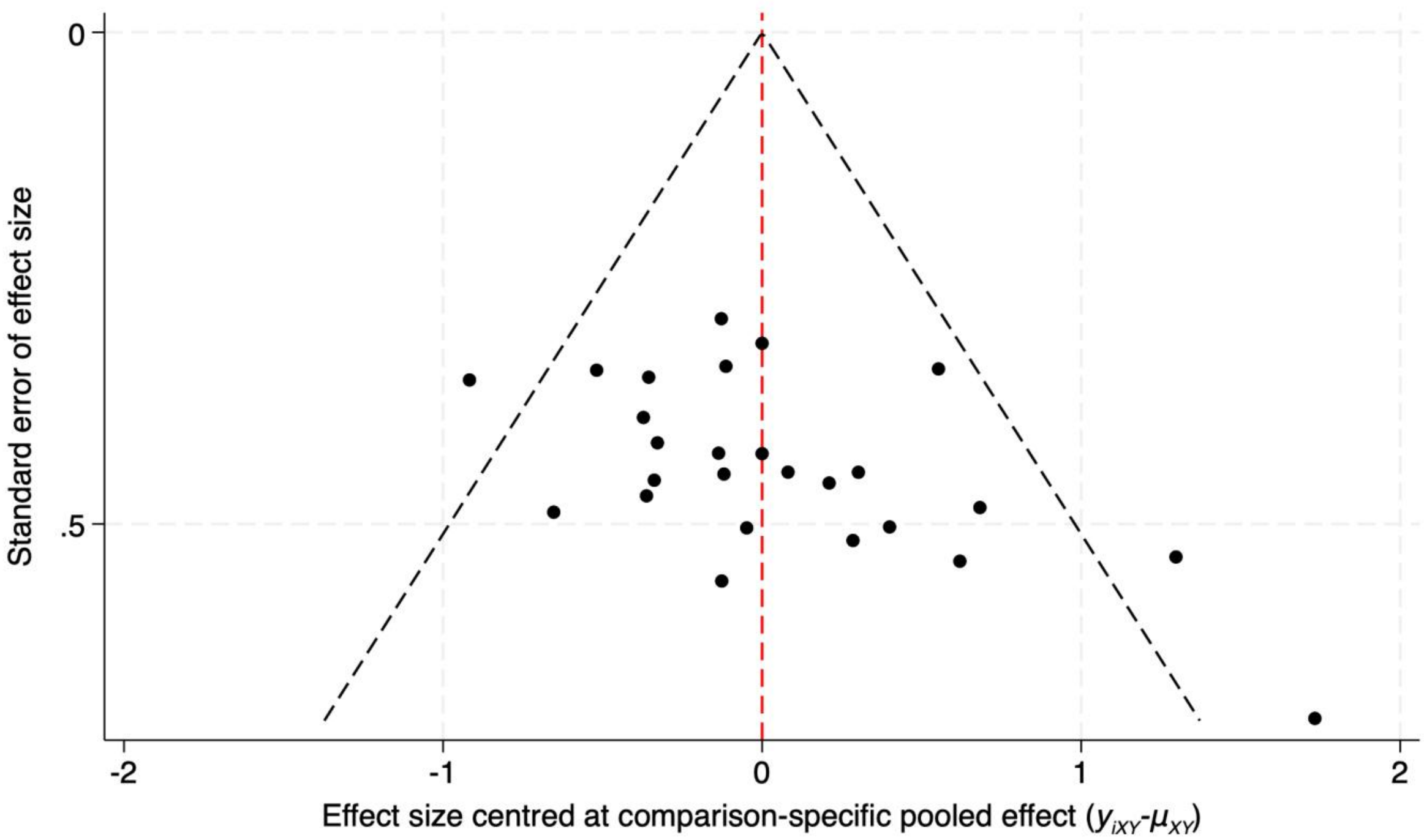

Egger's test t=2.93 P=0.007

Figure S12.2: Muscle Strength/Torque at 48 h Post-Exercise

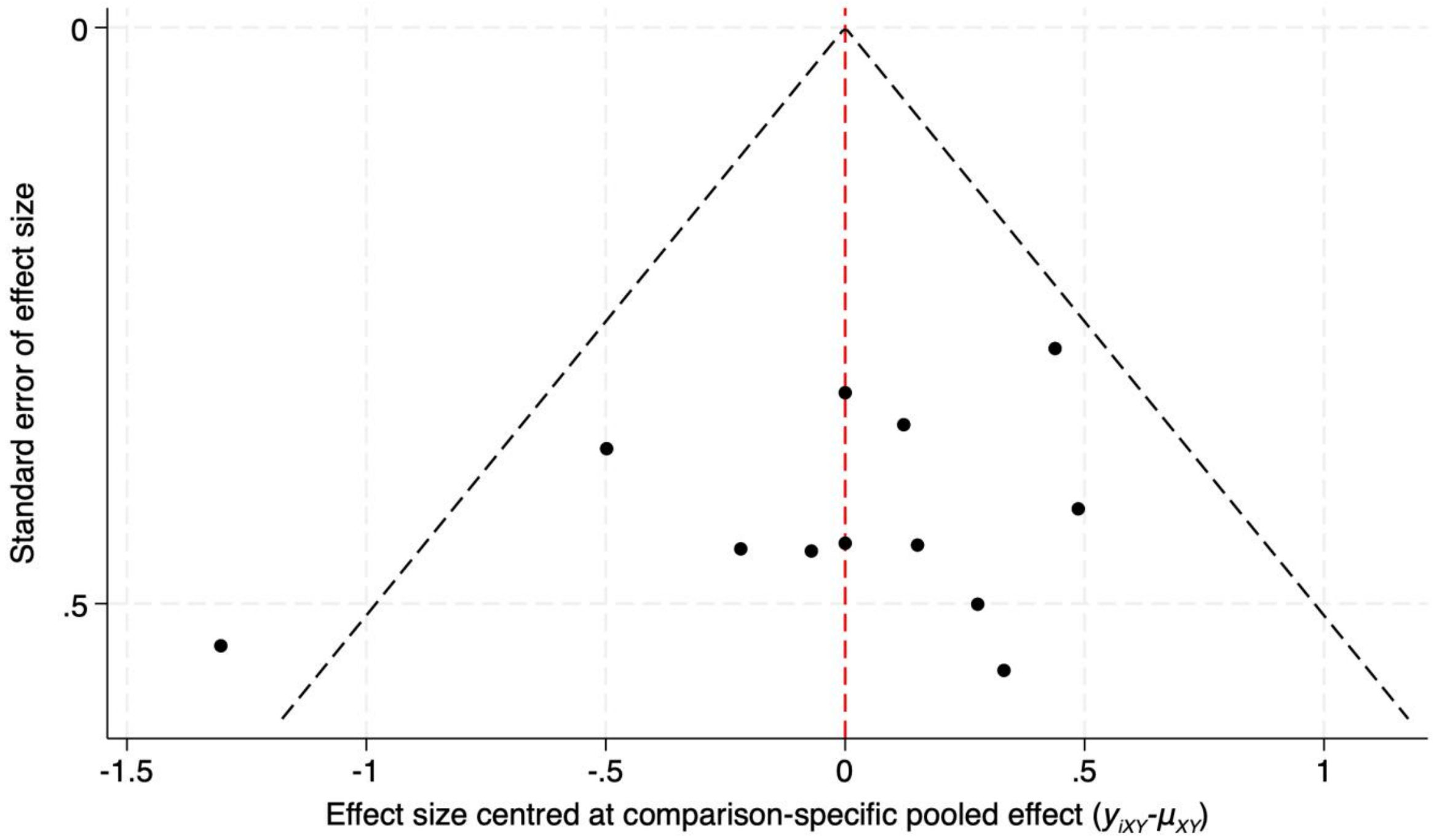

Egger's test t= -1.48 P= 0.169

Figure S12.3: CK at 48 h Post-Exercise

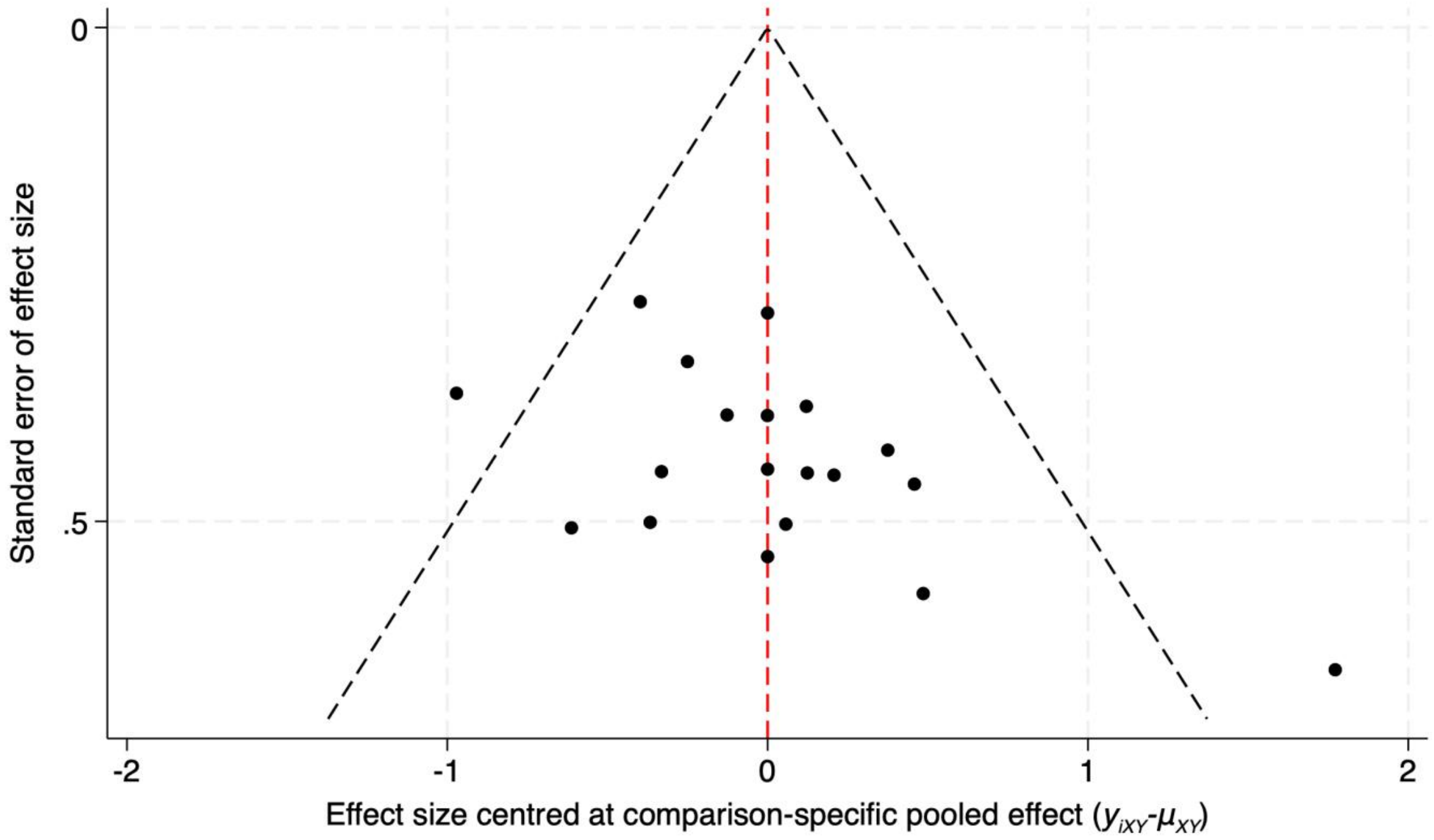

Egger's test t=-2.66 P=0.017

Figure S12.4: CRP at 48 h Post-Exercise

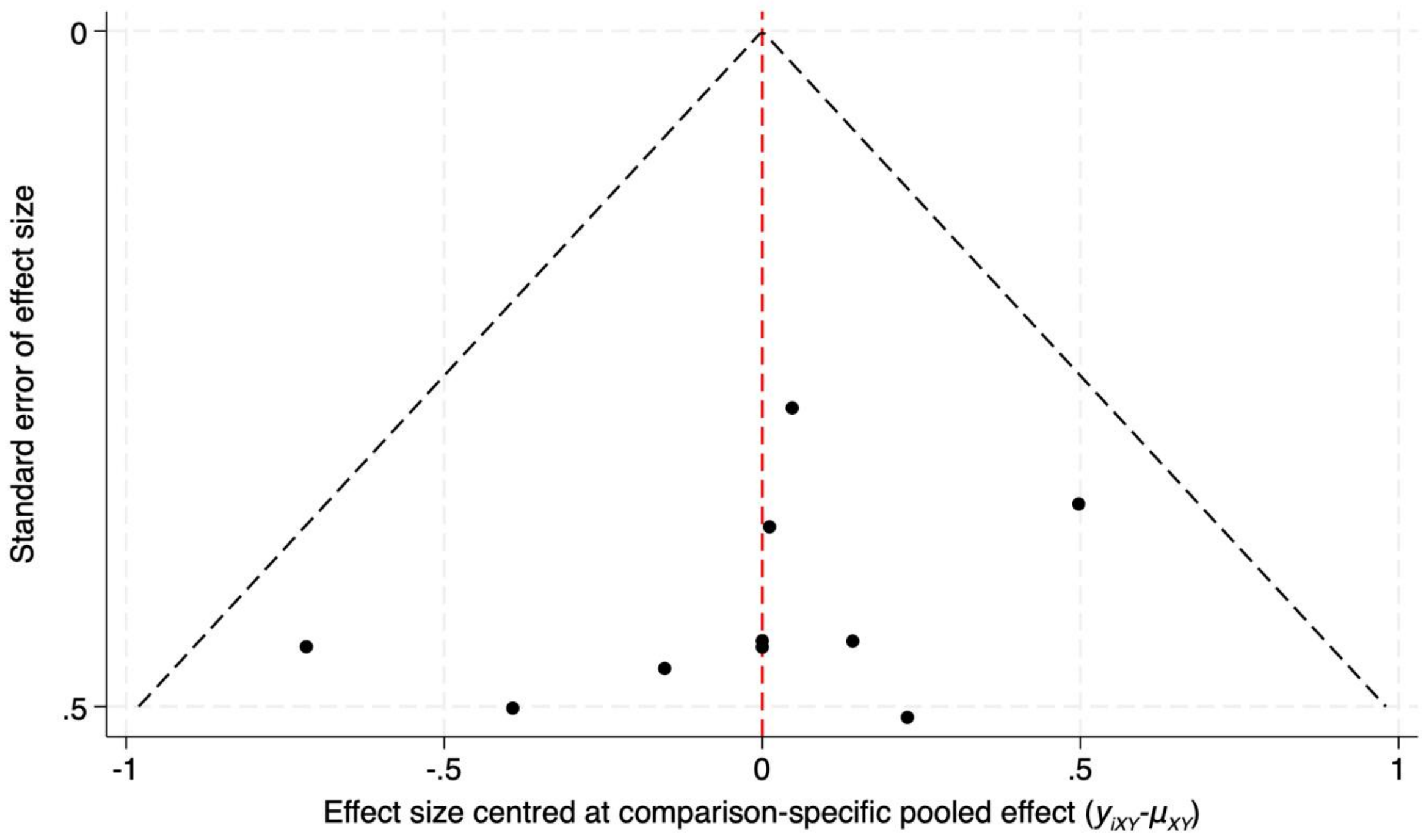

Egger's test t=-1.20 P=0.263

Figure S12.5: DOMS at 24 h Post-Exercise

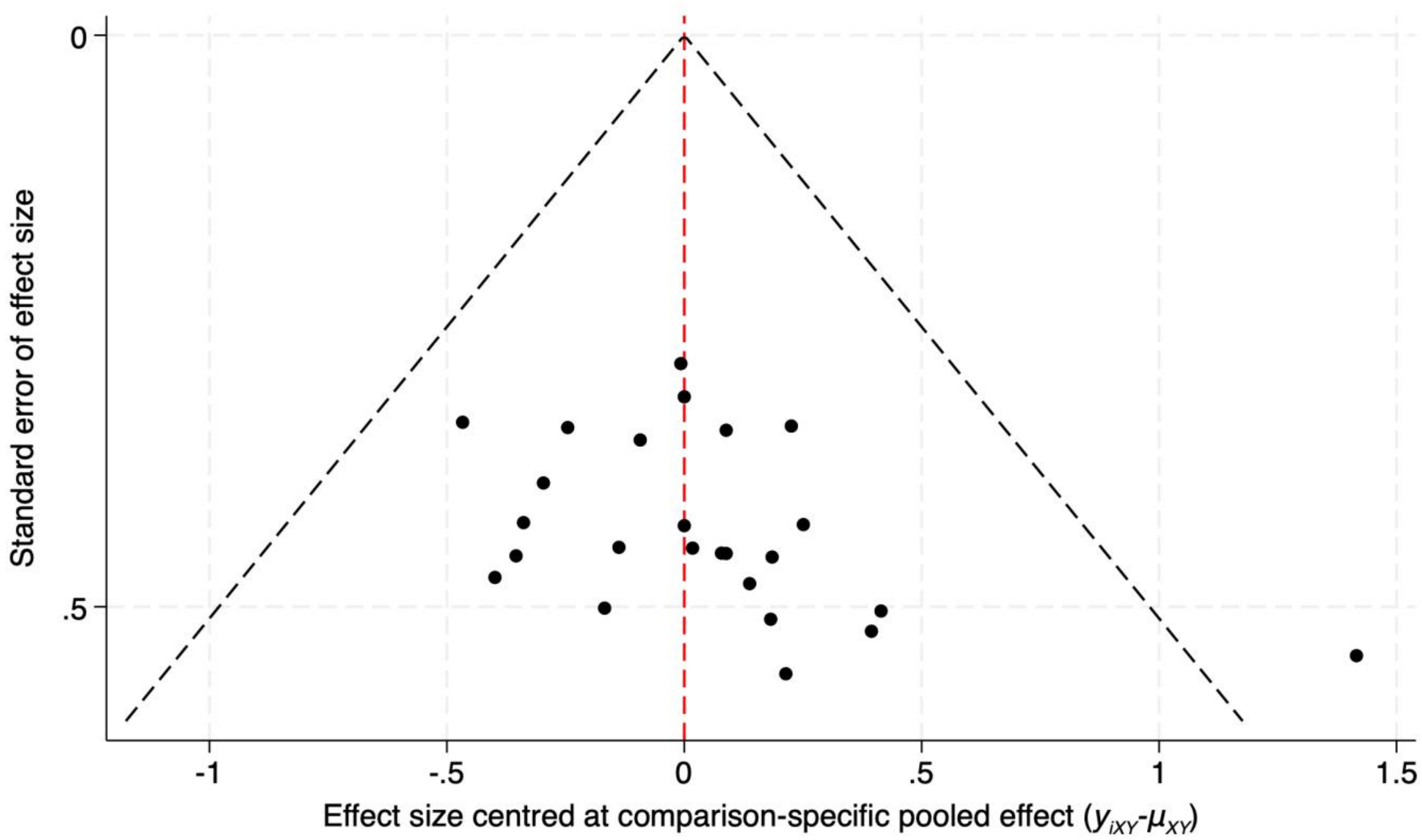

Egger's test t=2.26 P=0.034

Figure S12.6: Muscle Strength/Torque at 24 h Post-Exercise

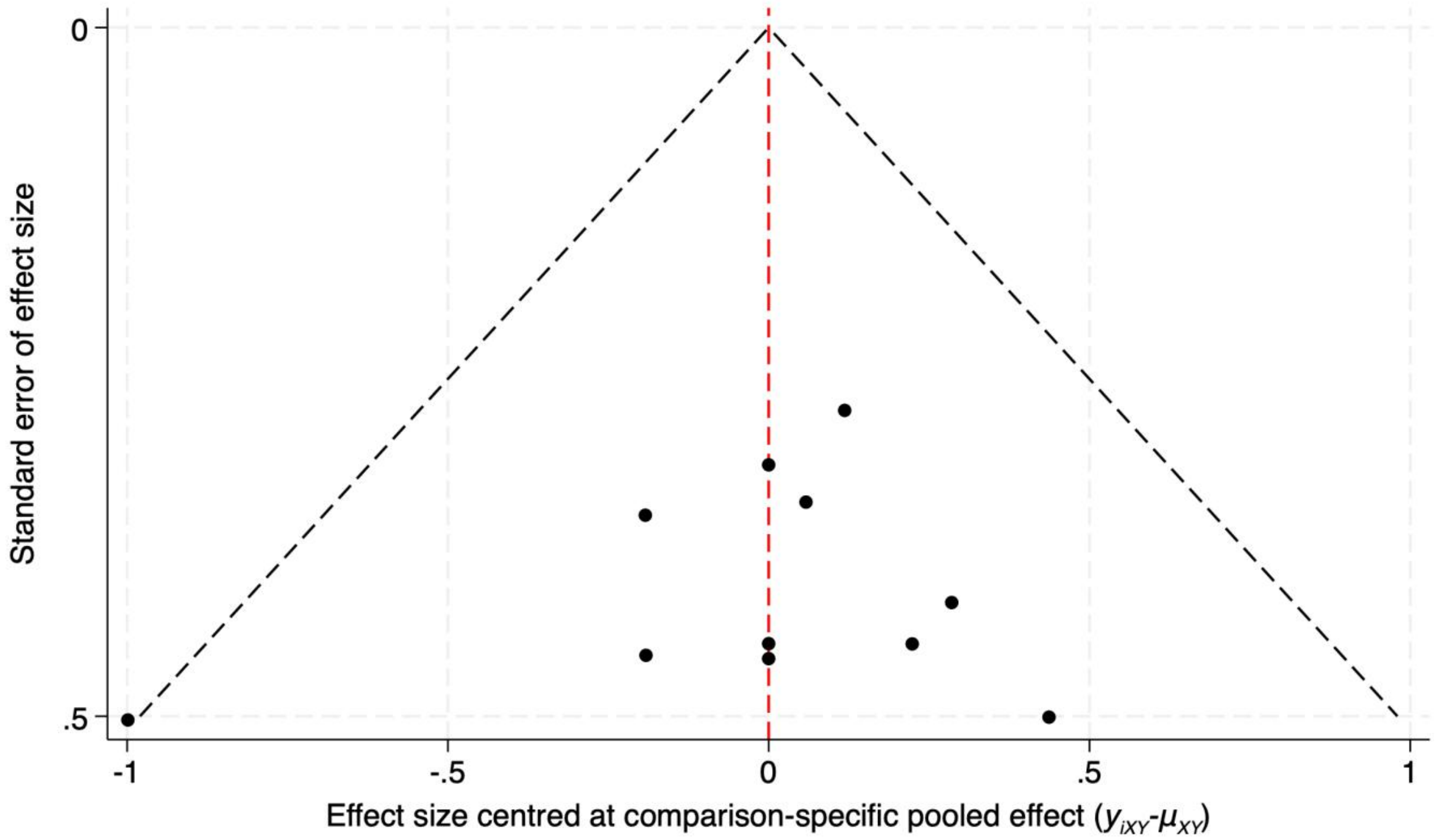

Egger's test t= -1.79 P= 0.107

Figure S12.7: CK at 24 h Post-Exercise

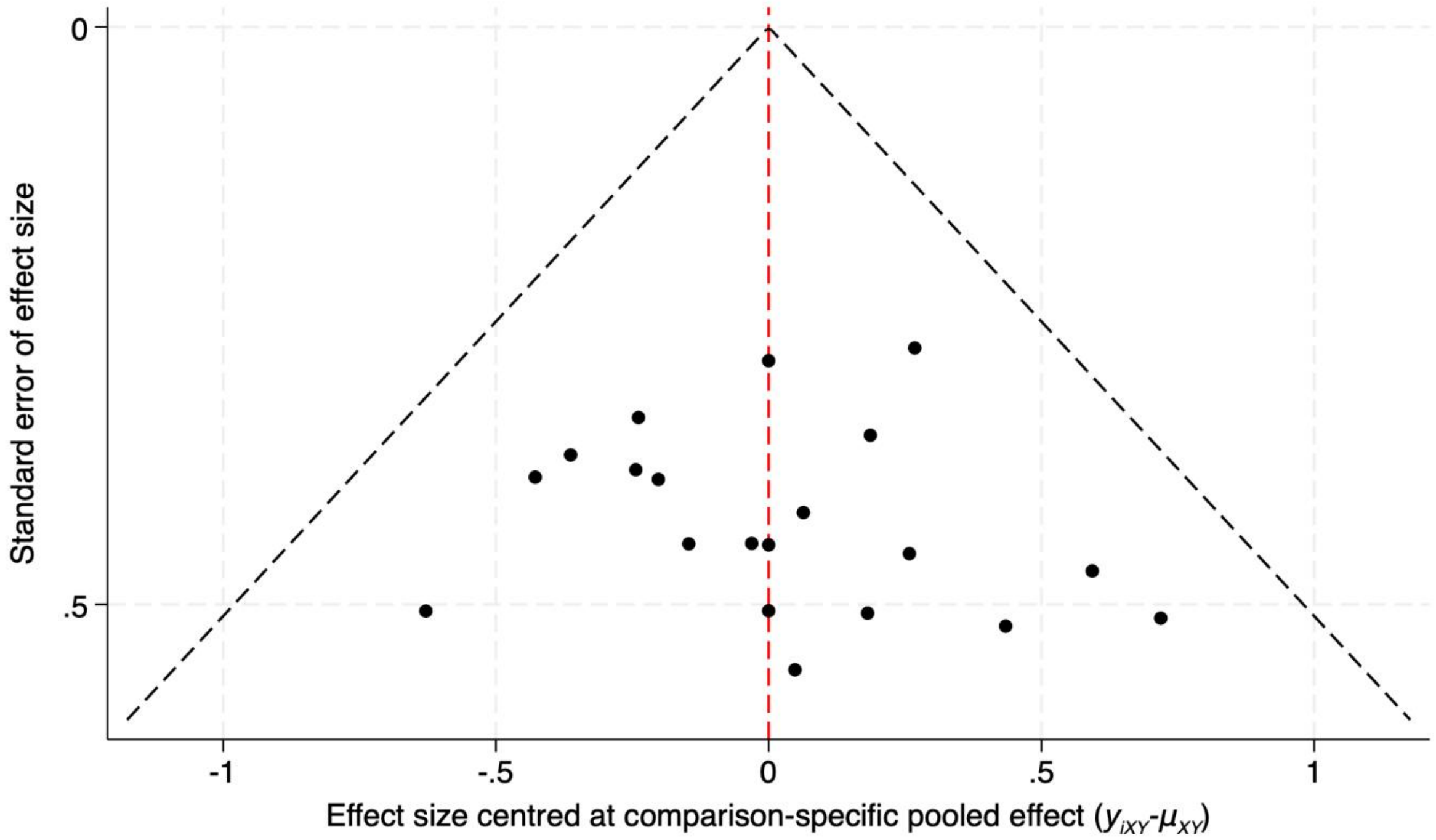

Egger's test t=2.14 P=0.046

Figure S12.8: CRP at 24 h Post-Exercise

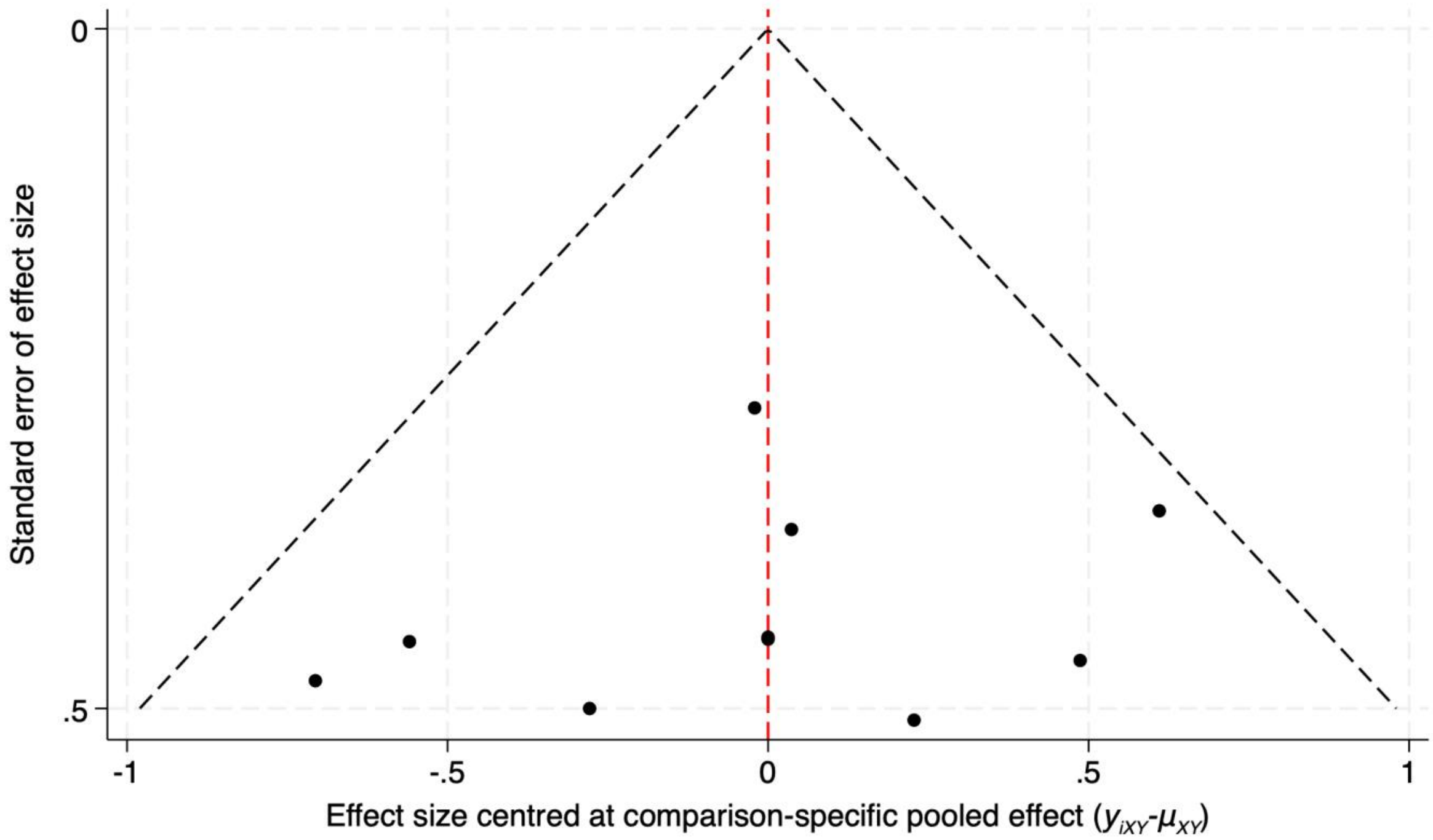

Egger's test t= -1.09 P=0.307

Figure S12.9: DOMS at 72 h Post-Exercise

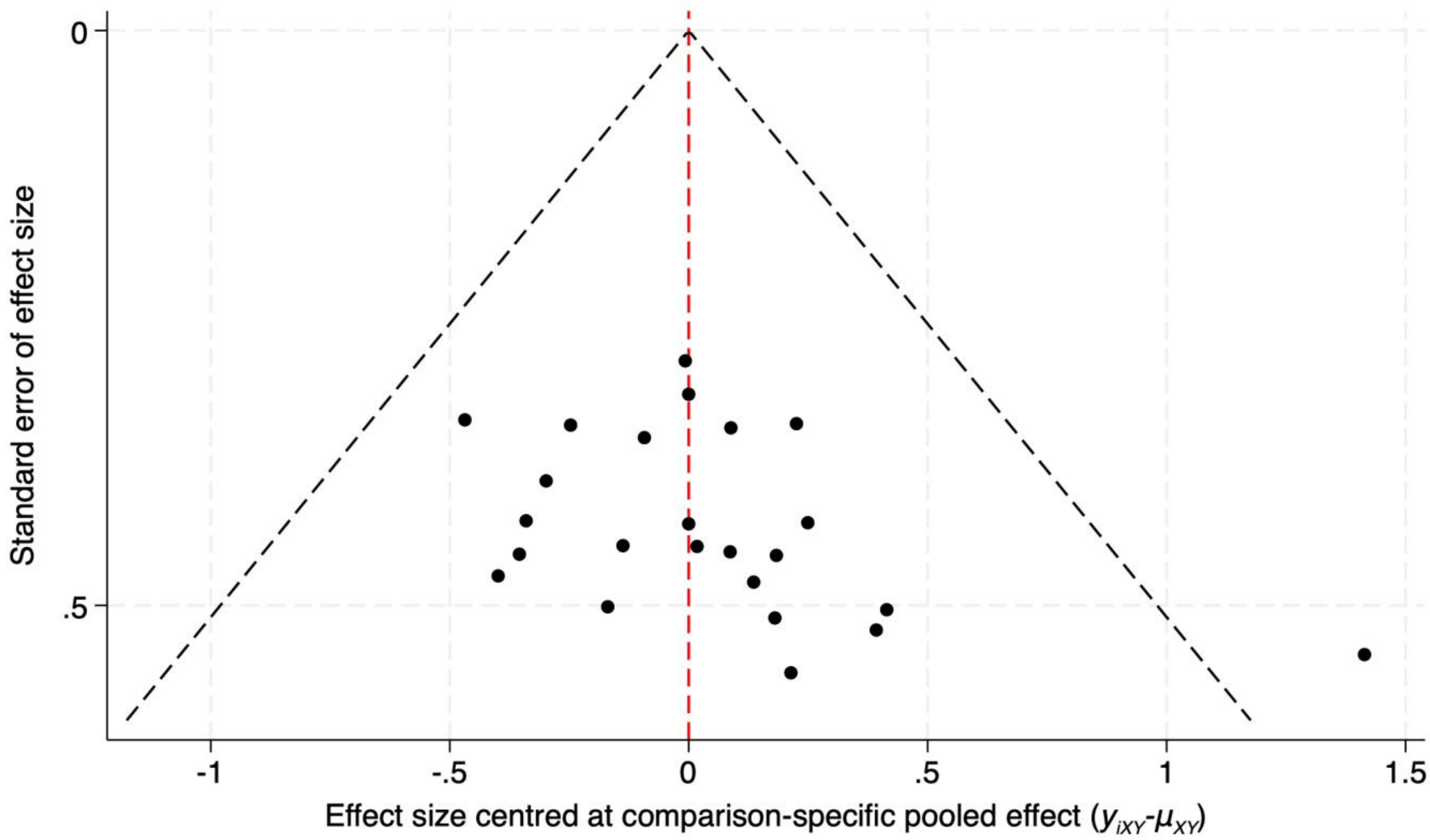

Egger's test t=3.69 P=0.006

Figure S12.10: Muscle Strength/Torque at 72 h Post-Exercise

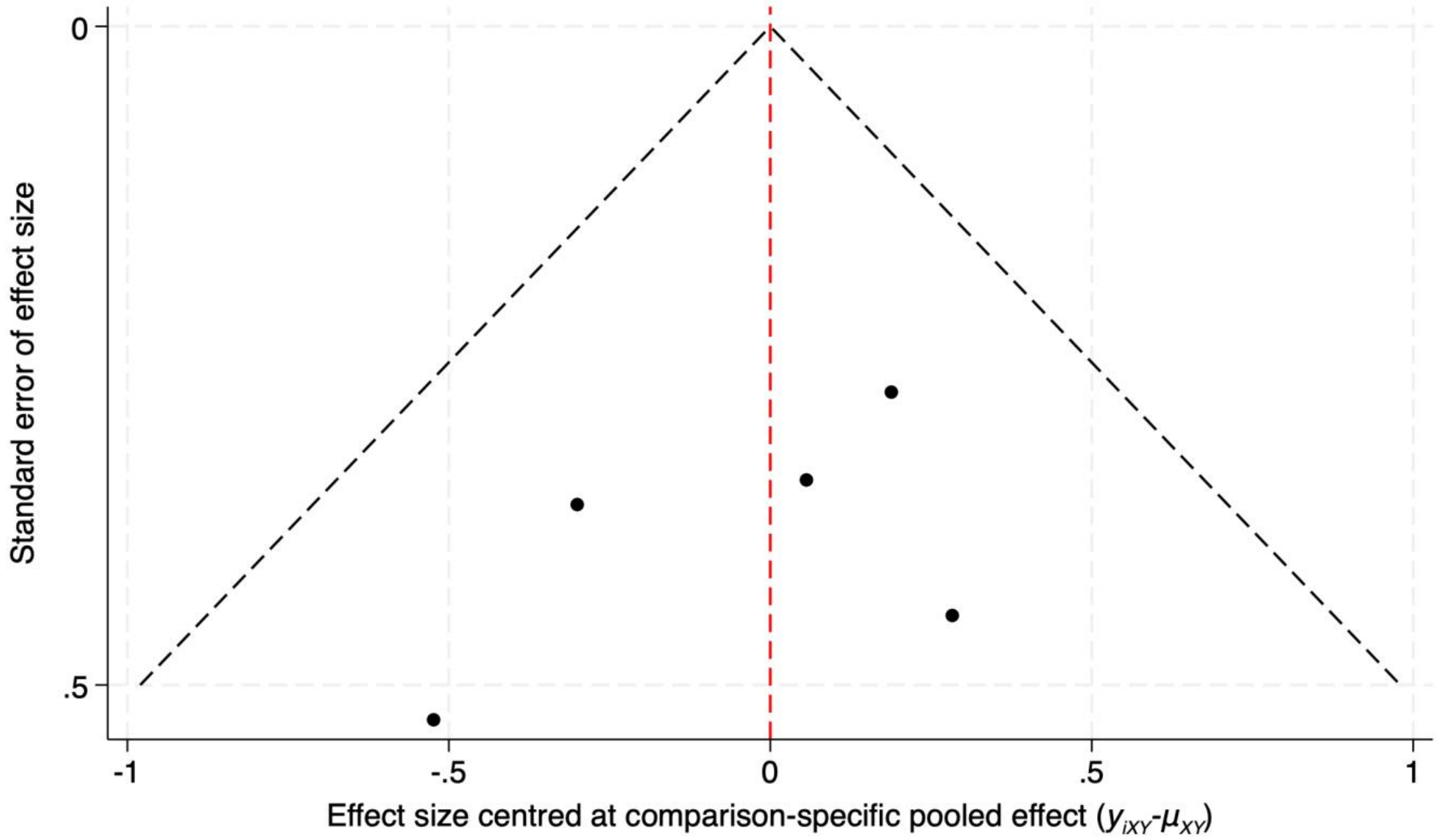

Egger's test t= -1.03 P= 0.379

Figure S12.11: CK at 72 h Post-Exercise

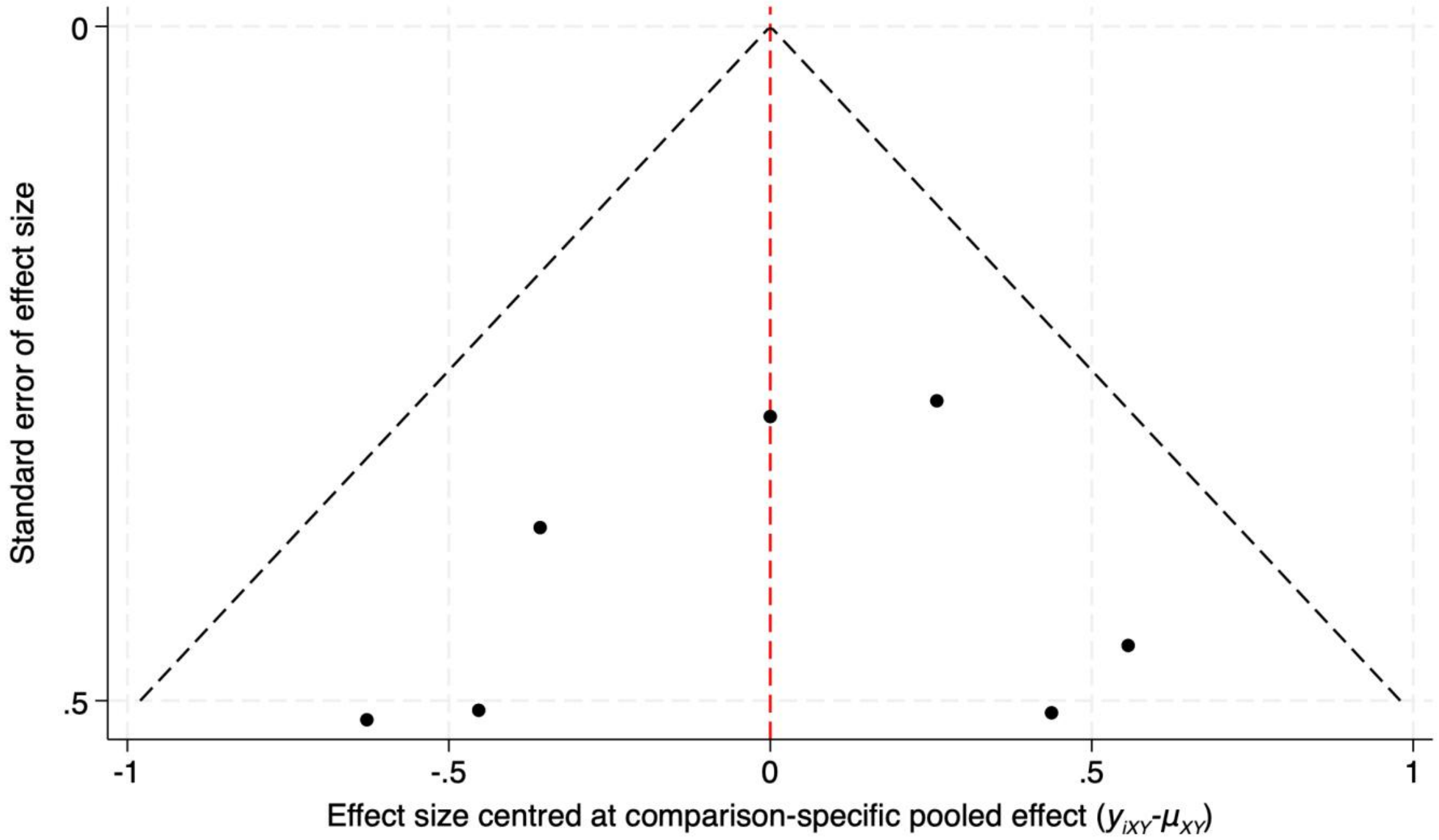

Egger's test t=0.20 P=0.849

Figure S12.12: CRP at 72 h Post-Exercise

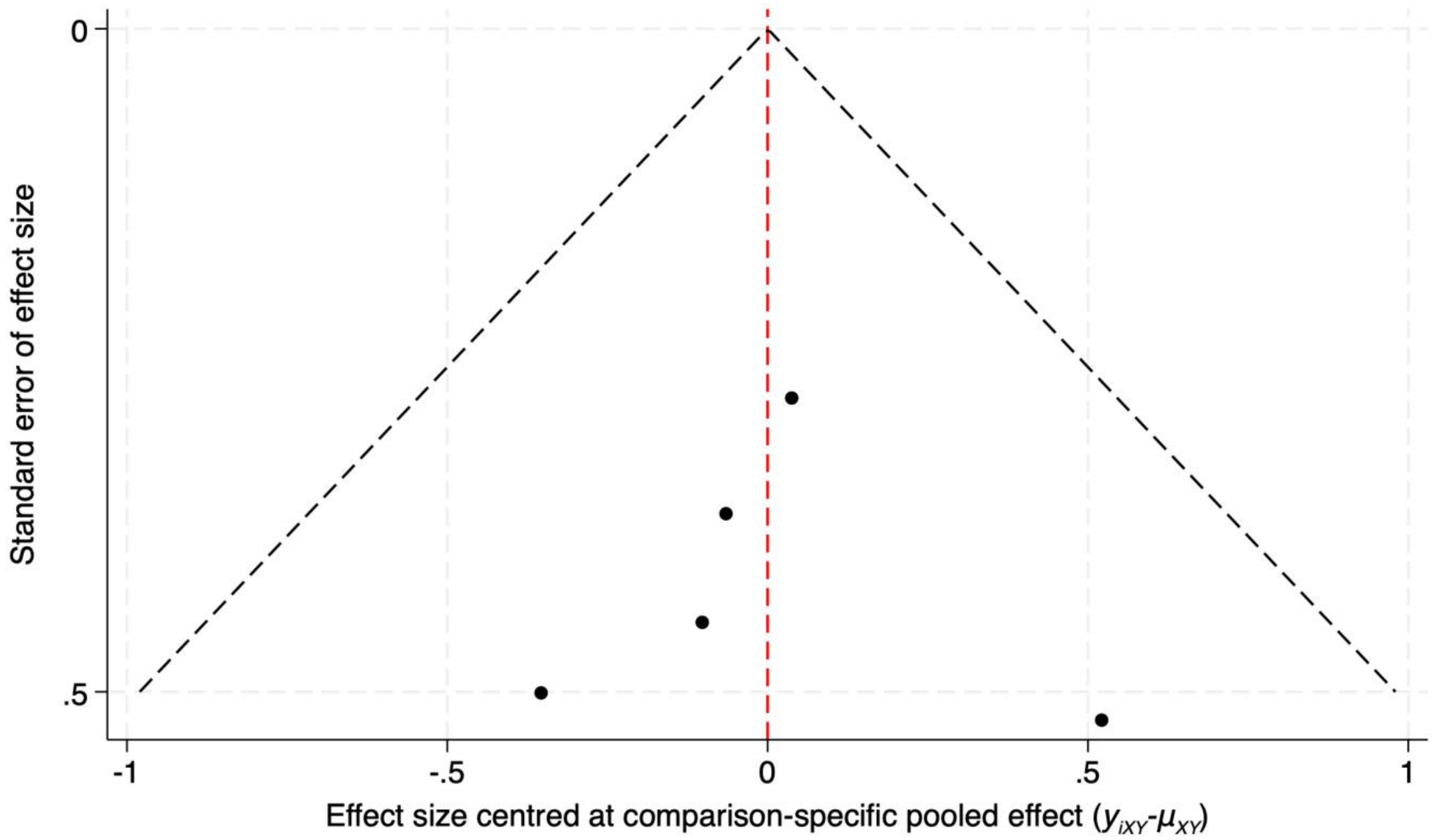

Egger's test t= 0.09 P=0.935

Appendix 13: Sensitivity analyses

Table S13.1: Sensitivity analyses of outcomes

Intervention	48-h DOMS				48-h Muscle strength				48-h CK			48-h CRP		
	Main analysis	Excluding acute protocols	Excluding pre-exercise-only supplementation	Excluding post-exercise -only supplementation	Main analysis	Excluding acute protocols	Excluding isokinetic total work	Isometric strength-only analysis	Main analysis	Excluding acute protocols	Excluding pre-exercise-only supplementation	Main analysis	Excluding acute protocols	Excluding pre-exercise-only supplementation
Tart cherry	-0.39(-0.70,-0.07)	-0.38(-0.68, -0.09)	-0.38 (-0.67, -0.09)	-0.44 (-0.80, -0.08)	0.39(-0.07,1.13)	0.39 (-0.07,0.85)	0.51(-0.02,1.04)	0.59(-0.02,1.20)	-0.21(-0.54,0.12)	-0.21(-0.50,0.08)	-0.21(-0.50,0.08)	-0.24 (-0.63,0.15)	-0.24 (-0.65,0.18)	-0.24 (-0.65,0.18)
Pomegranate	-0.68(-1.34,-0.02)	-0.28 (-0.89,0.33)	-0.28 (-0.87,0.31)	-0.71 (-1.42, -0.01)	0.16(-0.52,0.84)	0.16 (-0.52,0.84)	0.17(-0.54,0.88)	0.69(-0.37,1.74)	-0.25(-0.84,0.34)	0.07(-0.37,0.50)	0.07(-0.37,0.50)	-0.25 (-0.64,0.15)	-0.28 (-0.74,0.18)	-0.28 (-0.74,0.18)
Tea extracts	-0.13(-0.89,0.62)	-0.16 (-0.84,0.52)	-0.17 (-0.83,0.49)	-0.12 (-0.93,0.69)	NA	NA	NA	NA	0.57(-0.07,1.21)	0.57 (0.03,1.1)	0.44(-0.31,1.20)	NA	NA	NA
Blueberry	0.34(-0.37,1.04)	0.50 (-0.27,1.28)	0.35 (-0.25,0.95)	0.33 (-0.43,1.09)	0.10(-1.03,1.23)	NA	0.10(-1.08,1.28)	0.10(-1.07,1.27)	0.37(-0.37,1.12)	0.37 (0.19,0.9)	0.37(-0.19,0.94)	NA	NA	NA
Blackcurrant	0.13(-0.69,0.96)	0.14 (-0.62,0.89)	0.14 (-0.60,0.88)	0.13 (-0.75,1.00)	NA	NA	NA	NA	-0.91(-2.07,0.25)	-0.91 (-1.96,0.14)	-0.91(-1.96,0.14)	NA	NA	NA
Cocoa flavanols	0.06(-0.79,0.90)	0.05 (-0.73,0.83)	0.05 (-0.71,0.82)	0.06 (-0.84,0.95)	0.14(-0.72,1.00)	0.14 (-0.72,1.00)	0.14(-0.75,1.03)	0.14(-0.75,1.03)	-0.07(-0.85,0.71)	-0.05(-0.75,0.64)	-0.05(-0.75,0.64)	0.34 (-0.55,1.22)	0.34 (-0.57,1.25)	0.34 (-0.57,1.25)
Grape	0.05 (-0.88,0.99)	0.05 (-0.76,0.86)	0.05 (-0.73,0.83)	0.05 (-0.96,1.07)	0.18(-0.77,1.13)	0.18 (-0.77,1.13)	0.18(-0.82,1.18)	0.18(-0.82,1.17)	NA	NA	NA	NA	NA	NA
Mixed polyphenols	-0.10(-1.19,1.00)	-0.10 (-1.08,0.89)	-0.10 (-1.06,0.87)	-0.10 (-1.26,1.07)	NA	NA	NA	NA	-0.00(-1.00,1.00)	-0.00 (-0.88,0.88)	-0.05(-0.75,0.64)	-0.49 (-1.39,0.40)	-0.49 (-1.41,0.43)	-0.49 (-1.41,0.43)

Note: Some sensitivity analyses were not included because the number of available studies was insufficient or the network could not be estimated, provided that this accurately reflects the actual analysis conducted.

Table S13.2: Sensitivity analyses for 48-h DOMS according to comparator classification

Intervention	48-h DOMS				
	Main analysis	Excluding Sensory matched but nutritionally non-equivalent comparator and Nutrient-mismatched control	Excluding Partially matched or unclear comparator and Sensory matched but nutritionally non-equivalent comparator	Excluding Protein, milk, or smoothie based control and Nutrient-mismatched control	Excluding Energy-containing placebo, Protein, milk, or smoothie based control and Nutrient-mismatched control
Tart cherry	-0.39 (-0.70,-0.07)	-0.38 (-0.73,-0.03)	-0.36 (-0.65, -0.07)	-0.39 (-0.75,-0.02)	-0.03 (-0.43, 0.37)
Pomegranate	-0.68 (-1.34,-0.02)	-0.76 (-1.45,-0.08)	-0.28 (-0.90, 0.34)	-0.79 (-1.51,-0.06)	-2.66 (-3.98, -1.34)
Tea extracts	-0.13 (-0.89,0.62)	-0.11 (-0.93,0.71)	-0.15 (-0.83, 0.52)	-0.10 (-0.97, 0.76)	-0.18 (-0.79, 0.42)
Blueberry	0.34 (-0.37,1.04)	0.33 (-0.45, 1.11)	0.51 (-0.28, 1.29)	0.51 (-0.58, 1.59)	NA
Blackcurrant	0.13 (-0.69,0.96)	0.13 (-0.76, 1.02)	0.14 (-0.63, 0.90)	0.13 (-0.80, 1.06)	0.48 (-0.48, 1.43)
Cocoa flavanols	0.06 (-0.79,0.90)	0.06 (-0.85, 0.97)	0.05 (-0.73, 0.84)	-0.03 (-1.31, 1.24)	NA
Grape	0.05 (-0.88,0.99)	0.05 (-0.99, 1.10)	0.05 (-0.77, 0.88)	0.05 (-1.06, 1.17)	NA
Mixed polyphenols	-0.10 (-1.19,1.00)	-0.10 (-1.28, 1.09)	-0.10 (-1.10, 0.90)	-0.10 (-1.35,1.15)	-0.10 (-1.01, 0.81)

Table S13.3: Sensitivity analyses for 48-h muscle strength according to comparator classification

Intervention	48-h Muscle strength			
	Main analysis	Excluding Partially matched or unclear comparator	Excluding Protein, milk, or smoothie based control	Excluding Energy-containing placebo and Protein, milk, or smoothie based control
Tart cherry	0.39<br>(-0.07, 1.13)	0.40<br>(-0.07, 0.87)	0.40<br>(-0.09, 0.89)	0.12<br>(-0.40, 0.64)
Pomegranate	0.16<br>(-0.52, 0.84)	0.17<br>(-0.53, 0.86)	0.17<br>(-0.56, 0.91)	-0.25<br>(-0.80, 0.29)
Tea extracts	NA	NA	NA	NA
Blueberry	0.10<br>(-1.03, 1.23)	NA	NA	NA
Blackcurrant	NA	NA	NA	NA
Cocoa flavanols	0.14<br>(-0.72, 1.00)	0.14<br>(-0.74, 1.02)	0.38<br>(-0.83, 1.59)	NA
Grape	0.18<br>(-0.77, 1.13)	0.18<br>(-0.81, 1.16)	0.18<br>(-0.86, 1.21)	NA
Mixed polyphenols	NA	NA	NA	NA

Table S13.4: Sensitivity analyses for 48-h CK according to comparator classification

Intervention	48-h CK				
	Main analysis	Excluding Sensory matched but nutritionally non-equivalent comparator	Excluding Partially matched or unclear comparator and Sensory matched but nutritionally non-equivalent comparator	Excluding Protein, milk, or smoothie based control and Nutrient-mismatched control	Excluding Energy-containing placebo, Protein, milk, or smoothie based control and Nutrient-mismatched control
Tart cherry	-0.21(-0.54,0.12)	-0.21(-0.62, 0.21)	-0.21(-0.50, 0.09)	-0.21(-0.63, 0.21)	-0.24(-1.15, 0.67)
Pomegranate	-0.25(-0.84,0.34)	-0.37(-1.02, 0.27)	0.07(-0.36, 0.51)	-0.38(-1.03, 0.28)	-1.01(-2.16, 0.15)
Tea extracts	0.57(-0.07,1.21)	0.57(-0.21, 1.35)	0.57(0.03, 1.10)	0.57(-0.22, 1.36)	NA
Blueberry	0.37(-0.37,1.12)	0.38(-0.61, 1.36)	0.38(-0.19, 0.94)	0.38(-0.62, 1.37)	NA
Blackcurrant	-0.91(-2.07,0.25)	-0.92(-2.23, 0.38)	-0.92(-1.96, 0.11)	-0.92(-2.24, 0.39)	NA
Cocoa flavanols	-0.07(-0.85,0.71)	-0.10(-1.00, 0.80)	-0.06(-0.75, 0.63)	0.26(-0.94, 1.46)	NA
Grape	NA	NA	NA	NA	NA
Mixed polyphenols	-0.00(-1.00,1.00)	-0.00(-1.19, 1.19)	-0.00(-0.88, 0.88)	-0.00(-1.20, 1.20)	-0.00(-1.64, 1.64)

Table S13.5: Sensitivity analyses for 48-h CRP according to comparator classification

Intervention	48-h CRP		
	Main analysis	Excluding Partially matched or unclear comparator and Sensory matched but nutritionally non-equivalent comparator	Excluding Energy-containing placebo, Protein, milk, or smoothie based control and Nutrient-mismatched control
Tart cherry	-0.24 (-0.63,0.15)	-0.23 (-0.64, 0.17)	-0.71(-1.40, -0.03)
Pomegranate	-0.25 (-0.64,0.15)	-0.28 (-0.75, 0.18)	-0.24 (-0.72, 0.23)
Tea extracts	NA	NA	NA
Blueberry	NA	NA	NA
Blackcurrant	NA	NA	NA
Cocoa flavanols	0.34 (-0.55,1.22)	0.34 (-0.57, 1.25)	NA
Grape	NA	NA	NA
Mixed polyphenols	-0.49 (-1.39,0.40)	-0.49 (-1.42, 0.43)	-0.49 (-1.38, 0.40)

Appendix 14: Subgroup analysis of training status

Subgroup analyses were conducted according to participants' training status, categorised as trained participants and untrained or recreationally active participants.

Figure S14.1: Forest plot of 48-h DOMS in trained participants. Negative SMDs favour plant-derived supplements. Note: The placebo node pools all eligible control conditions, including matched placebo and vehicle-matched controls.

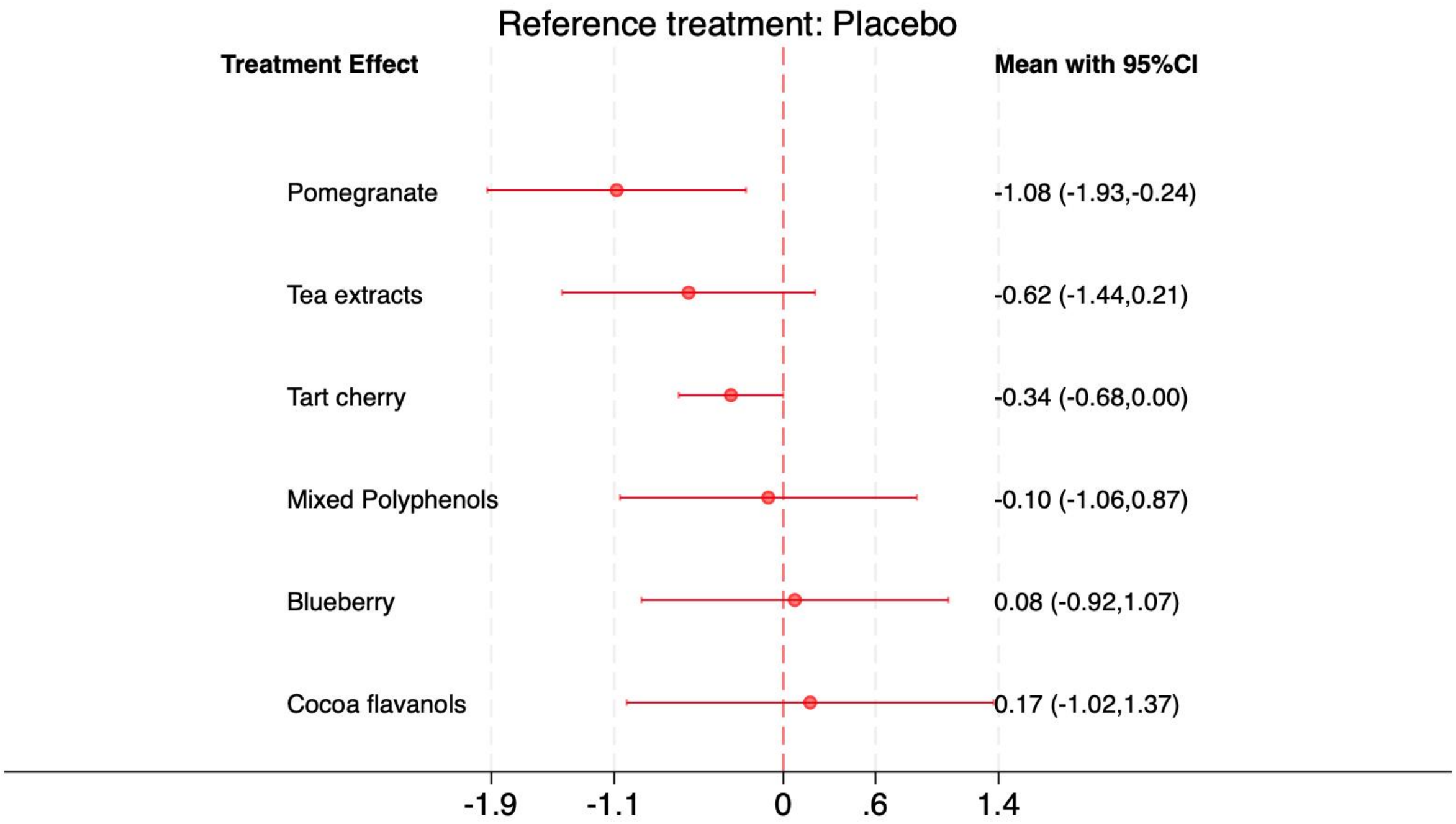

Table S14.1: League table of DOMS at 48 h Post-Exercise in trained participants.

Tart cherry						
0.74 (-0.14,1.63)	Pomegranate					
0.27 (-0.62,1.17)	-0.47 (-1.65,0.71)	Tea extracts				
-0.42 (-1.47,0.64)	-1.16 (-2.47,0.15)	-0.69 (-1.99,0.60)	Blueberry			
-0.52 (-1.76,0.73)	-1.26 (-2.72,0.20)	-0.79 (-2.24,0.66)	-0.10 (-1.66,1.46)	Cocoa flavanols		
-0.24 (-1.27,0.78)	-0.99 (-2.27,0.29)	-0.52 (-1.79,0.75)	0.17 (-1.22,1.56)	0.27 (-1.26,1.81)	Mixed Polyphenols	
-0.34 (-0.68,-0.00)	-1.08 (-1.93,-0.24)	-0.62 (-1.44,0.21)	0.08 (-0.92,1.07)	0.17 (-1.02,1.37)	-0.10 (-1.06,0.87)	Placebo

Note: Values are SMDs with 95% CIs. Negative values indicate lower DOMS and favour the treatment listed in the column over the treatment listed in the row. The placebo node pools all eligible control conditions, including matched placebo and vehicle-matched controls.

Figure S14.2: Forest plot of 48-h DOMS in untrained or recreationally active participants. Negative SMDs favour plant-derived supplements. The placebo node pools all eligible control conditions, including matched placebo and vehicle-matched controls.

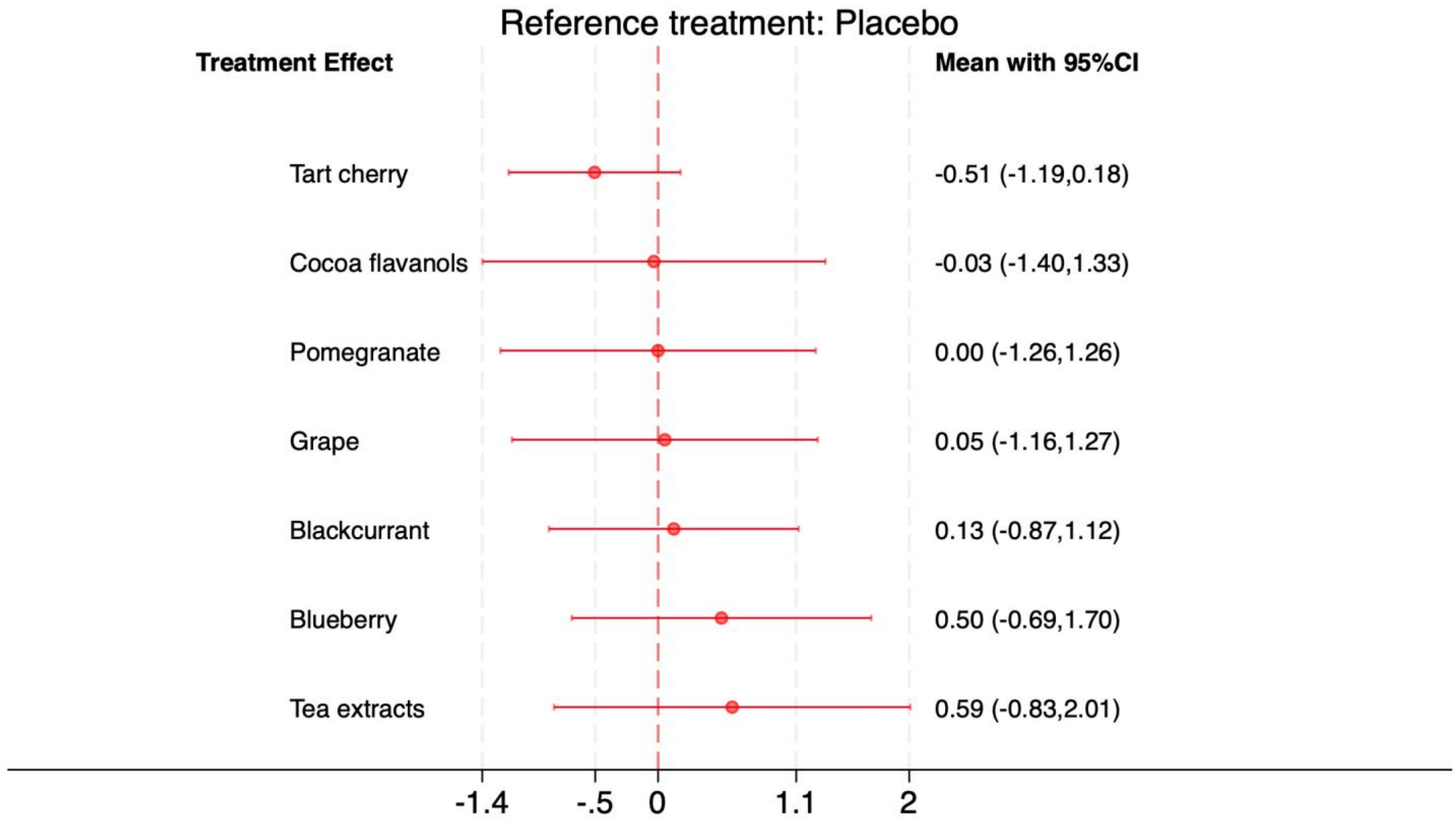

Table S14.2: League table of DOMS at 48 h post-exercise in untrained or recreationally active participants.

Tart cherry							
-0.51 (-1.94,0.92)	Pomegranate						
-1.10 (-2.67,0.48)	-0.59 (-2.48,1.30)	Tea extracts					
-1.01 (-2.39,0.36)	-0.50 (-2.24,1.23)	0.09 (-1.77,1.94)	Blueberry				
-0.63 (-1.84,0.57)	-0.13 (-1.73,1.48)	0.46 (-1.27,2.20)	0.38 (-1.17,1.93)	Blackcurrant			
-0.47 (-2.00,1.05)	0.03 (-1.82,1.89)	0.62 (-1.35,2.59)	0.54 (-1.28,2.35)	0.16 (-1.53,1.85)	Cocoa flavanols		
-0.56 (-1.96,0.84)	-0.05 (-1.80,1.70)	0.54 (-1.33,2.41)	0.45 (-1.25,2.16)	0.07 (-1.50,1.64)	-0.09 (-1.92,1.74)	Grape	
-0.51 (-1.19,0.18)	-0.00 (-1.26,1.26)	0.59 (-0.83,2.01)	0.50 (-0.69,1.70)	0.13 (-0.87,1.12)	-0.03 (-1.40,1.33)	0.05 (-1.16,1.27)	Placebo

Note: Values are SMDs with 95% CIs. Negative values indicate lower DOMS and favour the treatment listed in the column over the treatment listed in the row. The placebo node pools all eligible control conditions, including matched placebo and vehicle-matched controls.

Figure S14.3: Forest plot of 48-h muscle strength/torque in trained participants. Positive SMDs favour plant-derived supplements. The placebo node pools all eligible control conditions, including matched placebo and vehicle-matched controls.

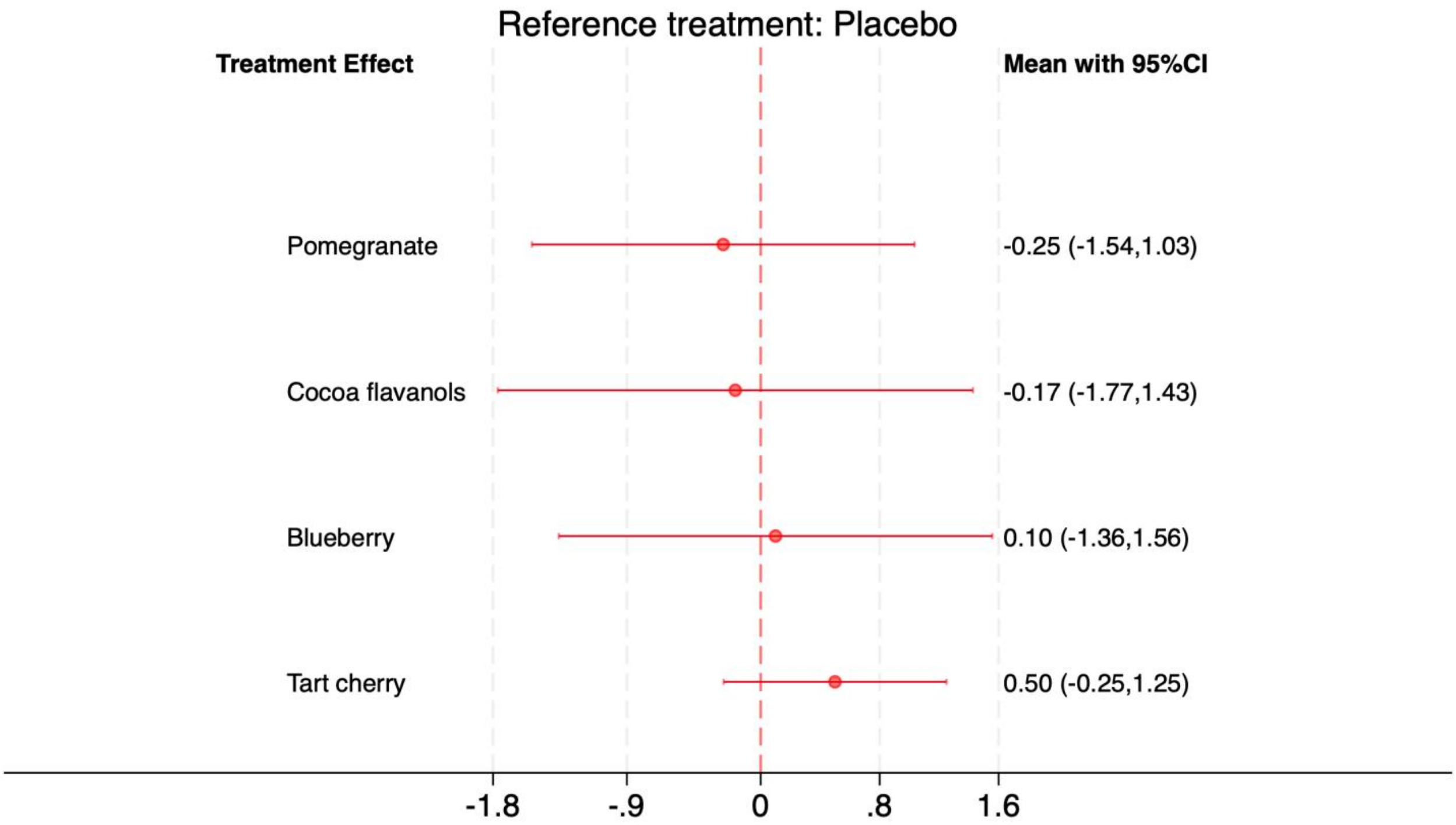

Table S14.3: League table of muscle strength/torque at 48 h post-exercise in trained participants.

Tart cherry				
0.75 (-0.74,2.24)	Pomegranate			
0.40 (-1.24,2.04)	-0.35 (-2.30,1.59)	Blueberry		
0.67 (-1.09,2.44)	-0.08 (-2.13,1.97)	0.27 (-1.89,2.43)	Cocoa flavanols	
0.50 (-0.25,1.25)	-0.25 (-1.54,1.03)	0.10 (-1.36,1.56)	-0.17 (-1.77,1.43)	Placebo

Note: Values are SMDs with 95% CIs. Positive values indicate better muscle strength/torque recovery and favour the treatment listed in the column over the treatment listed in the row. The placebo node pools all eligible control conditions, including matched placebo and vehicle-matched controls.

Figure S14.4: Forest plot of 48-h muscle strength/torque in untrained or recreationally active participants. Positive SMDs favour plant-derived supplements. The placebo node pools all eligible control conditions, including matched placebo and vehicle-matched controls.

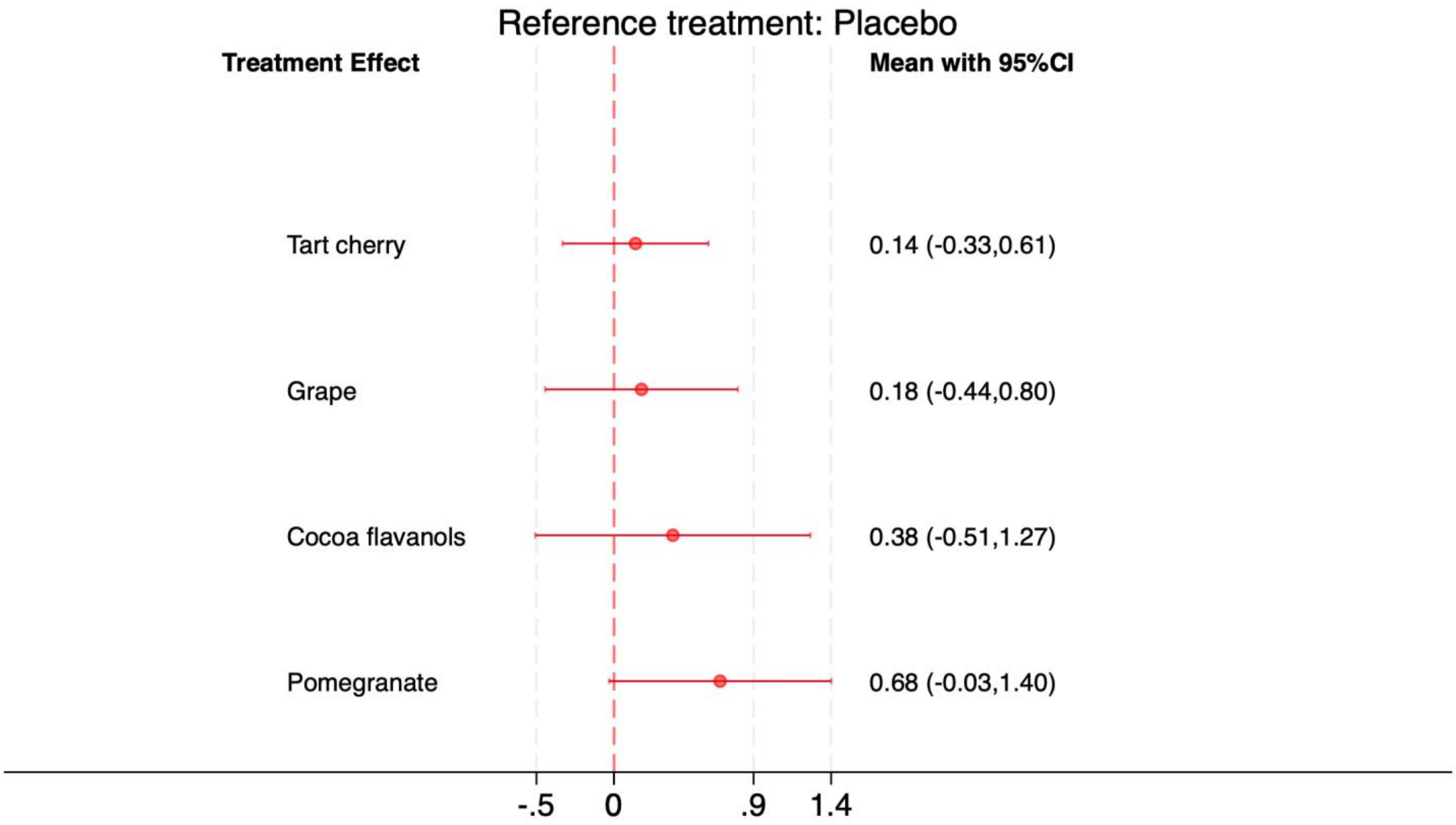

Table S14.4: League table of muscle strength/torque at 48 h post-exercise in untrained or recreationally active participants.

Tart cherry				
-0.55 (-1.40,0.31)	Pomegranate			
-0.24 (-1.24,0.76)	0.31 (-0.83,1.45)	Cocoa flavanols		
-0.04 (-0.82,0.74)	0.51 (-0.44,1.46)	0.20 (-0.88,1.28)	Grape	
0.14 (-0.33,0.61)	0.68 (-0.03,1.40)	0.38 (-0.51,1.27)	0.18 (-0.44,0.80)	Placebo

Note: Values are SMDs with 95% CIs. Positive values indicate better muscle strength/torque recovery and favour the treatment listed in the column over the treatment listed in the row. The placebo node pools all eligible control conditions, including matched placebo and vehicle-matched controls.

Figure S14.5: Forest plot of 48-h CK in trained participants. Negative SMDs favour plant-derived supplements. The placebo node pools all eligible control conditions, including matched placebo and vehicle-matched controls.

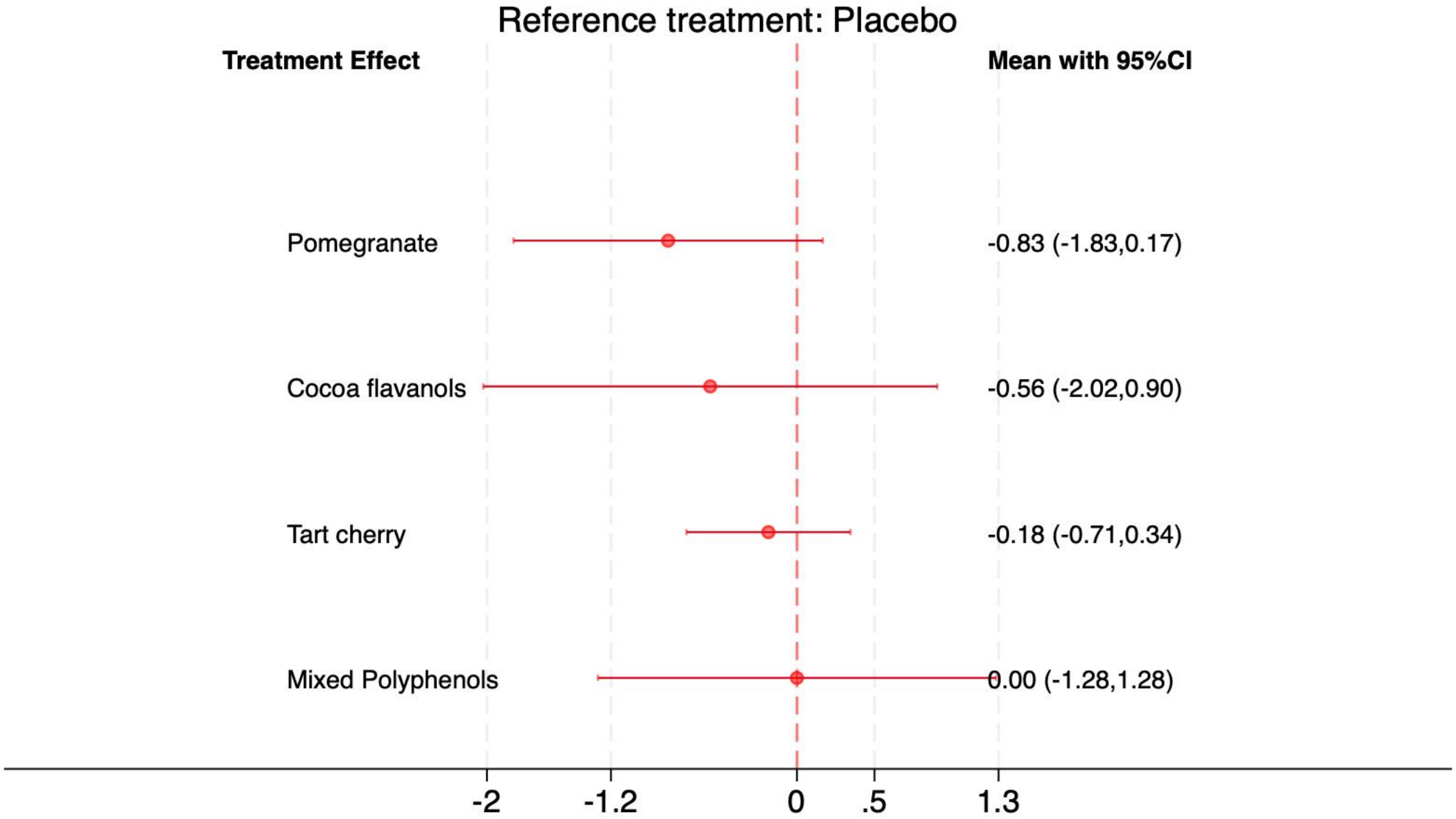

Table S14.5: League table of CK at 48 h post-exercise in trained participants.

Tart cherry				
0.65 (-0.49,1.78)	Pomegranate			
0.38 (-1.18,1.93)	-0.27 (-2.04,1.50)	Cocoa flavanols		
-0.18 (-1.57,1.20)	-0.83 (-2.46,0.80)	-0.56 (-2.51,1.39)	Mixed Polyphenols	
-0.18 (-0.71,0.34)	-0.83 (-1.83,0.17)	-0.56 (-2.02,0.90)	-0.00 (-1.28,1.28)	Placebo

Note: Values are SMDs with 95% CIs. Negative values indicate lower CK and favour the treatment listed in the column over the treatment listed in the row. The placebo node pools all eligible control conditions, including matched placebo and vehicle-matched controls.

Figure S14.6: Forest plot of 48-h CKin untrained or recreationally active participants. Negative SMDs favour plant-derived supplements. The placebo node pools all eligible control conditions, including matched placebo and vehicle-matched controls.

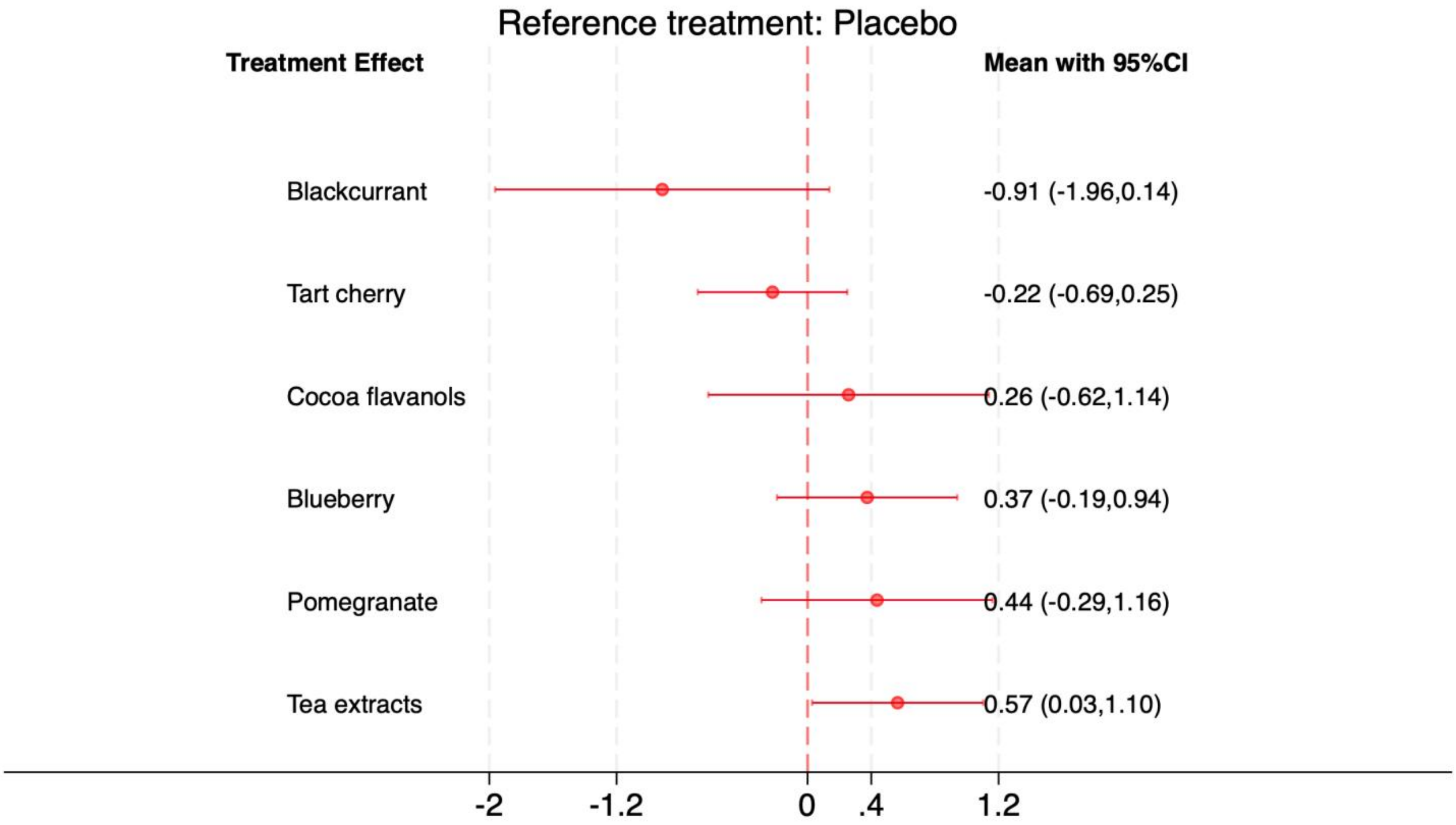

Table S14.6: League table of CK at 48 h post-exercise in untrained or recreationally active participants.

Tart cherry						
-0.66 (-1.52 ,0.21)	Pomegranate					
-0.79 (-1.50,- 0.07)	-0.13 (-1.03, 0.77)	Tea extracts				
-0.59 (-1.33, 0.14)	0.06 (-0.86, 0.98)	0.19 (-0.59, 0.97)	Blueberry			
0.69 (-0.46, 1.84)	1.35 (0.07, 2.63)	1.48 (0.30, 2.66)	1.29 (0.09, 2.48)	Blackcurrant		
-0.48 (-1.48,0.52)	0.18 (-0.96, 1.32)	0.31 (-0.72, 1.34)	0.12 (-0.93, 1.16)	-1.17 (-2.54, 0.20)	Cocoa flavanols	
-0.22 (-0.69, 0.25)	0.44 (-0.29, 1.16)	0.57 (0.03, 1.10)	0.37 (-0.19, 0.94)	-0.91 (-1.96, 0.14)	0.26 (-0.62, 1.14)	Placebo

Note: Values are SMDs with 95% CIs. Negative values indicate lower CK and favour the treatment listed in the column over the treatment listed in the row. The placebo node pools all eligible control conditions, including matched placebo and vehicle-matched controls.

Figure S14.7: Forest plot of 48-h CRP in trained participants. Negative SMDs favour plant-derived supplements. The placebo node pools all eligible control conditions, including matched placebo and vehicle-matched controls.

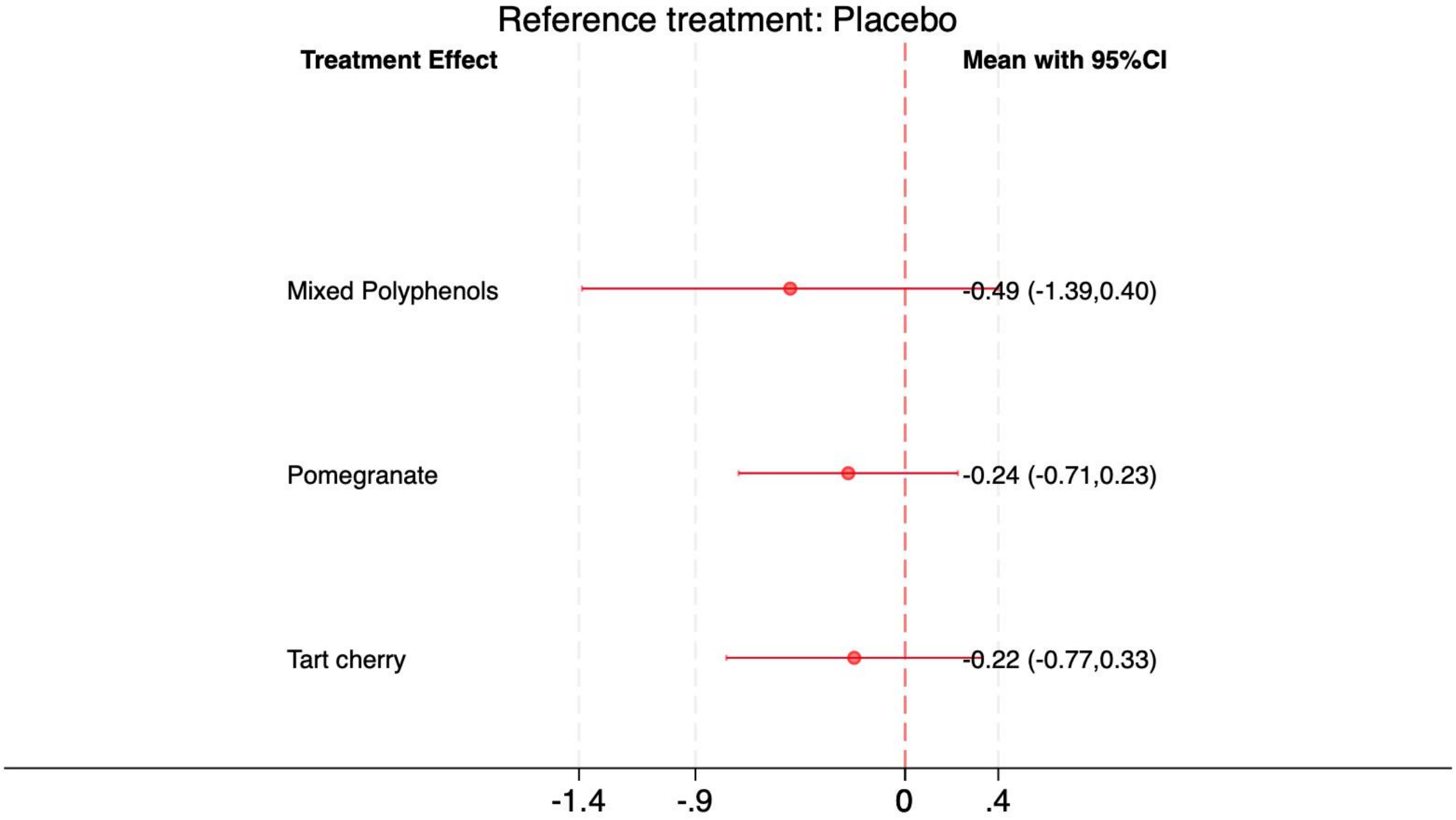

Table S14.7: League table of CRP at 48 h post-exercise in trained participants.

Tart cherry			
0.08 (-0.70,0.85)	Pomegranate		
0.27 (-0.77,1.32)	0.25 (-0.76,1.26)	Mixed Polyphenols	
-0.22 (-0.77,0.33)	-0.30 (-0.84,0.25)	-0.49 (-1.39,0.40)	Placebo

Note: Values are SMDs with 95% CIs. Negative values indicate lower CRP and favour the treatment listed in the column over the treatment listed in the row. The placebo node pools all eligible control conditions, including matched placebo and vehicle-matched controls.

Figure S14.8: Forest plot of 48-h CRP in untrained or recreationally active participants. Negative SMDs favour plant-derived supplements. The placebo node pools all eligible control conditions, including matched placebo and vehicle-matched controls.

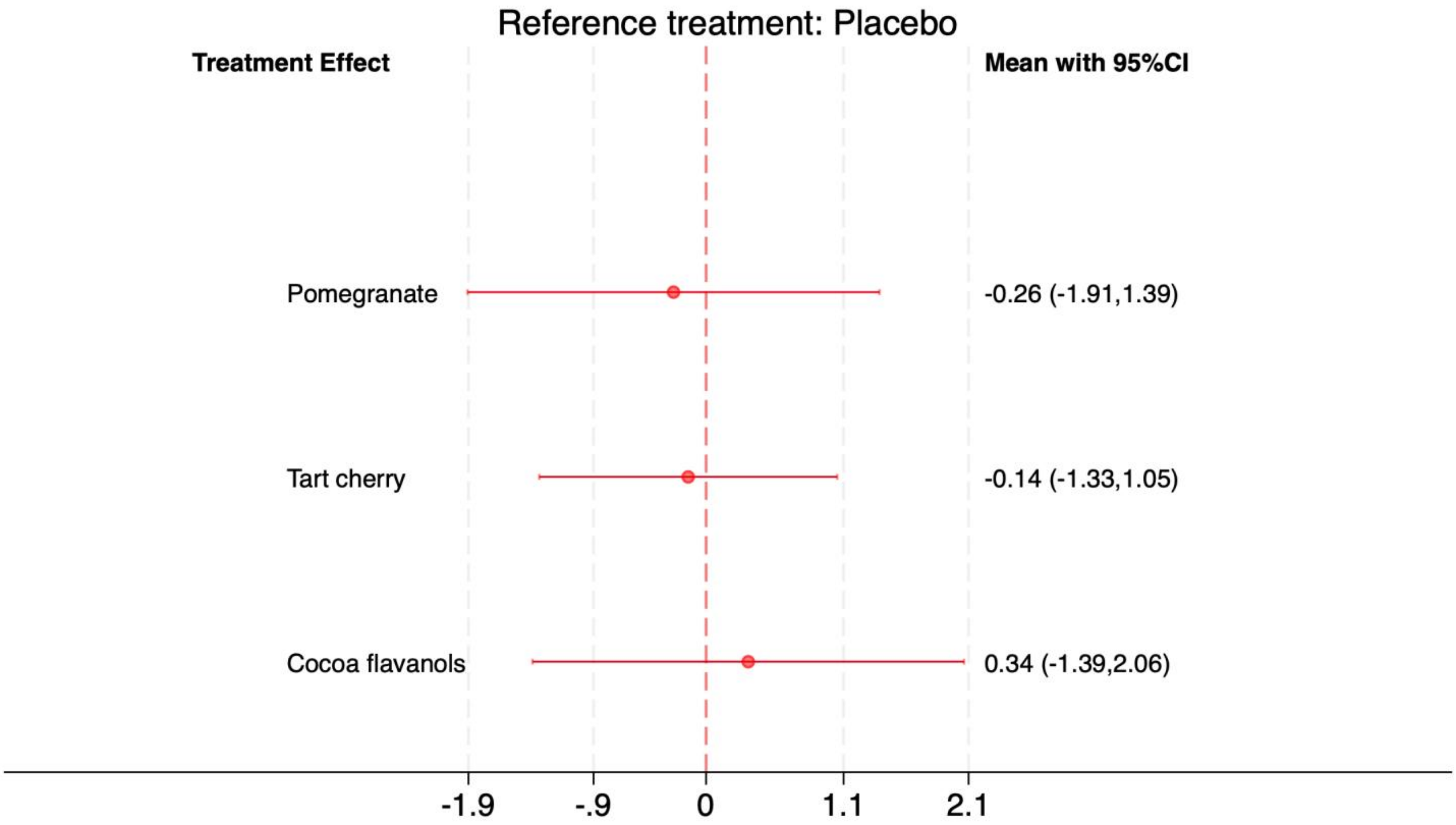

Table S14.8: League table of CRP at 48 h post-exercise in untrained or recreationally active participants.

Tart cherry			
0.12 (-1.92,2.15)	Pomegranate		
-0.48 (-2.58,1.62)	-0.60 (-2.98,1.79)	Cocoa flavanols	
-0.14 (-1.33,1.05)	-0.26 (-1.91,1.39)	0.34 (-1.39,2.06)	Placebo

Note: Values are SMDs with 95% CIs. Negative values indicate lower CRP and favour the treatment listed in the column over the treatment listed in the row. The placebo node pools all eligible control conditions, including matched placebo and vehicle-matched controls.
